# Pharmacophore modelling of vanillin derivatives, favipiravir, chloroquine, hydroxychloroquine, monolaurin and tetrodotoxin as M^Pro^ inhibitors of severe acute respiratory syndrome coronavirus-2 (SARS-CoV-2)

**DOI:** 10.1186/s13104-020-05379-6

**Published:** 2020-11-11

**Authors:** Woon Yi Law, Mohd Razip Asaruddin, Showkat Ahamd Bhawani, Samsur Mohamad

**Affiliations:** Faculty of Resource Science and Technology, Universiti Malaysia Sarawak, 94300 Kota Samarahan, Sarawak, Malaysia

**Keywords:** Coronavirus, Pharmacophore modelling, Vanillin, Favipiravir, Chloroquine, Hydroxychloroquine, Monolaurin, Tetrodotoxin

## Abstract

**Objectives:**

The aim of this study was to use Ligand-based pharmacophore modelling approach for four established antiviral drugs, namely remdesivir, lopinavir, ritonavir and hydroxychloroquine for COVID-19 inhibitors as training sets. In this study Twenty vanillin derivatives together with monolaurin and tetrodotoxin were used as test sets to evaluate as potential SARS-CoV-2 inhibitors. The Structure-based pharmacophore modelling approach was also performed using 5RE6, 5REX and 5RFZ in order to analyse the binding site and ligand–protein complex interactions.

**Results:**

The pharmacophore modelling mode of 5RE6 displayed two Hydrogen Bond Acceptors (HBA) and one Hydrophobic (HY) interaction. Besides, the pharmacophore model of 5REX showed two HBA and two HY interactions. Finally, the pharmacophore model of 5RFZ showed three HBA and one HY interaction. Based on ligand-based approach, 20 Schiff-based vanillin derivatives, showed strong M^Pro^ inhibition activity. This was due to their good alignment and common features to PDB-5RE6. Similarly, monolaurin and tetrodotoxin displayed some significant activity against SARS-CoV-2. From structure-based approach, vanillin derivatives (**1**) to (**12**) displayed some potent M^Pro^ inhibition against SARS-CoV-2. Favipiravir, chloroquine and hydroxychloroquine also showed some significant M^Pro^ inhibition.

## Introduction

In the early twenty-first century, an outbreak of coronaviruses has been causing a number of diseases. Coronavirus belong to the family of *Coronaviridae*. Coronaviruses are enveloped, positive-sense, single stranded RNA viruses, with their size approximately 27 to 34 kilobases [1, 2]. Out of numerous coronaviruses, seven types of coronaviruses are known for their ability to cause infections and respiratory illnesses in humans [3]. Four types of coronaviruses: 229E, OC43, NL63, and HKU1 are associated with the symptoms of common cold [4]. Another two types of coronaviruses, which are severe acute respiratory syndrome coronavirus (SARS-CoV) and Middle East respiratory syndrome coronavirus (MERS-CoV) were the causal agents of the outbreak of severe acute respiratory syndrome (SARS) in 2002–2003 and Middle East respiratory syndrome in 2012, respectively [4]. Both coronaviruses had accumulated more than 10,000 cases in the past, with 10% death cases from SARS and 37% death cases from MERS [5, 6].

In December 2019, a novel strain of coronavirus was identified at Wuhan, China, which led to a series of pneumonia cases [7]. Formerly named as 2019 novel coronavirus (2019-nCoV) by the World Health Organization (WHO), the coronavirus was later named as severe acute respiratory syndrome coronavirus-2 (SARS-CoV-2) due to the genetic resemblance of about 80% with SARS-CoV [7, 8]. The disease was named as coronavirus disease 2019 (COVID-19) by WHO [7]. On 30th of January 2020, the outbreak of COVID-19 was declared a Public Health Emergency of International Concern (PHEIC), and consequently on 11th of March 2020, an official pandemic was declared [9, 10]. The situation is further aggravated due to the absence of specific medicinal drugs or vaccines that are licensed or approved by the U.S. Food and Drug Administration (FDA) for the treatment of COVID-19 [11].

Coronaviruses are divided into four genera, namely α-CoVs, β-CoVs, γ-CoVs and δ-CoVs [12]. Among these, the α-CoVs and β-CoVs possess ability to infect mammals, whereas the γ-CoVs and δ-CoVs can infect both birds and mammals [13]. The three coronaviruses, namely, SARS-CoV, MERS-CoV and SARS-CoV-2 belong to β-CoVs [14]. There are four types of structural proteins found on the surface of coronaviruses: spike (S) protein, envelope (E) protein, membrane (M) protein, and nucleocapsid (N) protein [15]. The attachment and entry of SARS-CoV-2 into the host cell is facilitated by the S proteins, particularly to the angiotensin converting enzyme 2 (ACE2) receptors of human cells due to great affinity [16]. Therefore, therapies to control the activities of SARS-CoV-2, such as preventing viral RNA synthesis, viral replication or blocking the binding of virus are efforts that researchers are putting on to take care of the issue. In coronaviruses, one of the drug target that is being focused by chemists is the main protease (M^Pro^) due to its important role in processing viral polyproteins translated from the viral RNA and controlling replicase complex activity [17, 18]. The activity of M^Pro^ should be inhibited to control and prevent the viral action of SARS-CoV-2. In recent times, vanillin derivatives, which are Schiff-based vanillin with primary amines, have been utilised by researchers for different research purposes. From previous reported research, Schiff-based vanillin derivatives have been tested as neuraminidase inhibitors of influenza virus [19]. In addition to this, vanillin and its derivatives had also been tested for their antifungal activity against the human fungal pathogen, *Cryptococcus neoformans* [20]. Hence in this study, a series of Schiff-based vanillin derivatives will be tested as potent M^Pro^ inhibitors of SARS-CoV-2 by means of ligand-based and structure-based pharmacophore modelling in computer-aided drug design (CADD).

As the world is combating with the viral disease, the first antiviral drug named Favilavir has been approved by the National Medical Products Administration of China [21]. Despite the fact that Favilavir is yet to be approved by the U.S. Food and Drug Administration (FDA), the ability of the drug to inhibit the action of the RNA-dependent RNA polymerase (RdRp) has been proven [21, 22]. The chemical compound, favipiravir, was known as a broad spectrum antiviral drug that showed inhibition against influenza virus [23]. The Ministry of Science and Technology of China reported that patients from Shenzhen which received Favilavir treatment were tested negative for the coronavirus after 4 days being tested positive, as well as 91% improvement in their lung conditions [22]. From this perspective, in silico screening in CADD will be performed as well to test the potent ability of favipiravir as M^Pro^ inhibitors of SARS-CoV-2 (Additional file 1).

Chloroquine is a medicinal drug known for its antimalarial activity used to treat malaria, and used as prophylaxis [24, 25]. Recently two research teams have reported the in vitro antiviral study of chloroquine against the growth of SARS-CoV-2, which was also further confirmed by providing medication to about 100 COVID-19 patients [26, 27]. The study is further affirmed by reporting the antiviral activity of chloroquine in interfering SARS-CoV from binding ACE2 receptors through decreasing the terminal glycosylation of ACE2 receptors, therefore inhibiting virus replication [28]. Simultaneously, one of the recent study found out that the S protein of SARS-CoV-2 is very identical to that of SARS-CoV, which can bind to the ACE2 receptors [16]. A derivative of chloroquine, namely, hydroxychloroquine, was reported to be more soluble and less toxic than chloroquine [25]. Similar to chloroquine, hydroxychloroquine is also a potential COVID-19 pharmacological agent for its in vitro antiviral activity being tested against SARS-CoV-2 [29]. The Chinese Clinical Trial Registry performed seven clinical trial registries on hydroxychloroquine to determine its efficacy in treating COVID-19 [29]. However up to now, there are still not enough clinical evidence to confirm the effectivity of hydroxychloroquine as anti-SARS-CoV-2 drug.

Coconut oil and its derivatives have been long reported for their antiviral and antibacterial activity, particularly lauric acid and monolaurin were utilised as feed supplements in farm animals [30]. It was also reported that lauric acid and its derivative, monolaurin, exhibit potential in vitro antiviral activity against SARS-CoV-2 [31]. This was reported that by elaborating the potential of coconut oil as antiviral agent against COVID-19 is based on three mechanisms, disintegration of the virus membrane, inhibition of virus maturation and prevention of binding of viral proteins to the host cell membrane [31]. Tetrodotoxin, a potent neurotoxin, is mostly found in pufferfish [32]. Tetrodotoxin was known as sodium channel blocker, preventing messages to be delivered by the nervous system [33]. Despite the fact being 1200 times more toxic than cyanide, the analgesic activity of tetrodoxin was applied by researchers in discovery of drugs for pain relief, for example in severe cancer [34]. Therefore, the potent antiviral activity of chloroquine, hydroxychloroquine, monolaurin and tetrodotoxin will be tested via in silico screening in CADD.

## Main text

### Methodology

The pharmacophore modelling studies were carried out using Ligand Scout 4.3 software, a powerful structure and ligand based pharmacophore model generator by Inte:Ligand. The 3D molecular graphics were generated using ligand-based and structure-based approach. All chemical molecular structures were drawn from minimized function of ChemSpider and MolView 2.4 and were then being converted into MOL file format. All training sets were imported into the ligand-based perspective of LigandScout 4.3.

#### Ligand-based pharmacophore modelling

Four antiviral drugs (Table 1) used for the treatment of COVID-19 were used as training sets for the generation of pharmacophore model, namely remdesivir, lopinavir, ritonavir and hydroxychloroquine [29, 30, 32].

**Table 1:**
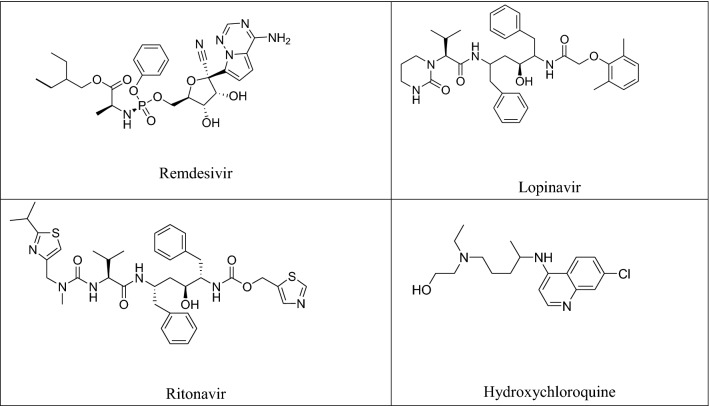
List of antiviral drugs

A group of 20 primary amine Schiff-based vanillin derivatives (Table 2) were imported as test sets, namely vanillin associated with methyl-6-aminopyridine-3-carboxylate **(1)**, sepiapterin **(2)**, 6-aminopyridine-3-carboxylic acid **(3)**, 6-aminopyridine-2-carboxylic acid **(4)**, pemoline **(5)**, α-phenylglycine **(6)**, 2-amino-4-hydroxy-3-methylpentanoic acid **(7)**, 4-hydroxyphenylglycine **(8)**, β-homoserine **(9)**, allylglycine **(10)**, oxamic acid **(11)**, benzophenone hydrazine **(12)**, 2-aminoadipic acid **(13)**, D-alanyl-D-alanine **(14)**, *p*-bromophenylalanine **(15)**, nicotinic hydrazide **(16)**, 4-hydroxybenzhydrazide **(17)**, benzohydrazide **(18)**, isonicotinic hydrazide **(19)**, and phenylhydrazine **(20)**, together with monolaurin **(21)** and tetrodotoxin **(22)**. All test sets were run for molecular modelling evaluation based on ligand-based approach to analyse their potential as M^Pro^ inhibitors of SARS-CoV-2 inhibitors.

**Table 2:**
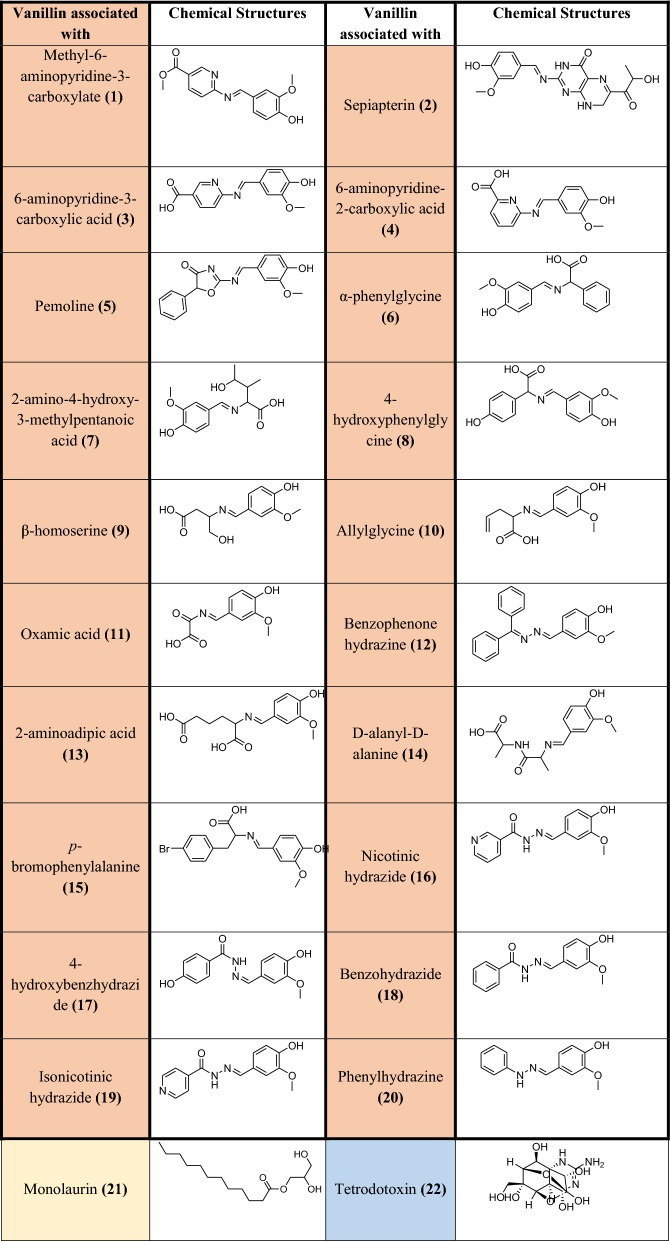
Set of vanillin derivatives

#### Structure-based pharmacophore modelling

Three structures which are crystal structures of SARS-CoV-2 main protease in complex with Z54571979, PCM-0102287 and, coded as 5RE6, 5REX and 5RFZ, are retrieved from Protein Data Bank (Table 3).

**Table 3:**
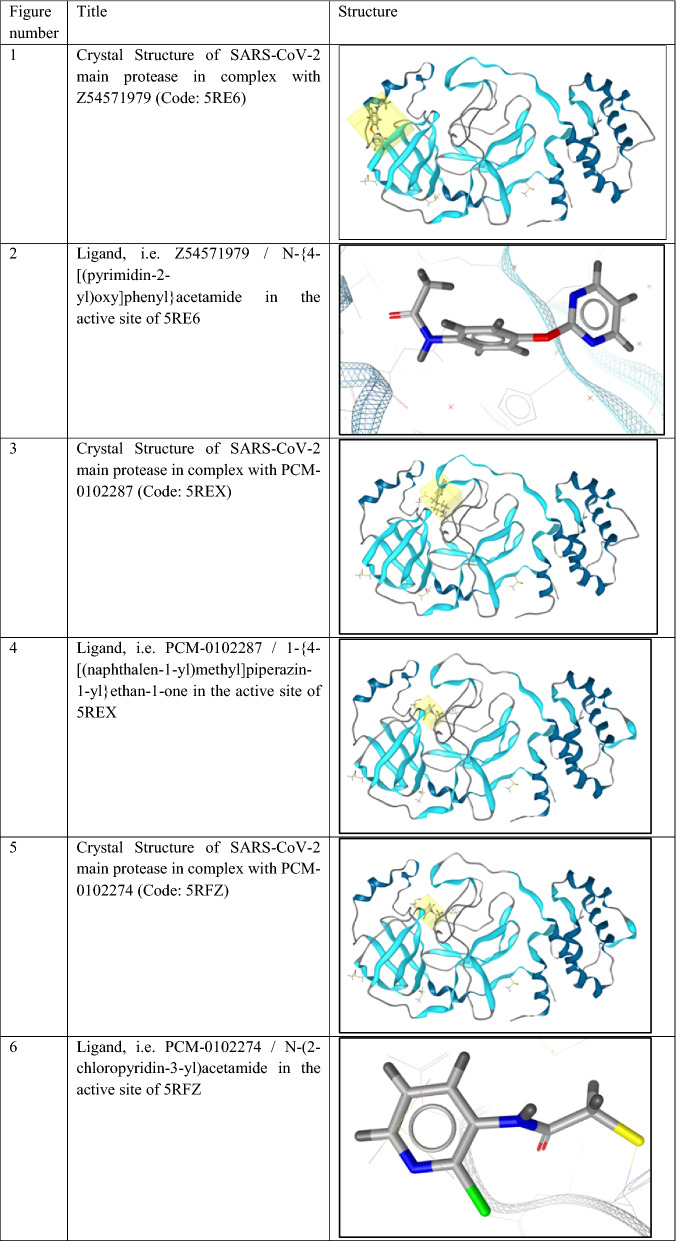
Crystal structures of SARS-CoV-2 main protease in complex with Z54571979, PCM-0102287 and, coded as 5RE6, 5REX and 5RFZ

The pharmacophore models of the ligands in the active site were generated. The pharmacophore models generated and the ligands were copied into the alignment perspective. The vanillin derivative **(1)** was imported into 5RE6 active site (SDF file format), and subjected to the alignment perspective. The overlapping pharmacophore features were generated, merged and interpolated. Upon reimporting to the structure-based perspective, the alignment score and interactions between the vanillin derivative and active sites of the protein were analysed. The steps were repeated for the entire vanillin derivatives.

#### Favipiravir, chloroquine and hydroxychloroquine

Favipiravir, chloroquine and hydroxychloroquine were subjected to structure-based pharmacophore modelling approach by docking into the active sites of 5REX and 5RFZ. The alignment scores and interactions of the compounds in the active sites were further analysed.

### Results and discussion

#### Ligand-based pharmacophore modelling approach

The pharmacophore model generated from the training sets are composed of four common features, which are **two HBA** and **two HBD**. The results of this observation are reported in Table S1 and Table S2. All vanillin derivatives and monolaurin exhibited 3–4 common pharmacophore features, being potentially active compounds against SARS-CoV-2. Based on the alignment score of the vanillin derivatives, **(2)** exhibited the highest score (47.04), followed by **(9)** (45.39). The rest of the vanillin derivatives show alignment score of about 38. In all the vanillin derivatives, the nitrogen in the C=N bond is responsible for one of the HBA feature, whereas the hydroxyl group is responsible for the HBA and HBD features aligned in the pharmacophore model generated. Monolaurin displayed four common pharmacophore features, with C=O aligned with the HBA feature of the model, and hydroxyl groups aligned with the HBA and two HBD features, with high alignment score (46.18). Similarly, tetrodotoxin displayed an alignment score of 43.39, with four common pharmacophore features aligned at the hydroxyl groups as well, with four common pharmacophore features as well, showing that both monolaurin and tetrodotoxin are potential M^Pro^ inhibitors of SARS-CoV-2. All vanillin derivatives were subjected to structure-based approach for further affirmation.

In structure based pharmacophore modelling, the evaluations are based on the pharmacophore features generated by the ligand in the active sites of the retrieved protein, as well as the alignment scores of the test sets with the pharmacophore model of the particular ligand generated. Upon generation of pharmacophore model from the ligand, the model is imported to the alignment perspective where the test sets will be aligned with the model. Such alignment is to elucidate matched or shared features, where one feature is from the reference element’s feature (from the ligand) and one feature is from the element to be aligned (from the test set). From these alignments, alignment scores are calculated based on their shared features and injection in the active site of the protein.

The ligand–protein complex interaction in the active site of 5RE6 showed three features: two HBA to the active sites HOH671A and HOH741A, and one HY interaction to the active site LEU58A. 12 out of 20 vanillin derivatives, **(1)** to **(12)** exhibit the three common features. The alignment scores of the vanillin derivatives with the ligand are very similar, ranging from 36.41 to 38.33, where vanillin associated with pemoline showed the highest alignment score, (38.33).

The ligand in the active site of 5RE6 contain a pyrimidine ring with two nitrogen possessing the HBA features to the HOH671A and HOH741A active sites, as well as a benzene ring to the LEU58A active site. In close observations to the chemical structures of these vanillin derivatives, most of the vanillin derivatives that contain aminopyridines and oxazole derivatives aligned well with the pyrimidine ring in the ligand and exhibited the HBA features to HOH671A and HOH741A, whereas the benzene rings exhibited the HY feature to LEU58A. Therefore, in general, vanillin derivatives with aminopyridines, benzene rings and oxazole derivatives showed better results in both ligand and structure-based pharmacophore modelling. Since these 12 vanillin derivatives are able to align well with the ligand and showed good common pharmacophore features, they are proven in silico as potential inhibitors of the M^Pro^ of SARS-CoV-2 in the active site.

#### Structure-based pharmacophore modelling approach

The results of this study are presented in Table S3 and Table S4. The pharmacophore model generated of 5REX in the active site are made up of two HBA to the GLY143A and CYS145A active sites, and two HY interactions to MET149A active site in the ligand–protein complex. The naphthalene ring in the ligand of the active site is responsible for the two HY interactions, whereas the carbonyl group is responsible for the two HBA features. Chloroquine and hydroxychloroquine are able to align well with the ligand, and exhibited all common features as shown in the pharmacophore model but favipiravir was void. In chloroquine, the substituted hexane showed good alignment with the naphthalene ring as HY interaction, and on the other hand, the nitrogen aligned with the carbonyl group and possessed the two HBA features. For the case of hydroxychloroquine, the quinoline ring possess HY interaction to MET149A, and the hydroxyl group is responsible for the two HBA features. Therefore, the results proved their potential antiviral ability as SARS-CoV-2 M^Pro^ inhibitors through in silico approach.

Other than that, favipiravir showed good alignment with the ligand, i.e. the inhibitor, of 5RFZ, and exhibited common features with the pharmacophore model which are composed of three HBA to the GLY143A, CYS145A and and HOH614A active sites, and one HY interaction to LEU27A and THR25A active sites. The chlorine is responsible for the HY interaction, whereas the nitrogen in the ring and oxygen in the carbonyl group are responsible for the three HBA features. In favipiravir, the fluorine aligned well with the chlorine as HY interaction in the complex, whereas the two nitrogens in the ring aligned well with the nitrogen and oxygen in the ligand as HBA features. However, chloroquine and hydroxychloroquine failed to align with the ligand of 5RFZ. Therefore, favipiravir is also another potential compound to inhibit SARS-CoV-2 M^Pro^.

Despite all the research and reported trials, none of them are conclusive enough to confirm the effectivity of chloroquine and hydroxychloroquine as anti-SARS-CoV-2 drug. A placebo-controlled trial reported on using hydroxychloroquine as postexposure prophylaxis showed that there was no significant difference between patients receiving hydroxychloroquine treatment and patients receiving placebo [35]. The research concluded that hydroxychloroquine did not successfully prevent COVID-19 after high-risk or moderate-risk exposure. Also, chloroquine was reported to be used together with other antiviral drugs in treatment of COVID-19, alone itself is inefficient against SARS-CoV-2. Fourteen (14) Italian tourists that were reported positive for COVID-19 in Medanta Hospital have recovered by treatment using lopinavir, azithromycin and chloroquine, but it was reported that chloroquine did not contributed much [36]. More research on chloroquine and hydroxychloroquine as antiviral drugs for SARS-CoV-2 is necessary.

### Conclusion

From the ligand-based pharmacophore modelling approach, it was concluded that the 20 vanillin derivatives, compound **(1)** to **(20)**, exhibited significant antiviral activity regarded as M^Pro^ inhibitors of SARS-CoV-2. Thus, it means that these potential compounds have some capability to fight COVID-19. Furthermore, monolaurin and tetrodotoxin are also a potent active compounds against SARS-CoV-2 according to ligand-based approach. Further result from structure-based pharmacophore modelling approach suggested that vanillin derivatives **(1)** to **(12)** displayed good result as potent COVID-19 antiviral active compounds. Those established marketed drugs such as favipiravir, chloroquine and hydroxychloroquine have proven to possess potent antiviral activity as M^Pro^ inhibitors of SARS-CoV-2 against COVID-19 in silico. Further investigation should be done in order to ensure the safety and lethality of these compounds.

## Limitations

The data incorporated in this note is based on only preliminary investigation generated on computer assisted software. Further investigation based on in-vitro and eventually in vivo studies will confirm the applicability of the adopted methodology and also the efficiency of proposed compounds in this study.

## Supplementary information

**Additional file 1.** Additional tables.

## Data Availability

All data is compiled in the manuscript.

## Abbreviations

SARS-CoV: Severe acute respiratory syndrome coronavirus
HBA: Hydrogen Bond Acceptors
HY: Hydrophobic
CADD: Computer-aided drug design
WHO: World Health Organization

## References

[1] Huang C, Wang Y, Li X, Ren L, Zhao J, Hu Y (2020). Clinical features of patients infected with 2019 novel coronavirus in Wuhan China. Lancet.

[2] Sexton NR, Smith EC, Blanc H, Vignuzzi M, Peersen OB, Denison MR (2016). Homology-based identification of a mutation in the coronavirus RNA-dependent RNA polymerase that confers resistance to multiple mutagens. J Virol.

[3] Tesini BL: Coronaviruses and Acute Respiratory Syndromes (COVID-19, MERS, and SARS). MSD Manuals. 2020. https://www.msdmanuals.com/professional/infectious-diseases/respiratory-viruses/coronaviruses-and-acute-respiratory-syndromes-covid-19,-mers,-and-sars.

[4] Zhu N, Zhang D, Wang W, Li X, Yang B, Song J (2020). A novel coronavirus from patients with pneumonia in China, 2019. New Engl J Med.

[5] World Health Organization: Summary of probable SARS cases with onset of illness from 1 November 2002 to 31 July 2003. 2003. https://www.who.int/csr/sars/country/table2004_04_21/en/..

[6] World Health Organization: Middle East respiratory syndrome coronavirus (MERS-CoV). 2019. https://www.who.int/emergencies/mers-cov/en/..

[7] Guo YR, Cao QD, Hong ZS, Tan YY, Chen SD, Jin HJ (2020). The origin, transmission and clinical therapies on coronavirus disease 2019 (COVID-19) outbreak—an update on the status. Mil Med Res.

[8] Zhang L, Lin D, Sun X, Curth U, Drosten C, Sauerhering L (2020). Crystal structure of SARS-CoV-2 main protease provides a basis for design of improved α-ketoamide inhibitors. Science.

[9] World Health Organization: Statement on the second meeting of the International Health Regulations (2005) Emergency Committee regarding the outbreak of novel coronavirus (2019-nCoV). 2020. https://www.who.int/news-room/detail/30-01-2020-statement-on-the-second-meeting-of-the-international-health-regulations-(2005)-emergency-committee-regarding-the-outbreak-of-novel-coronavirus-(2019-ncov).

[10] World Health Organization: WHO Director-General's opening remarks at the media briefing on COVID-19. 2020. https://www.who.int/dg/speeches/detail/who-director-general-s-opening-remarks-at-the-media-briefing-on-covid-19---11-march-2020.

[11] Bergman SJ, Cennimo DJ, Miller MM, Olsen KM: Treatment of Coronavirus Disease 2019 (COVID-19): Investigational Drugs and Other Therapies. Medscape. 2020. https://emedicine.medscape.com/article/2500116-overview.

[12] Yin Y, Wunderink RG (2017). MERS, SARS and other coronaviruses as causes of pneumonia. Respirology.

[13] Chen Y, Liu Q, Guo D (2020). Emerging coronaviruses: Genome structure, replication, and pathogenesis. J Med Virol.

[14] Walls AC, Park Y, Tortorici MA, Wall A, McGuire AT, Veesler D (2020). Structure, function, and antigenicity of the SARS-CoV-2 spike glycoprotein. Cell.

[15] Schoeman D, Fielding BC (2019). Coronavirus envelope protein: current knowledge. Virol J..

[16] Xu X, Chen P, Wang J, Feng J, Zhou H, Li X (2020). … Hao P: Evolution of the novel coronavirus from the ongoing Wuhan outbreak and modeling of its spike protein for risk of human transmission. Sci China Life Sci.

[17] Cheng VC, Lau SK, Woo PC, Kwok YY (2007). Severe acute respiratory syndrome coronavirus as an agent of emerging and reemerging infection. Clin Microbiol Rev.

[18] Xue X, Yu H, Yang H, Xue F, Wu Z, Shen W (2008). Structures of two coronavirus main proteases: implications for substrate binding and antiviral drug design. J Virol.

[19] Asaruddin MR: Modelling and syntheses of vanillin derivatives targeting influenza virus neuraminidase. Malaysian academic library institutional repository. 2016. https://eprints.usm.my/45418/1/MOHD%20RAZIP%20ASARUDDIN.pdf.

[20] Kim JH, Lee HO, Cho YJ, Kim J, Chun J, Choi J (2014). A vanillin derivative causes mitochondrial dysfunction and triggers oxidative stress in *Cryptococcus neoformans*. PLoS ONE..

[21] The Science Times: ‘Favilavir’: First Approved Drug to Possibly Treat Coronavirus. 2020. https://www.sciencetimes.com/articles/25053/20200317/favilavir-first-approve-drug-treat-coronavirus.htm.

[22] Hospimedica: Fujifilm’s Antiviral Becomes First Approved Drug to Treat Coronavirus in China. 2020. https://www.hospimedica.com/coronavirus/articles/294781247/fujifilms-antiviral-becomes-first-approved-drug-to-treat-coronavirus-in-china.html.

[23] Goldhill DH, Te Velthuis AJW, Fletcher RA, Langat P, Zambon M, Lackenby A, Barclay WS (2018). The mechanism of resistance to favipiravir in influenza. Proc Natl Acad Sci USA.

[24] Romanelli F, Smith K, Hoven A (2004). Chloroquine and hydroxychloroquine as inhibitors of human immunodeficiency virus (HIV-1) activity. Curr Pharm Design.

[25] The Centre for Evidence-Based Medicine: Chloroquine and hydroxychloroquine: Current evidence for their effectiveness in treating COVID-19. 2020. https://www.cebm.net/covid-19/chloroquine-and-hydroxychloroquine-current-evidence-for-their-effectiveness-in-treating-covid-19/.

[26] Wang M, Cao R, Zhang L, Yang X, Liu J, Xu M (2020). Remdesivir and chloroquine effectively inhibit the recently emerged novel coronavirus (2019-nCoV) in vitro. Cell Res.

[27] Xia J, Liu X, Chen H, Shang Y, Zhu H, Chen G, et al. Efficacy of Chloroquine and Lopinavir/Ritonavir in mild/general COVID-2019: a prospective, open-label, multicenter randomized controlled clinical study. 2020. Doi: 10.21203/rs.3.rs-16392/v1 10.1186/s13063-020-04478-w PMC7341476 32641091

[28] Vincent MJ, Bergeron E, Benjannet S, Erickson BR, Rollin PE, Ksiazek TG (2005). Chloroquine is a potent inhibitor of SARS coronavirus infection and spread. Virol J.

[29] Liu J, Cao R, Xu M, Wang X, Zhang H, Hu H (2020). Hydroxychloroquine, a less toxic derivative of chloroquine, is effective in inhibiting SARS-CoV-2 infection in vitro. Cell Discov..

[30] Baltić B, Starčević M, Dordević J, Mrdović B, Marković R (2017). Importance of medium chain fatty acids in animal nutrition. IOP Conf Ser Earth Environ Sci.

[31] Dayrit FM, Newport MT: The Potential of Coconut Oil and its Derivatives as Effective and Safe Antiviral Agents Against the Novel Coronavirus (nCoV-2019). Integrated Chemists of Philippines. 2020. https://www.icp.org.ph/2020/01/the-potential-of-coconut-oil-and-its-derivatives-as-effective-and-safe-antiviral-agents-against-the-novel-coronavirus-ncov-2019/.

[32] Hwang DF, Noguchi T (2007). Tetrodotoxin Poisoning. Adv Food Nutr Res.

[33] Bane V, Lehane M, Dikshit M, O’Riordan A, Furey A (2014). Tetrodotoxin: Chemistry, toxicity, source, distribution and detection. Toxins.

[34] Neil AH, Patrick DS, Bernard L, May OL, Benoit D, David W, Robin L, Anh HN. Tetrodotoxin for moderate to severe cancer pain: a randomized, double blind, parallel design multicenter study. J Pain Sympt Manag. 35: 420–429. Doi: 10.1016/j.jpainsymman.2007.05.011. 10.1016/j.jpainsymman.2007.05.011 18243639

[35] Boulware DR, Pullen MF, Bangdiwala AS, Pastick KA, Lofgren SM, Okafor EC (2020). A randomized trial of hydroxychloroquine as postexposure prophylaxis for Covid-19. N Engl J Med.

[36] Chandra H: Paracetamol, chloroquine & Google translator: How Medanta treated Italians with coronavirus. The Print. 2020. https://theprint.in/health/paracetamol-chloroquine-google-translator-how-medanta-treated-italians-with-coronavirus/387144/.

