# Supplementary material for: Pharmacophore modelling of vanillin derivatives, favipiravir, chloroquine, hydroxychloroquine, monolaurin and tetrodotoxin as MPro inhibitors of severe acute respiratory syndrome coronavirus-2 (SARS-CoV-2)
Source: BMC Res Notes. 2020 Nov 11;13:527. doi: 10.1186/s13104-020-05379-6 (PMC7656897; doi:10.1186/s13104-020-05379-6)
Supplement: Supplementary file 1 — Additional file 1. Additional tables. [file 13104_2020_5379_MOESM1_ESM.docx]

**Table S1: Pharmacophore fit and matching features of test sets in the pharmacophore model**

| **Test Sets** | **M^Pro^ inhibitors of SARS-CoV-2** | |
| --- | --- | --- |
| **Vanillin Derivatives** | **Matching features** | **Pharmacophore fit** |
| **Methyl-6-aminopyridine-3-carboxylate (1)** | 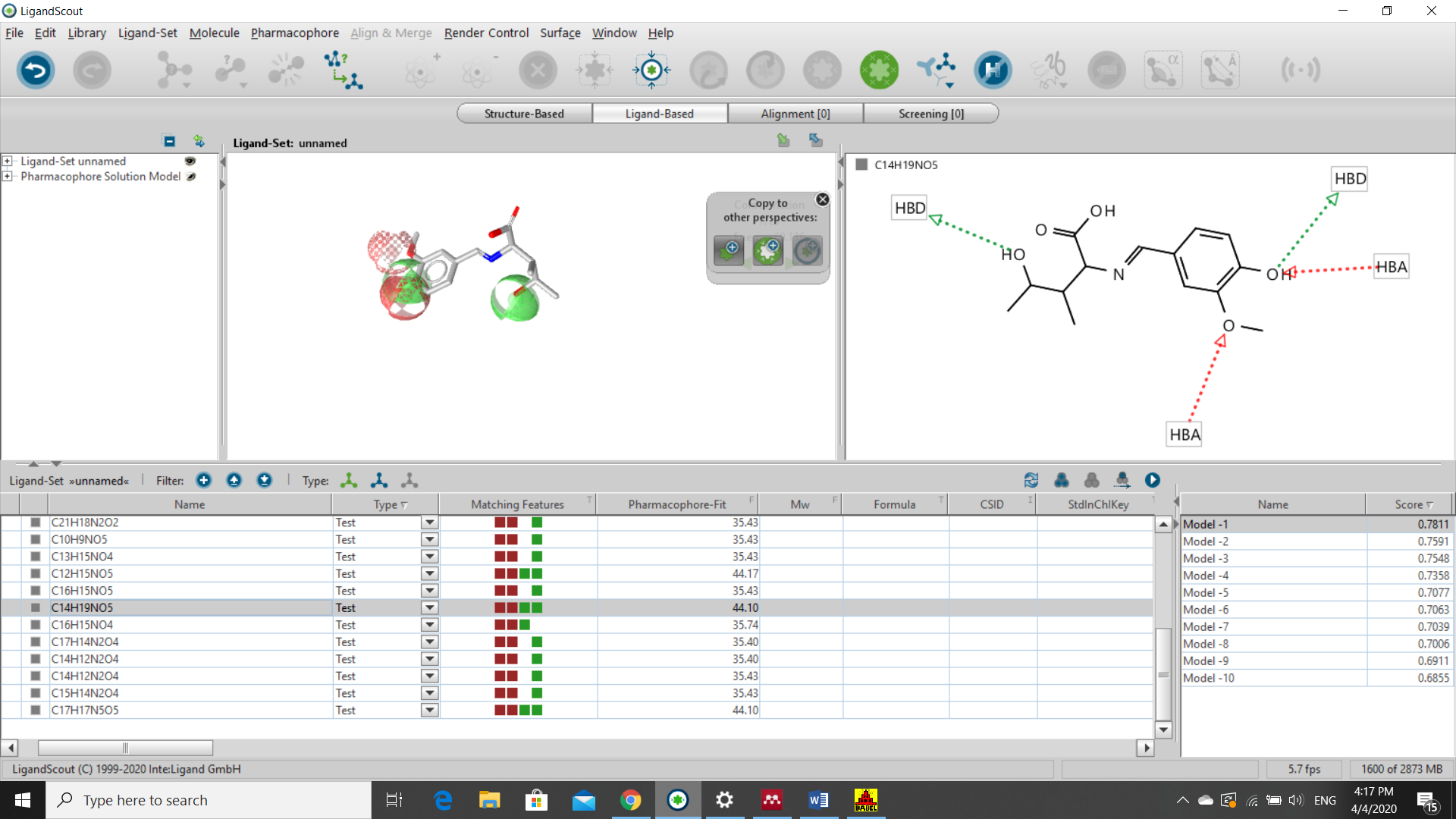 | 38.49 |
| **Sepiapterin (2)** | 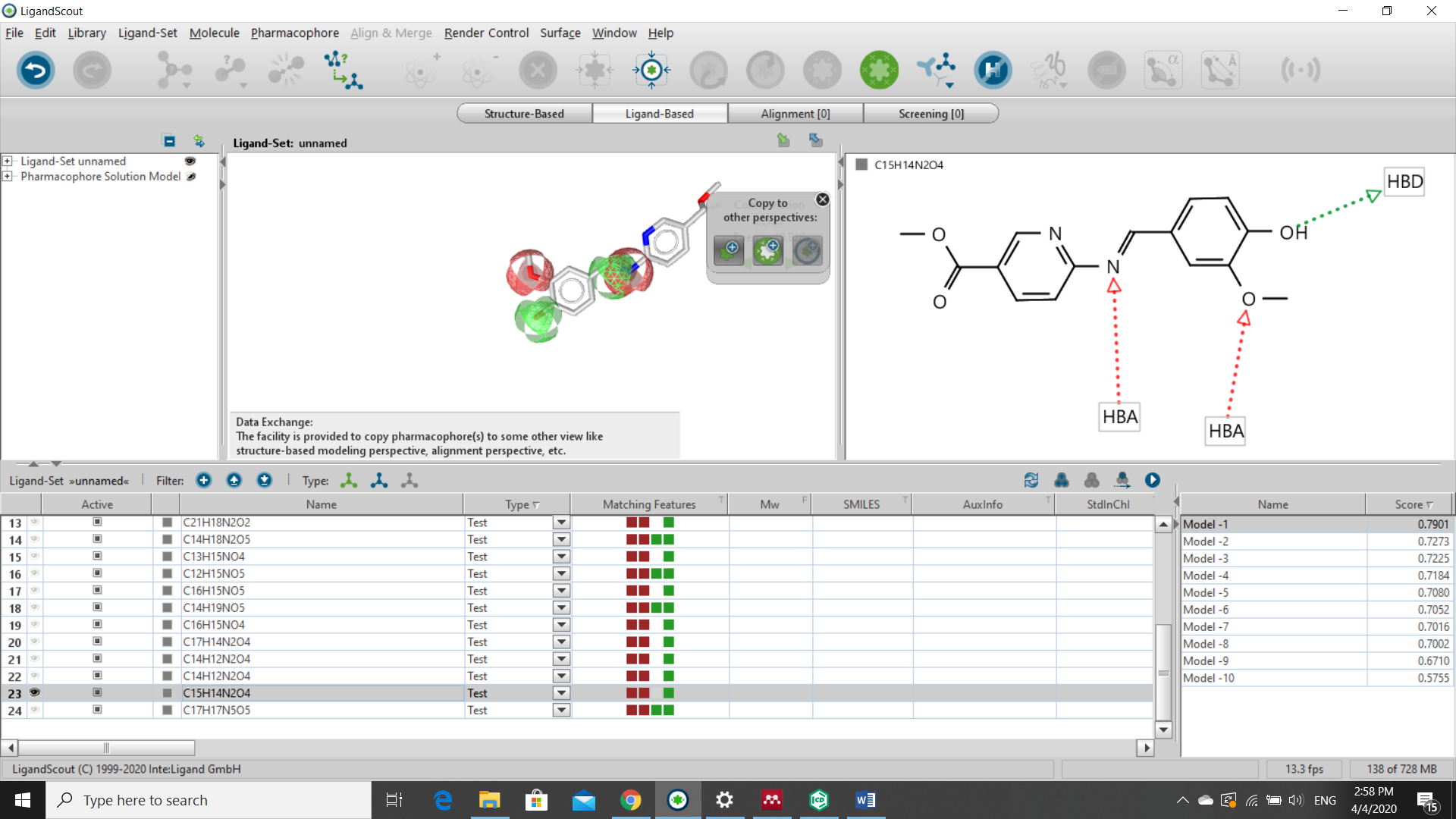 | 47.04 |
| **6-aminopyridine-3-carboxylic acid (3)** | 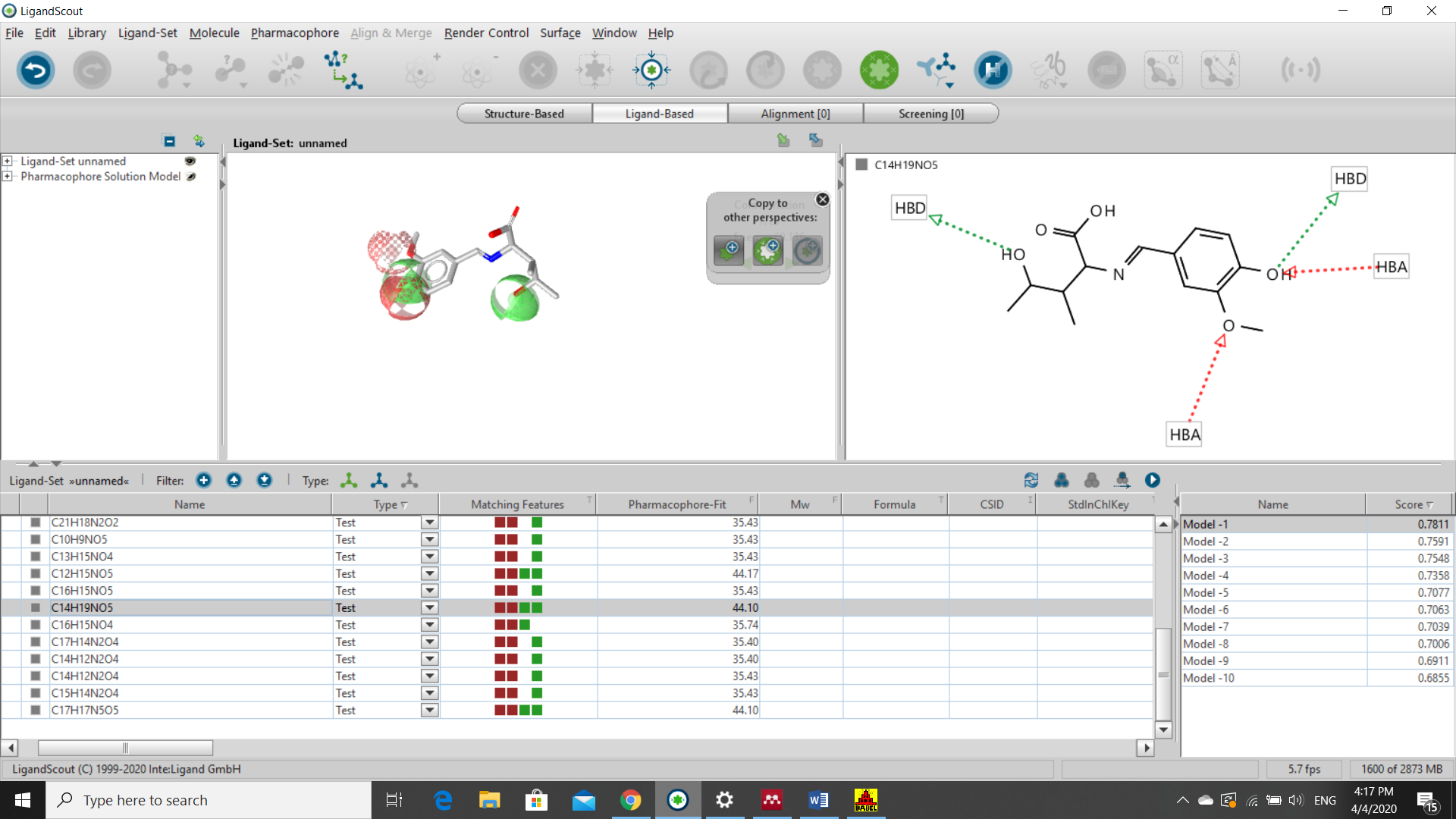 | 38.49 |
| **6-aminopyridine-2-carboxylic acid (4)** | 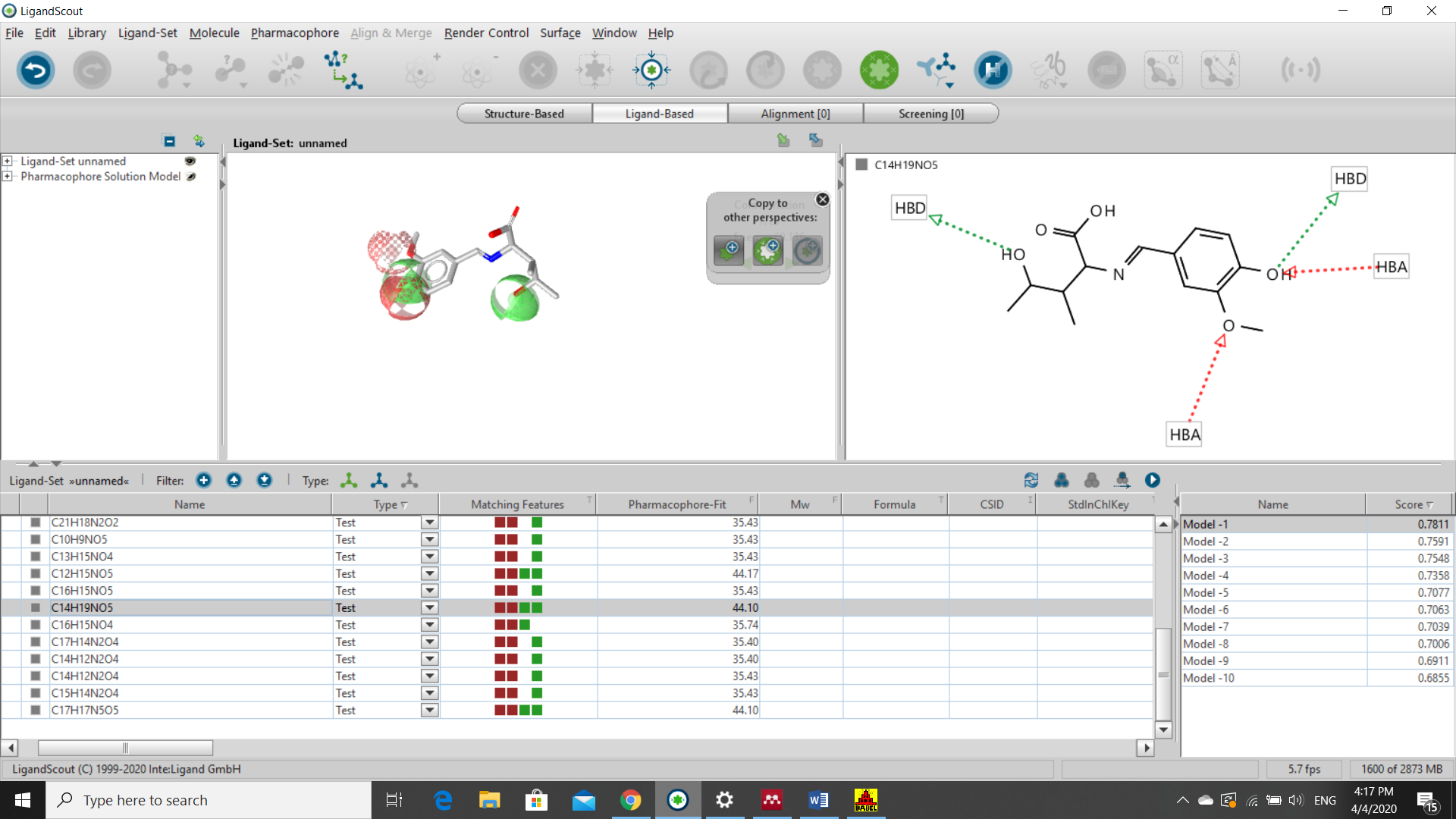 | 38.49 |
| **Pemoline (5)** | 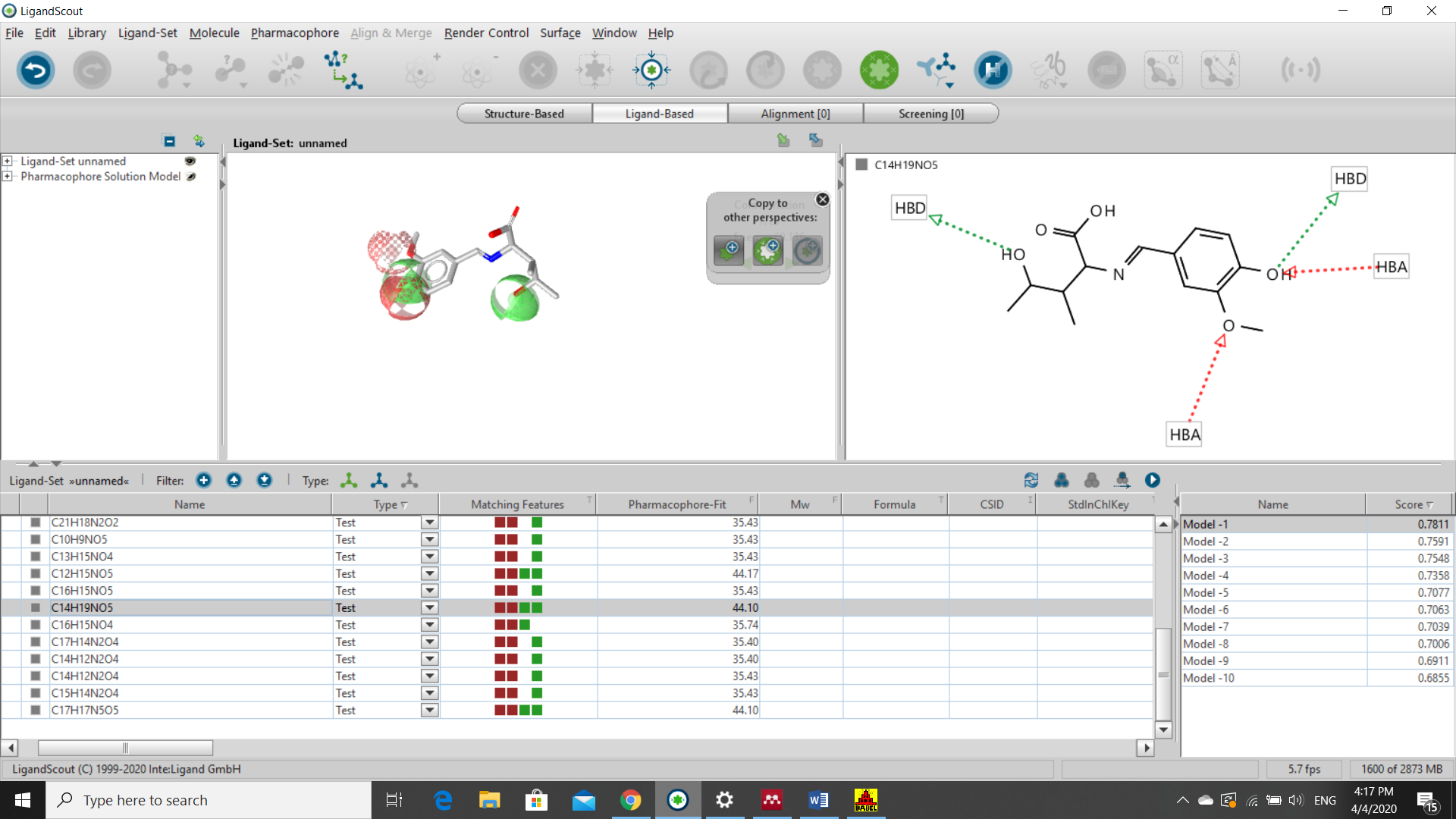 | 38.52 |
| **α-phenylglycine (6)** | 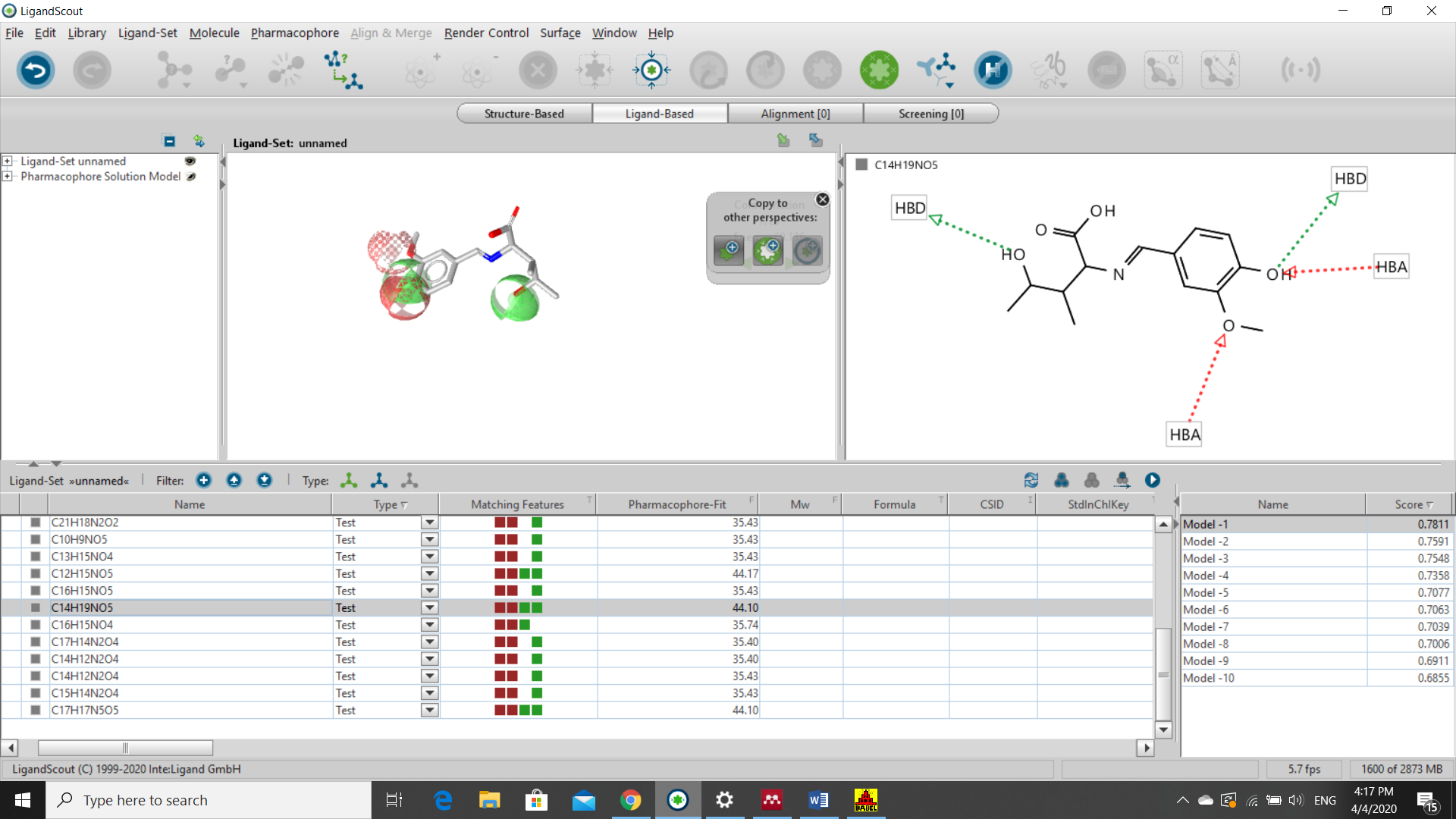 | 38.51 |
| **2-amino-4-hydroxy-3-methylpentanoic acid (7)** | 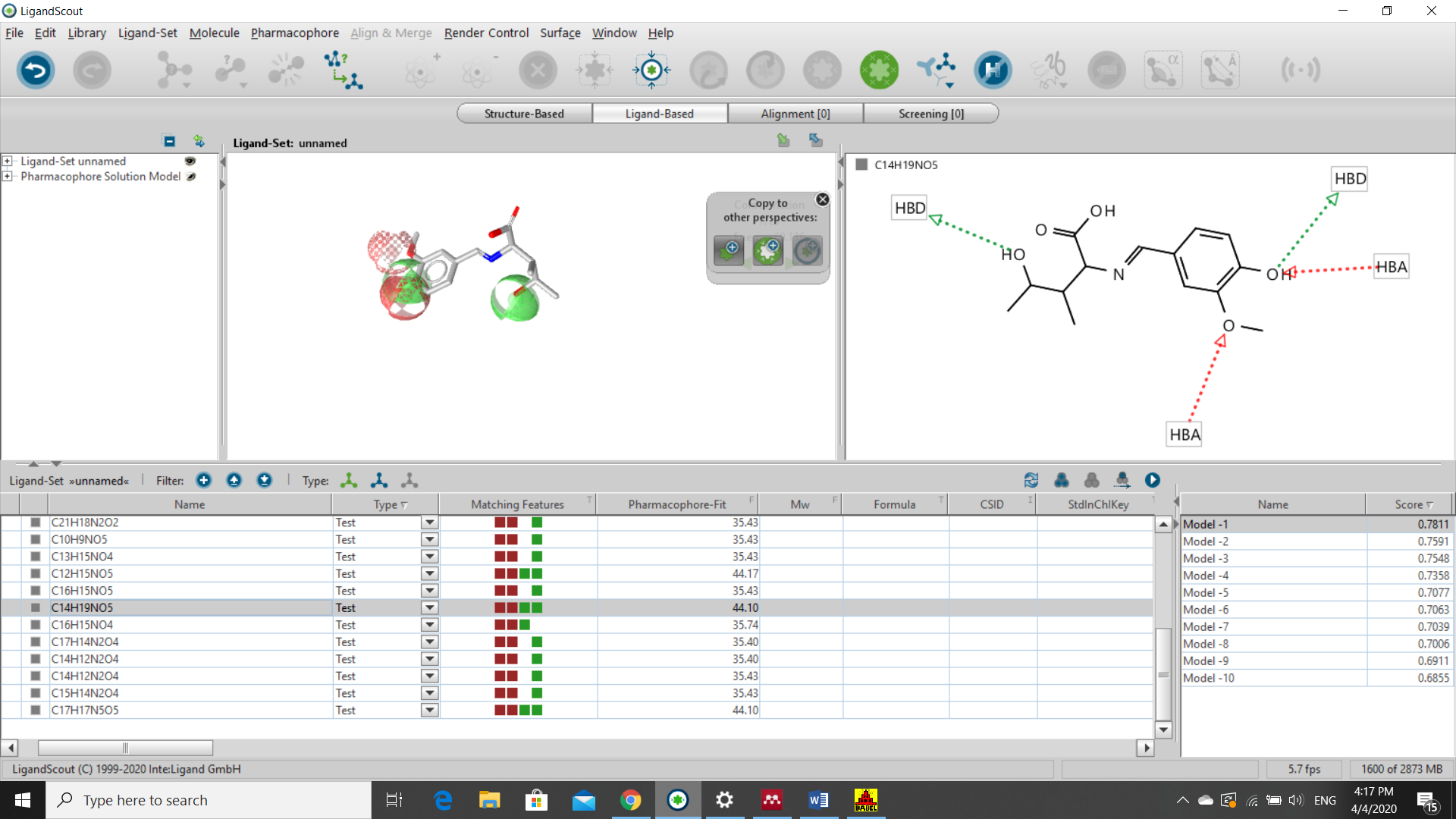 | 38.51 |
| **4-hydroxyphenylglycine (8)** | 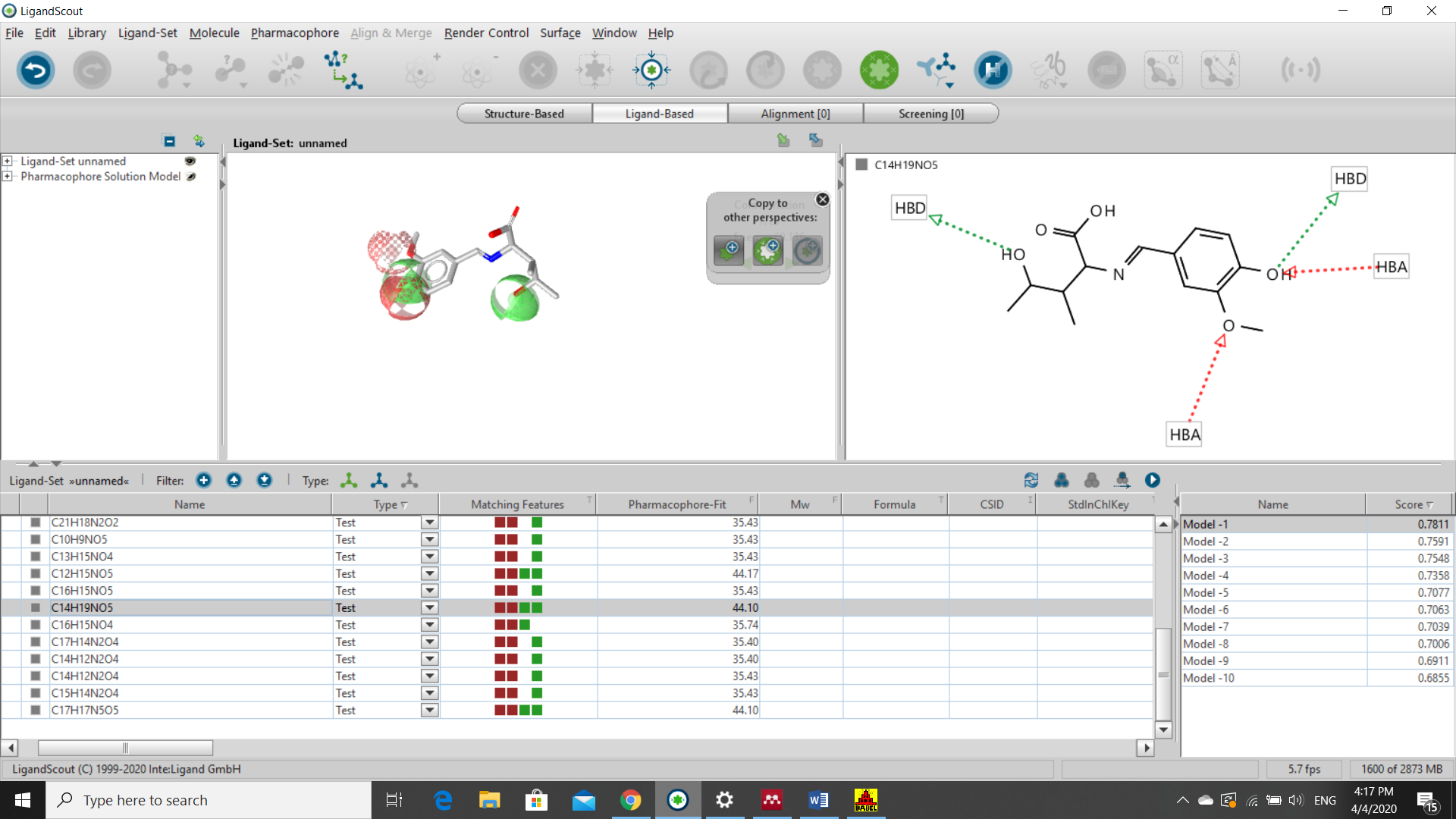 | 38.94 |
| **β-homoserine (9)** | 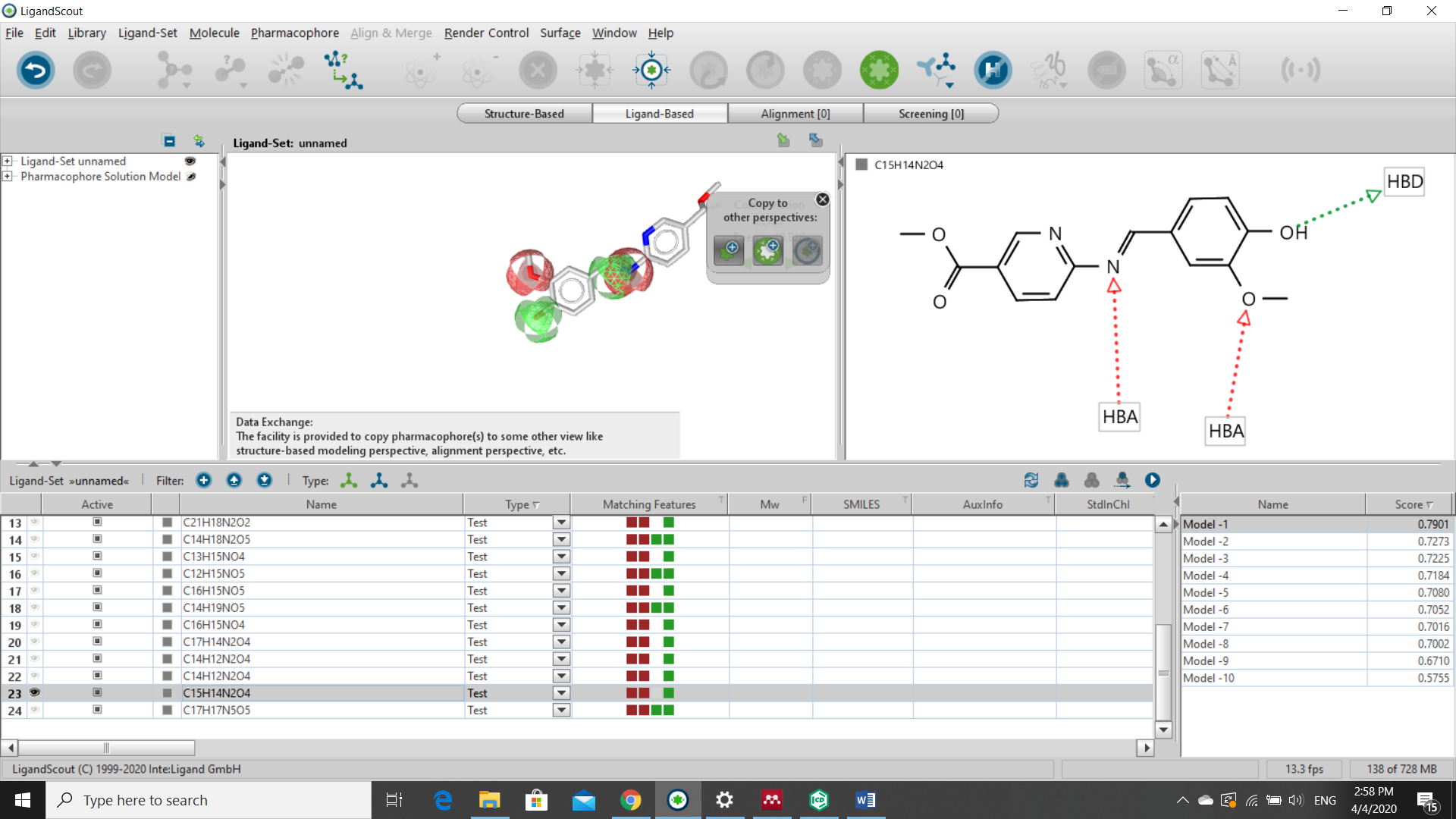 | 45.39 |
| **Allylglycine (10)** | 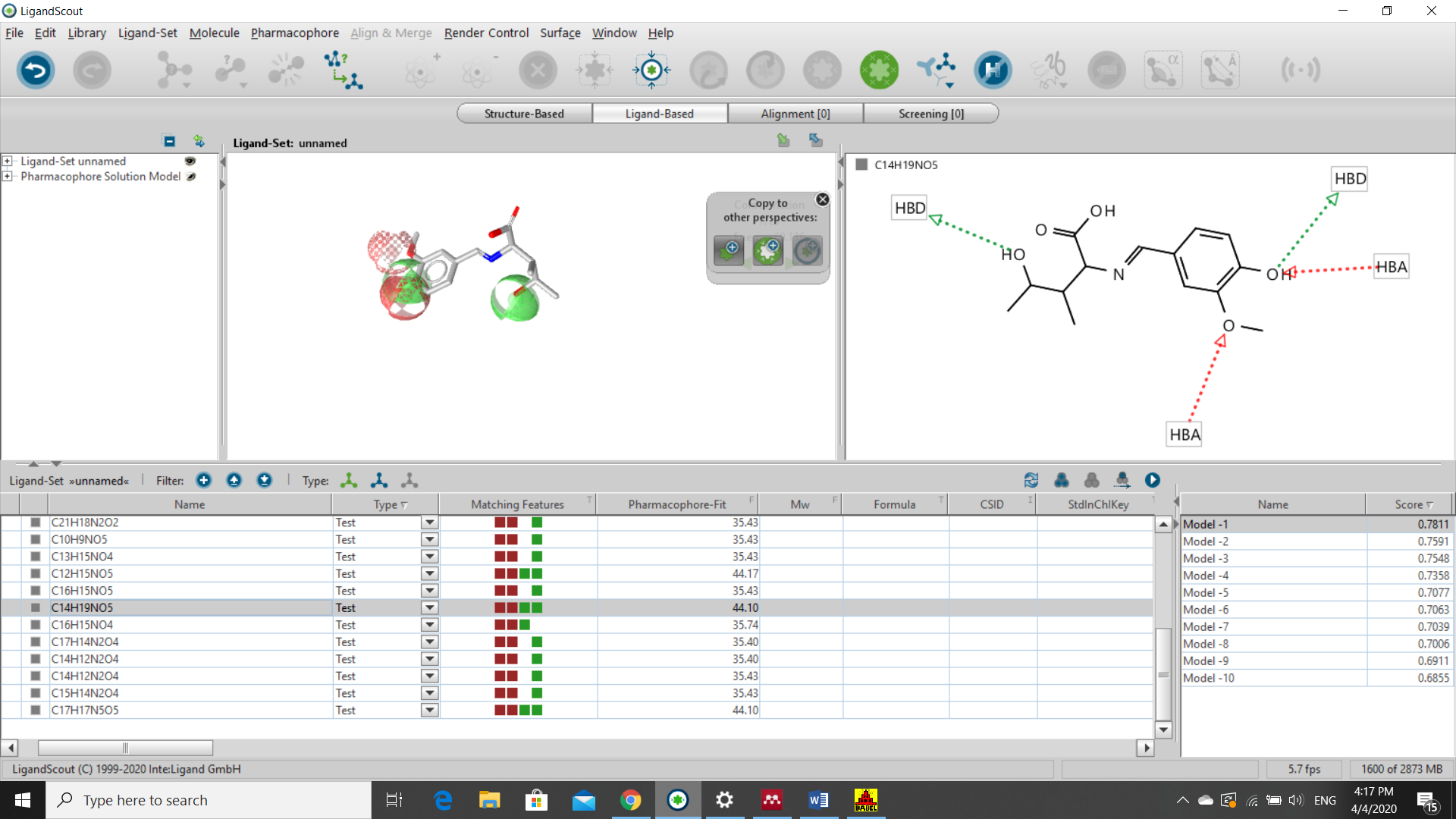 | 38.49 |
| **Oxamic acid (11)** | 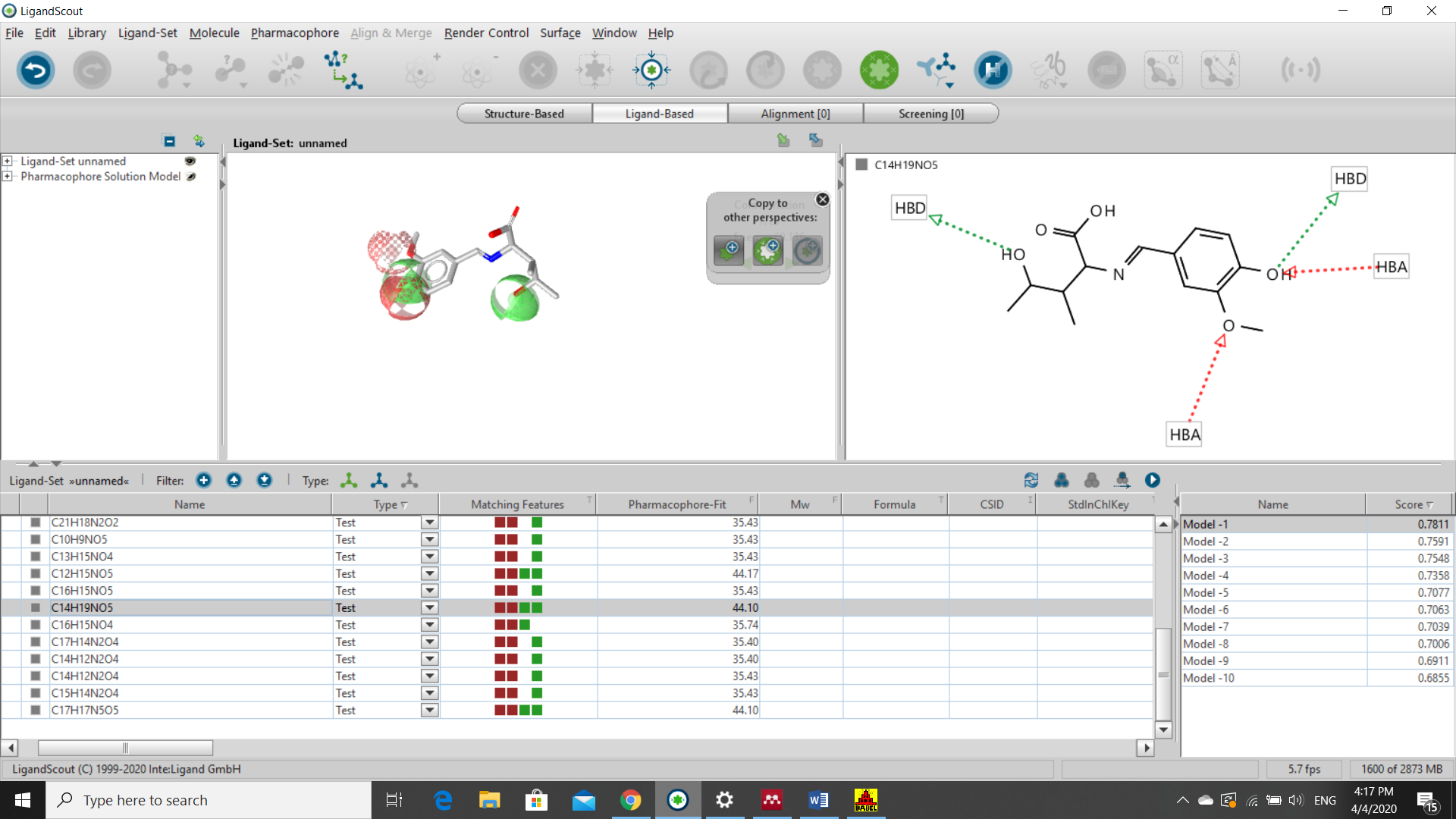 | 38.52 |
| **Benzophenone hydrazine (12)** | 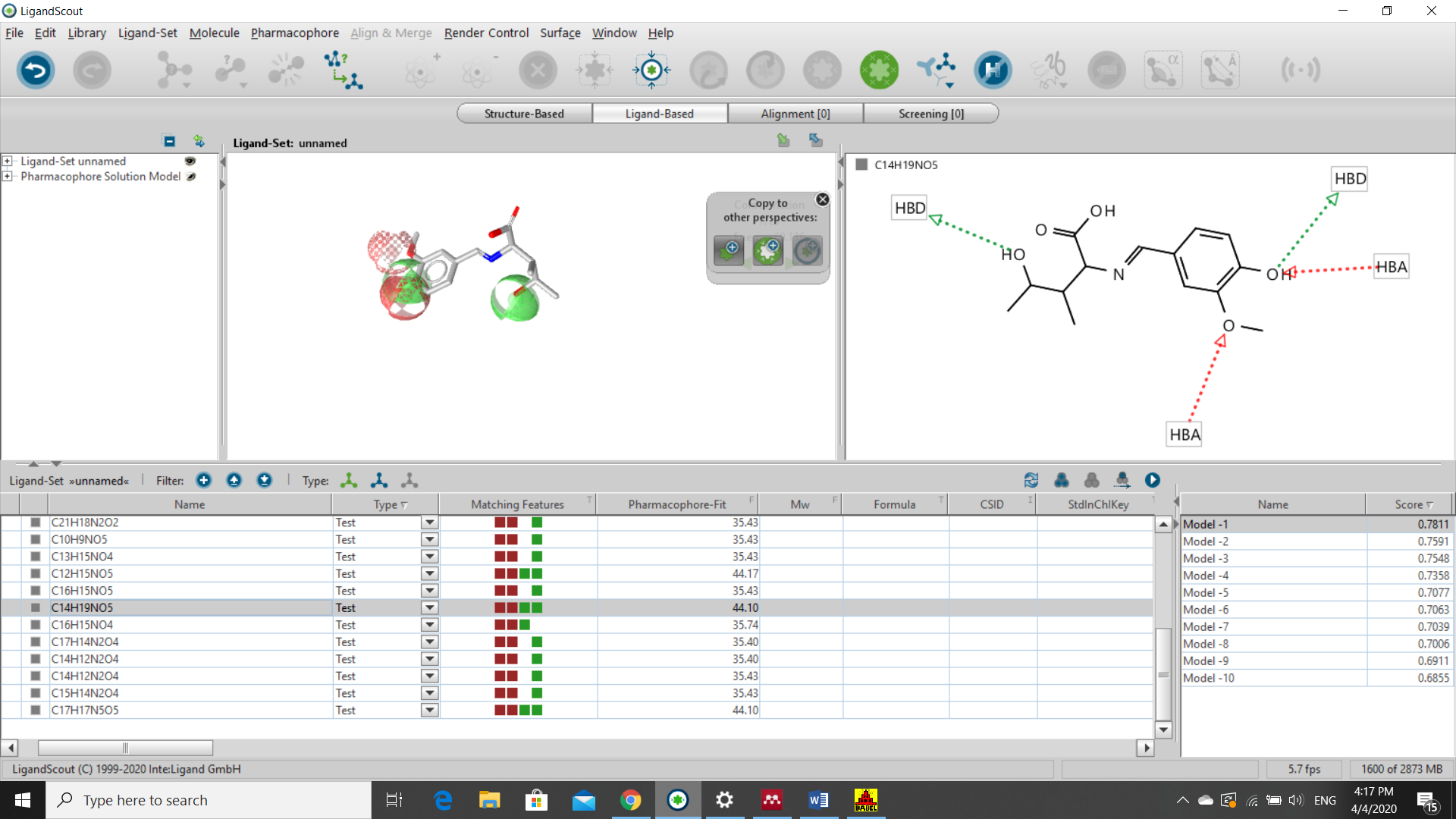 | 38.51 |
| **2-aminoadipic acid (13)** | 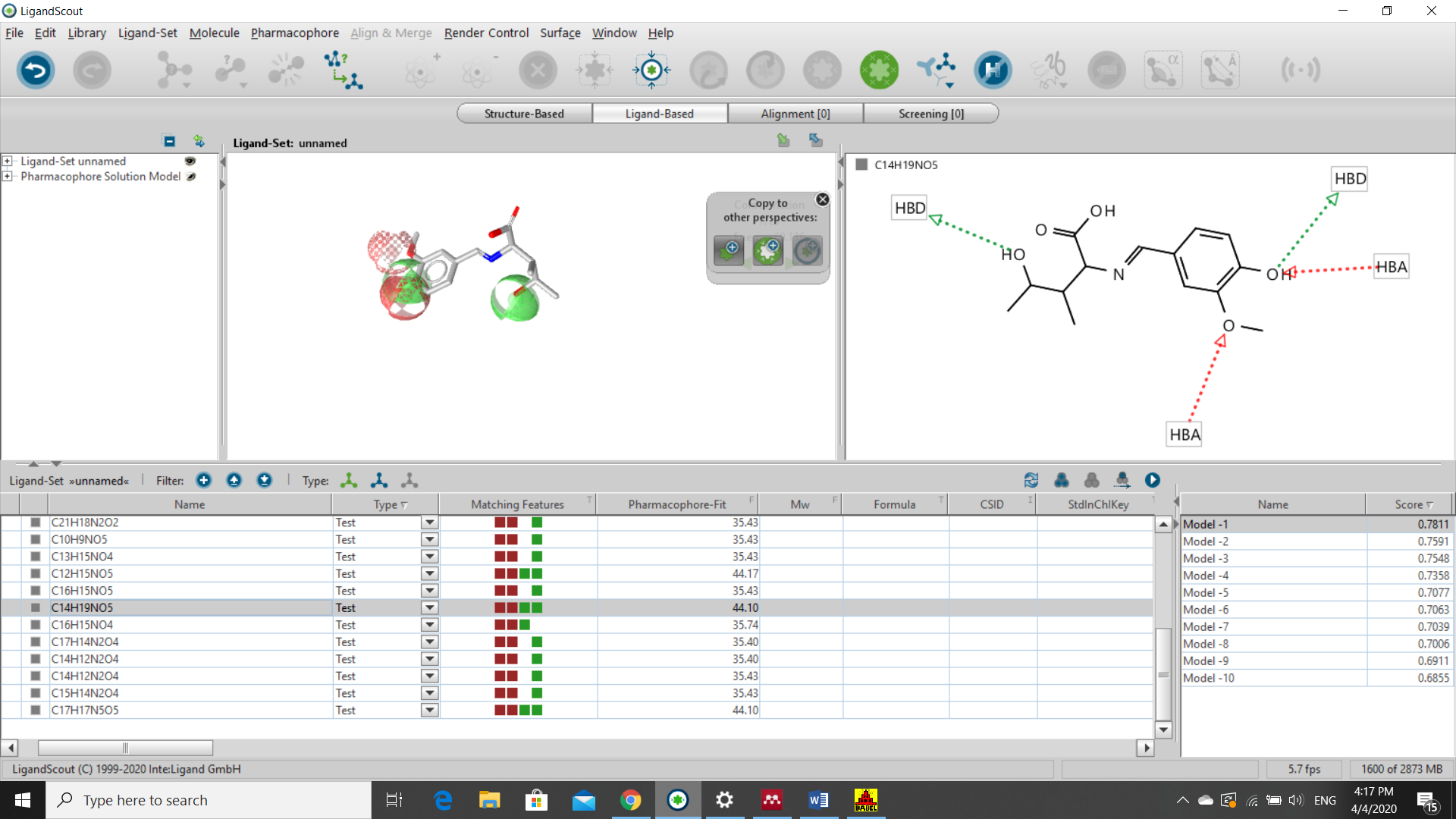 | 38.49 |
| **D-alanyl-D-alanine (14)** | 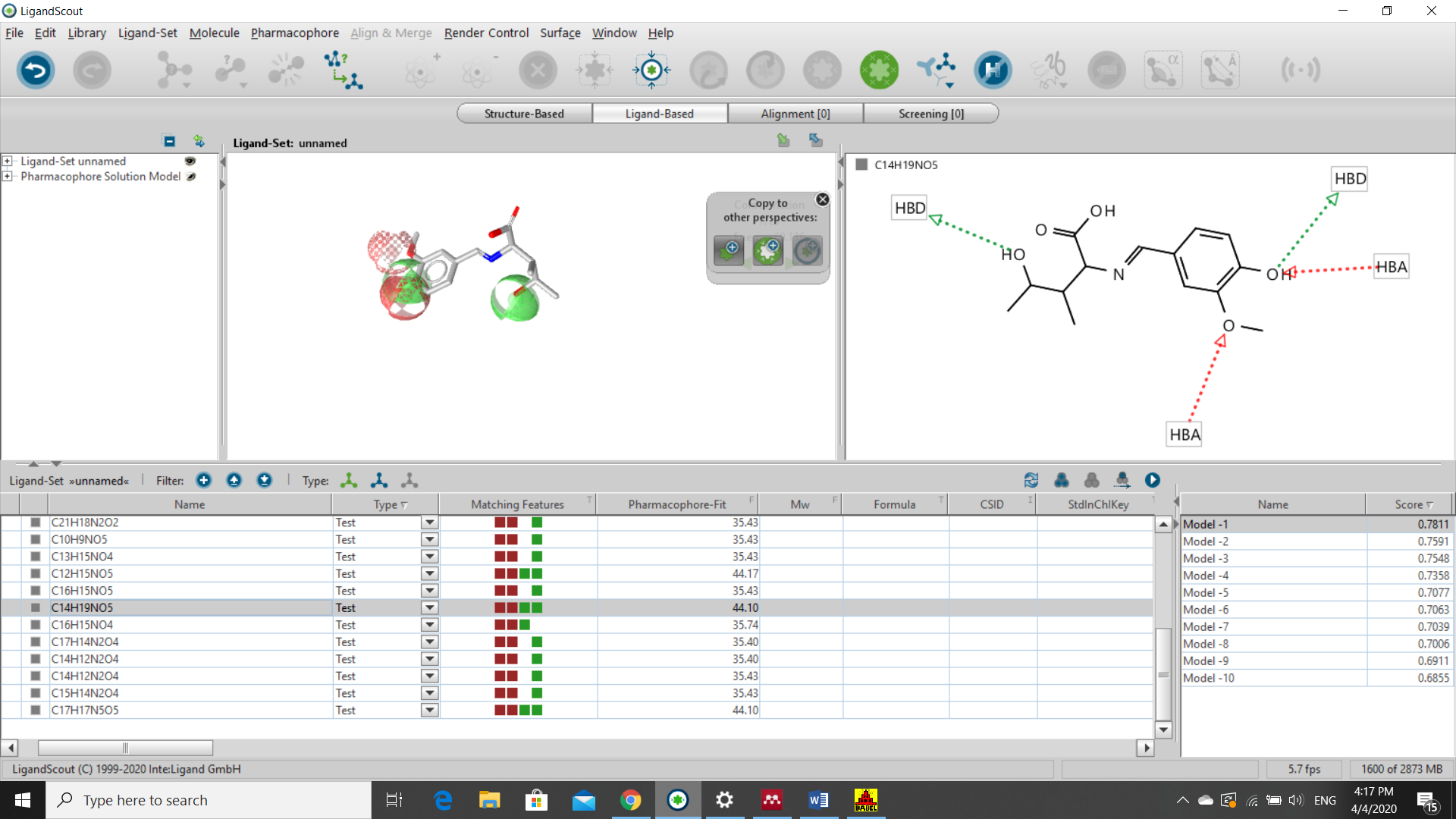 | 38.88 |
| ***p*-bromophenylalanine (15)** | 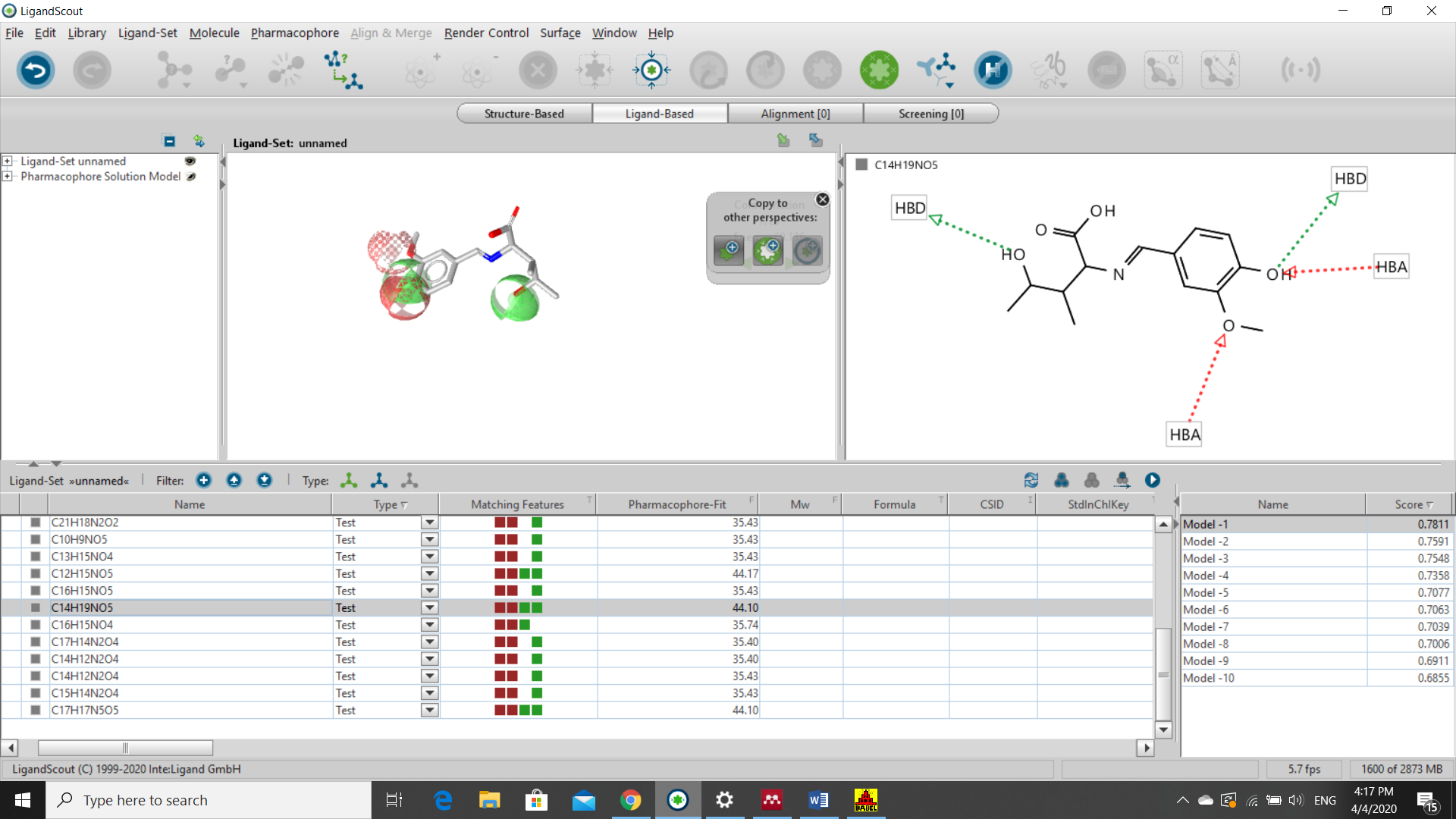 | 38.49 |
| **Nicotinic hydrazide (16)** | 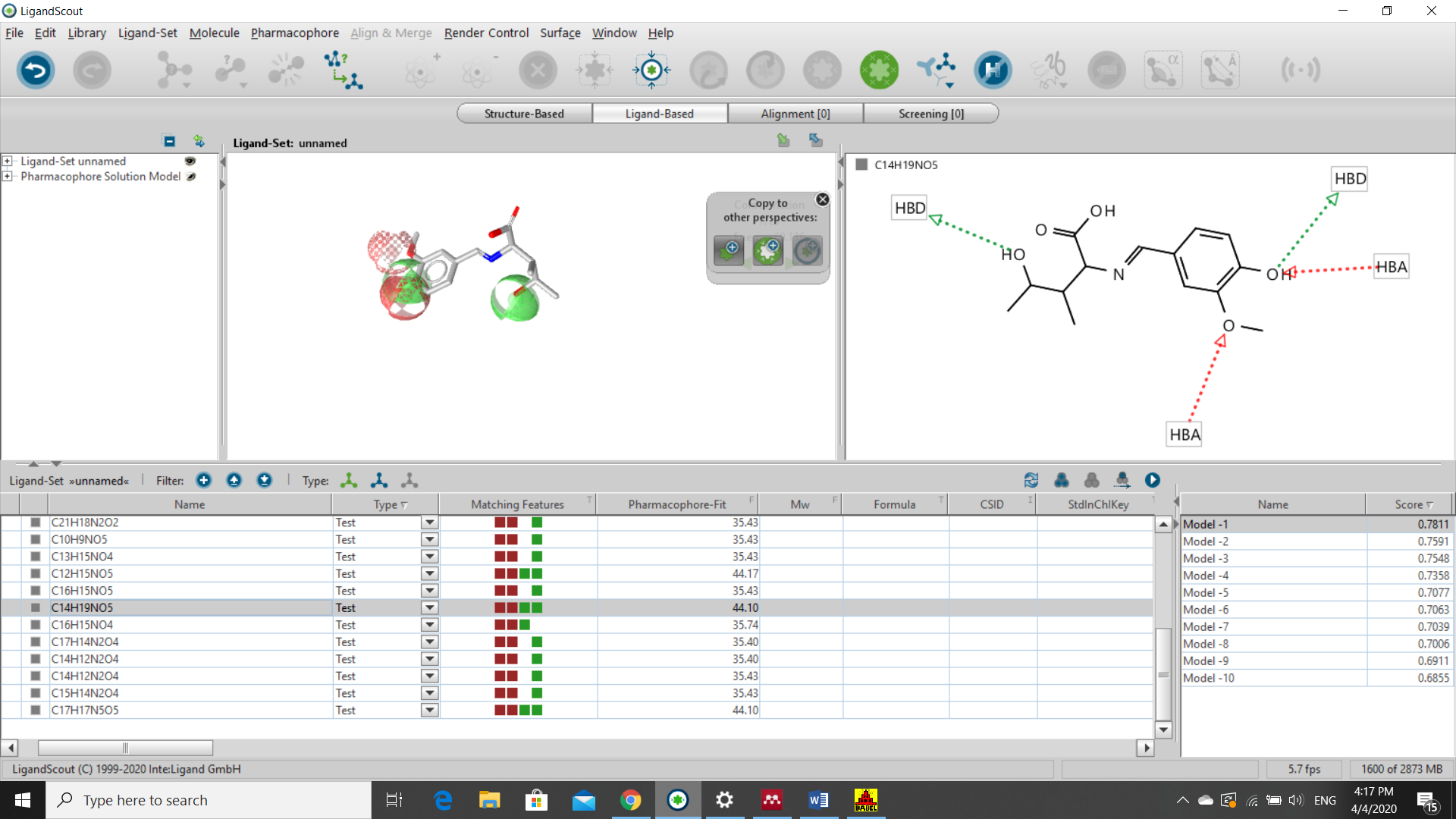 | 38.70 |
| **4-hydroxybenzhydrazide (17)** | 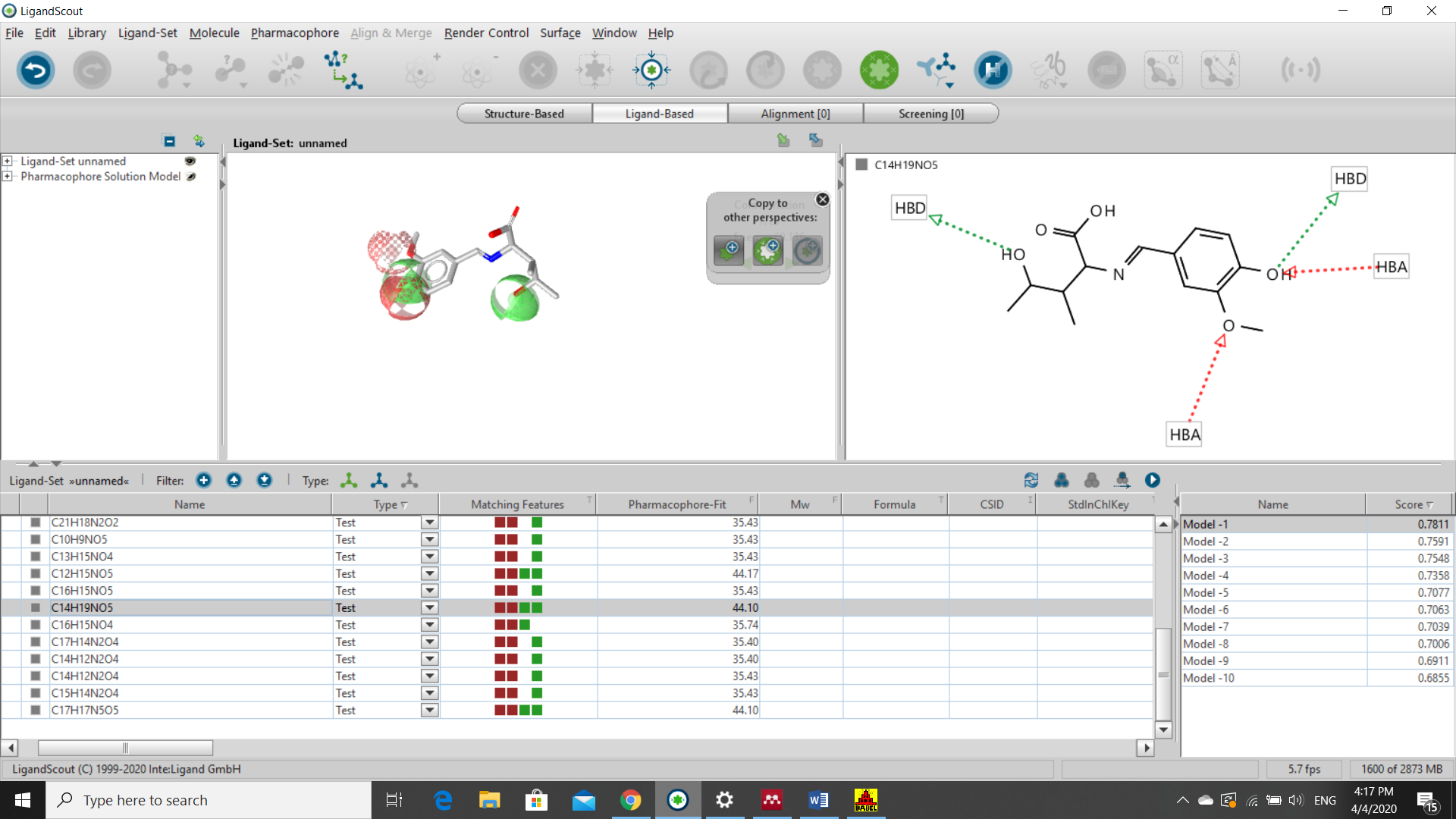 | 38.74 |
| **Benzohydrazide (18)** | 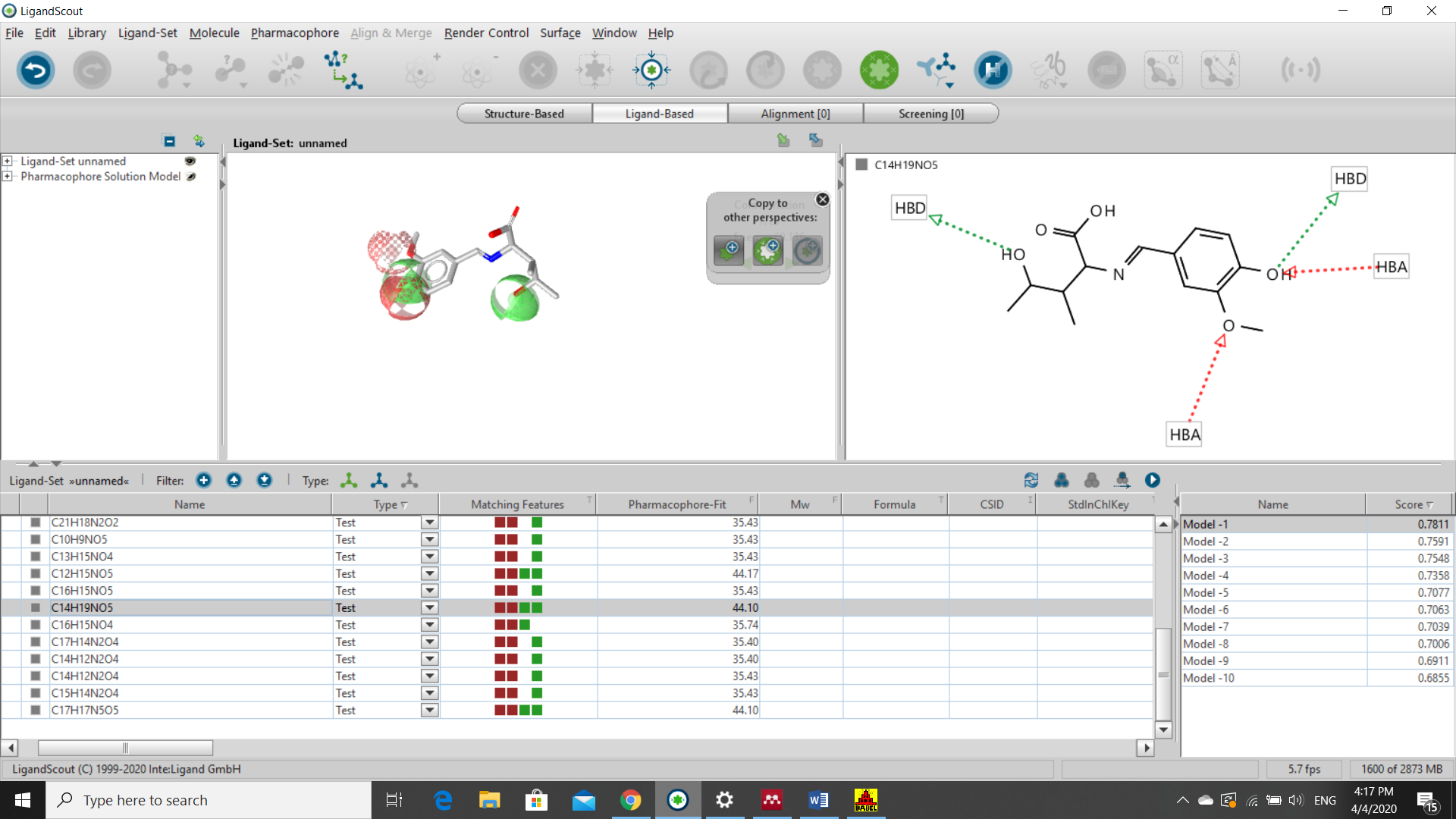 | 38.72 |
| **Isonicotinic hydrazide (19)** | 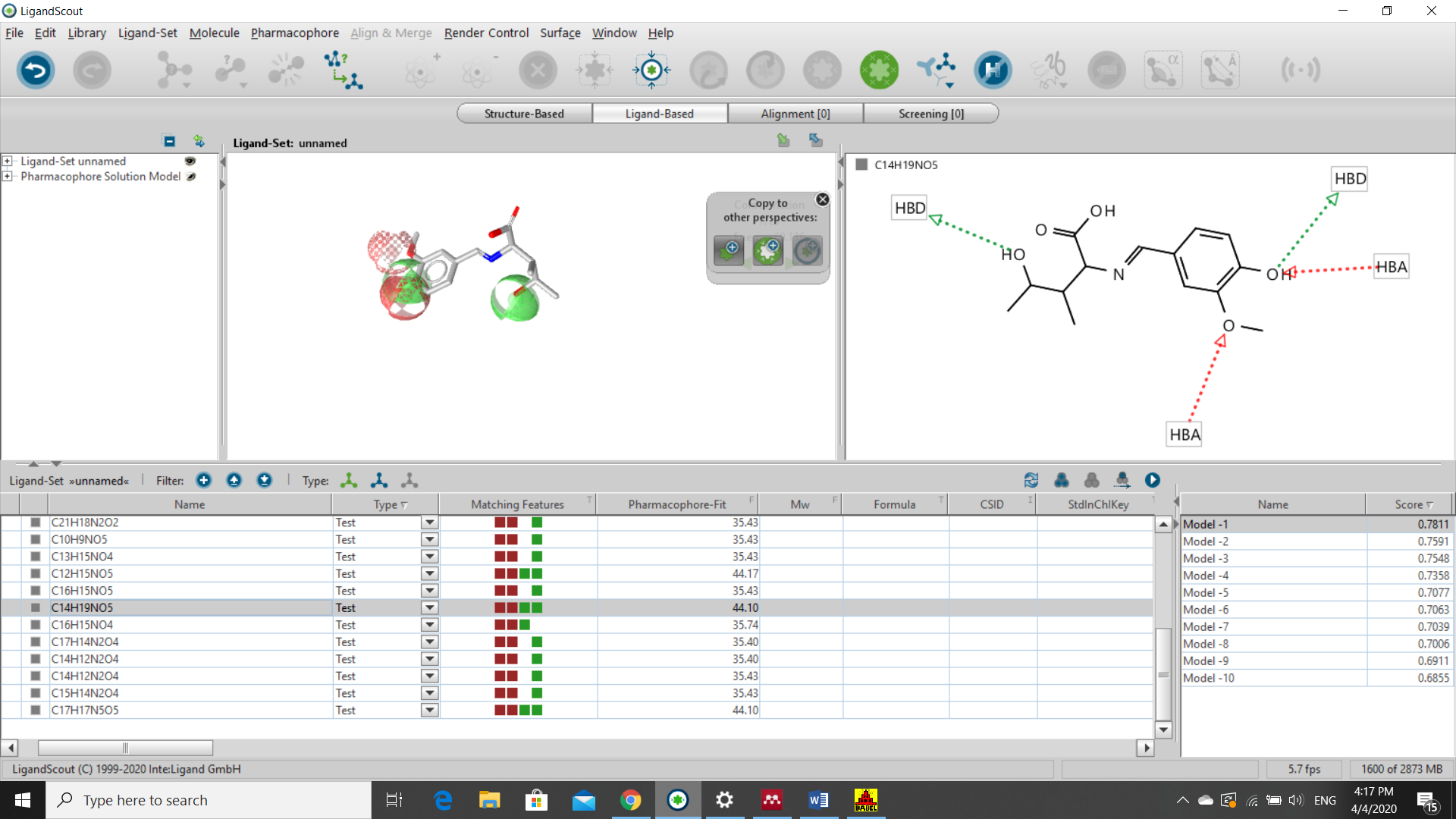 | 38.70 |
| **Phenylhydrazine (20)** | 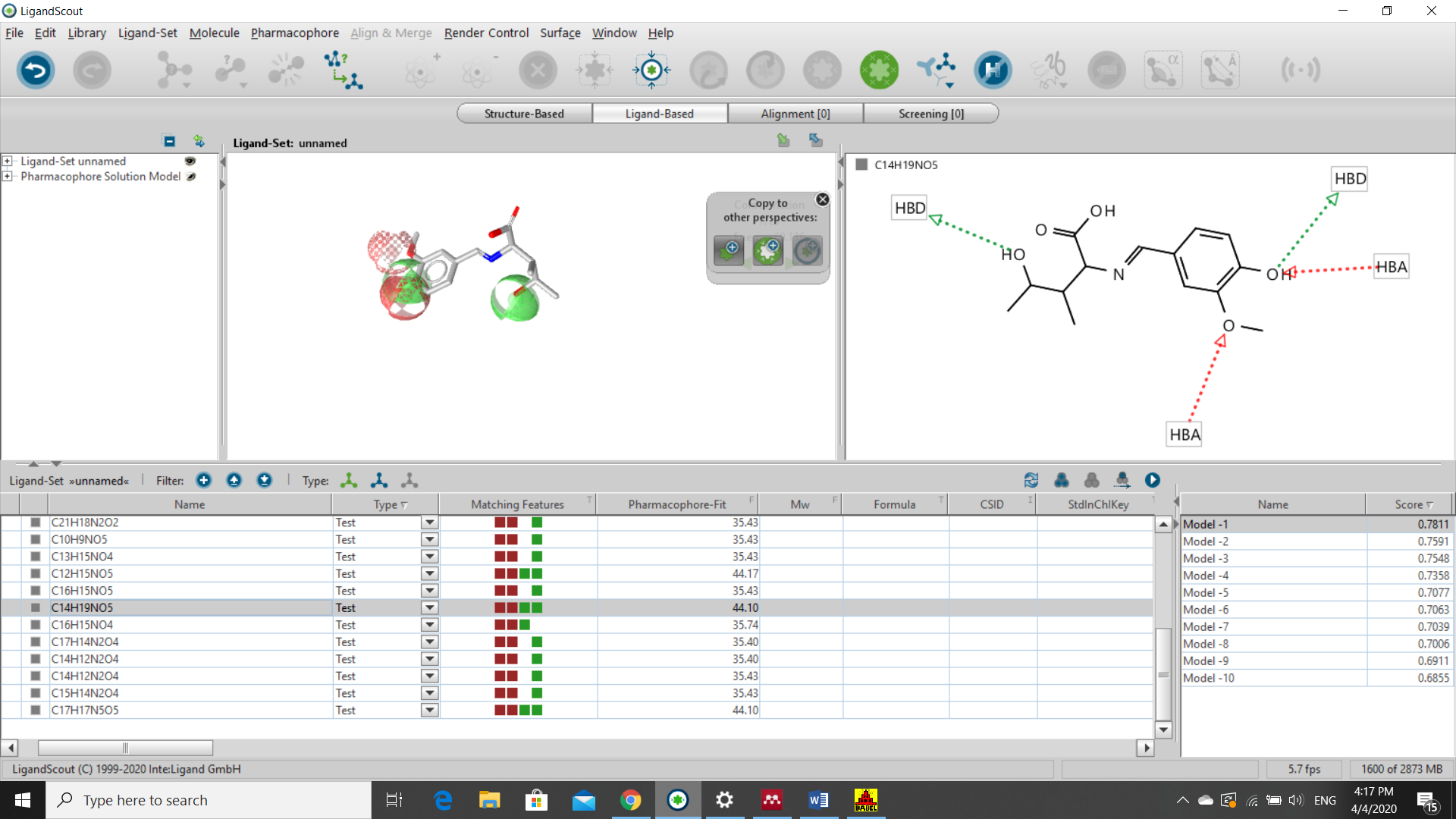 | 38.72 |
| **Monolaurin** | 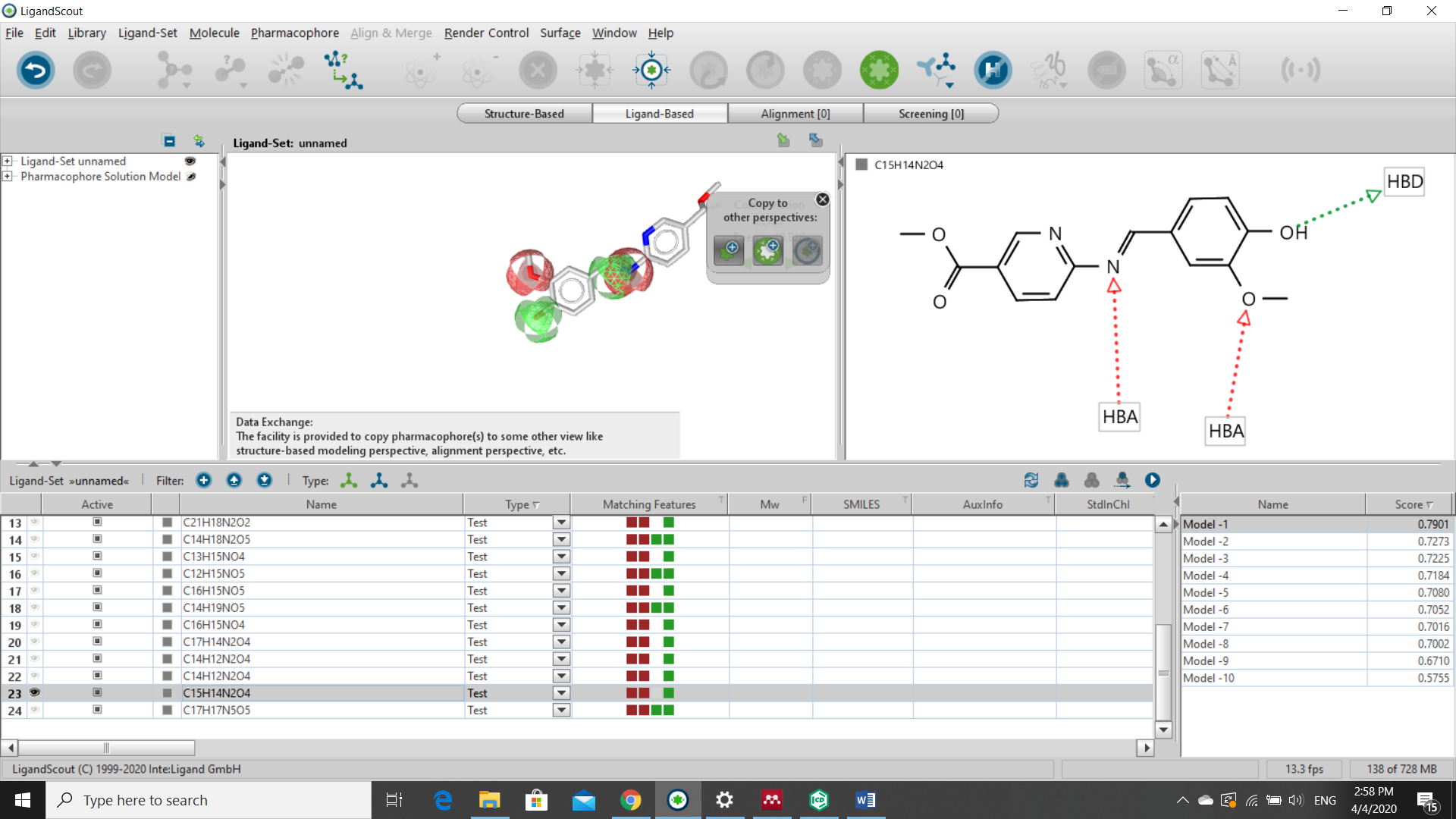 | 46.18 |
| **Tetrodotoxin** | 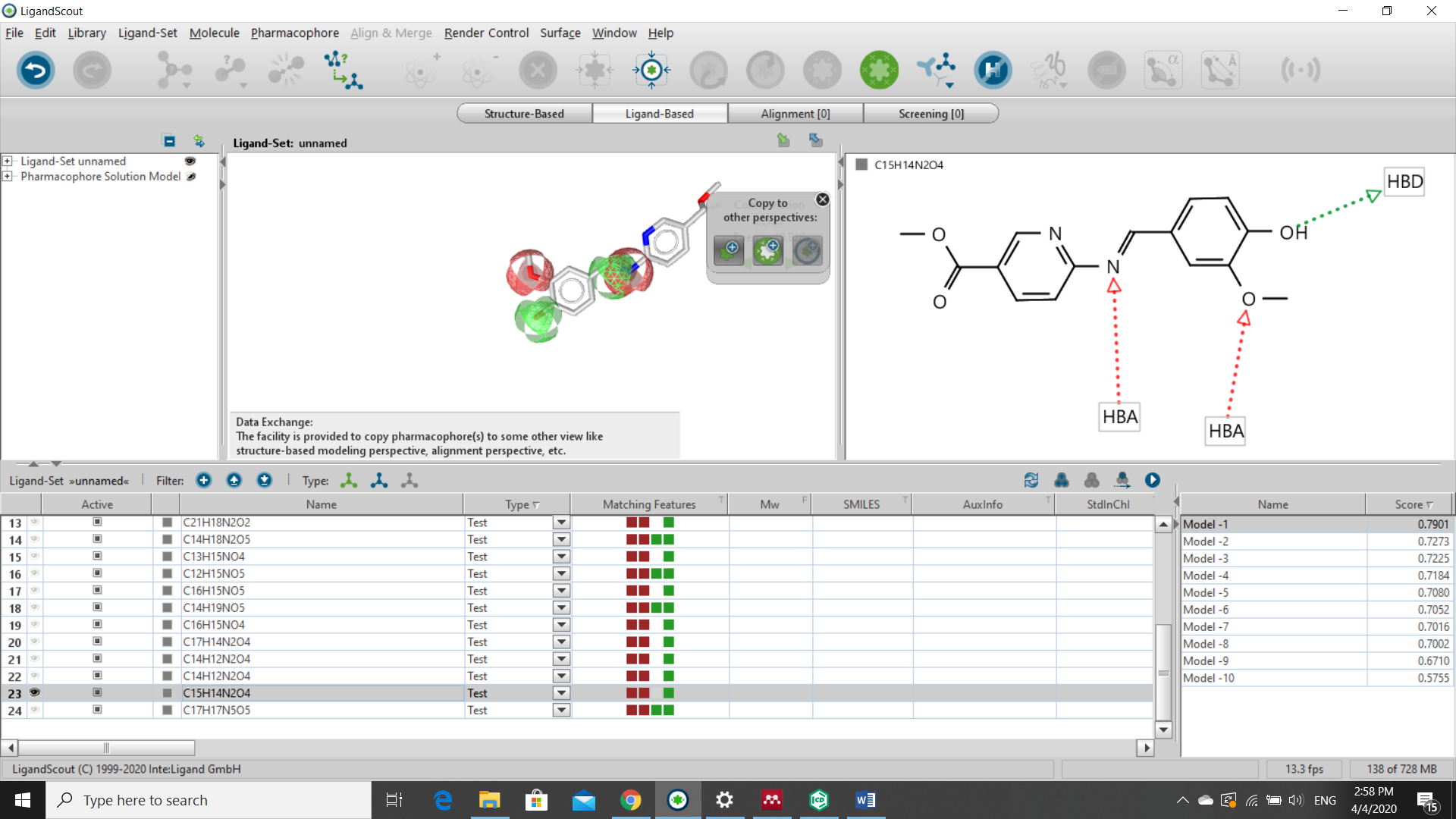 | 43.39 |

**Table S2: Matching features of test sets in the pharmacophore model**

| **Test sets** | **Pharmacophore model of COVID-19 antiviral drugs** | |
| --- | --- | --- |
|  | 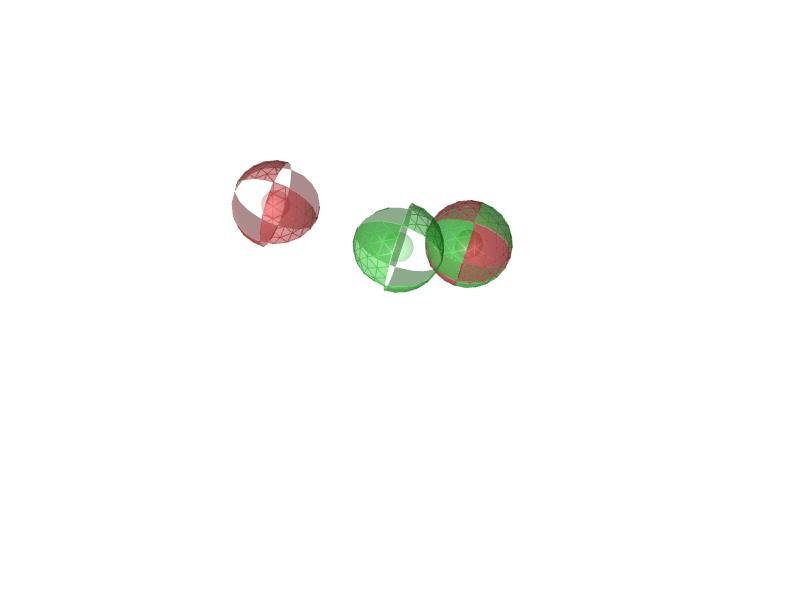  Pharmacophore Model of the Highest Score  2 Hydrogen Bond Acceptor (HBA) and 2 Hydrogen Bond Donor (HBD) | |
| **(1)** | 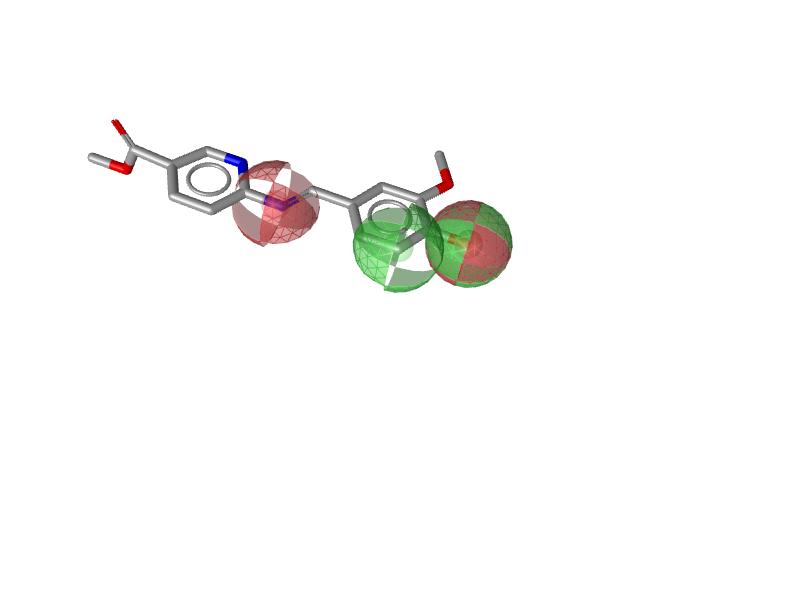 | 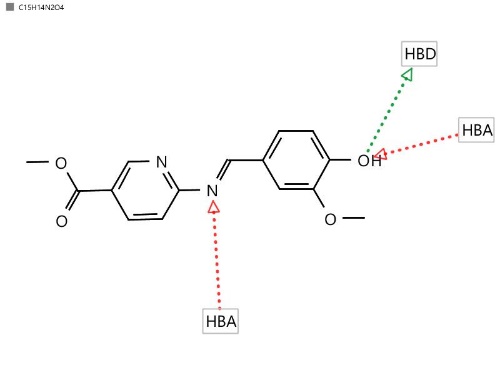 |
| **(2)** | 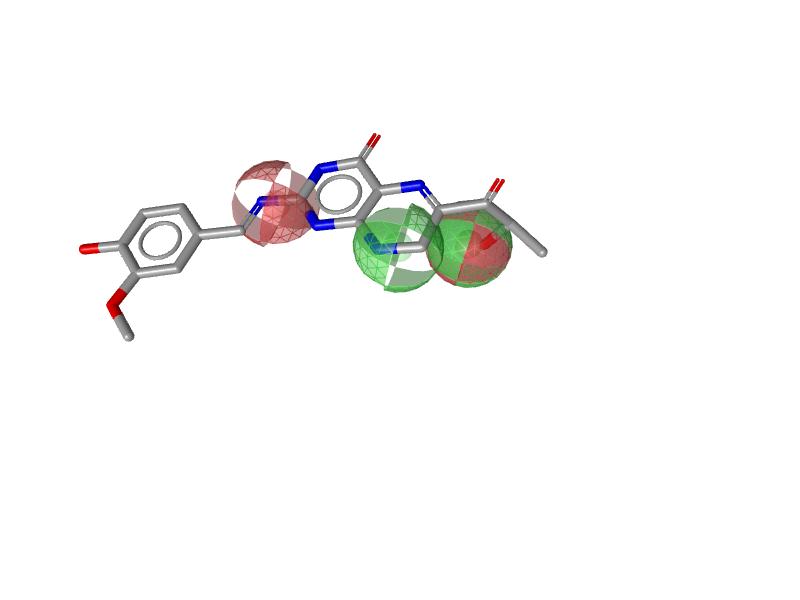 | 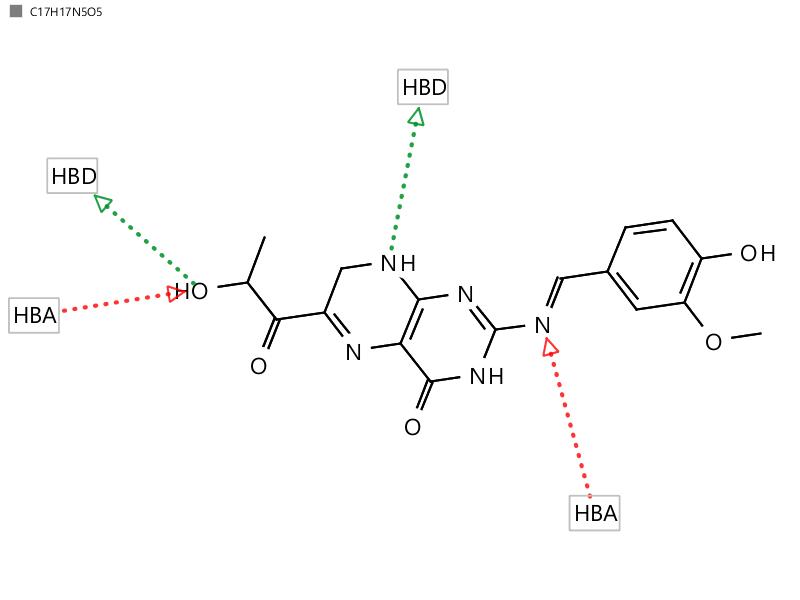 |
| **(3)** | 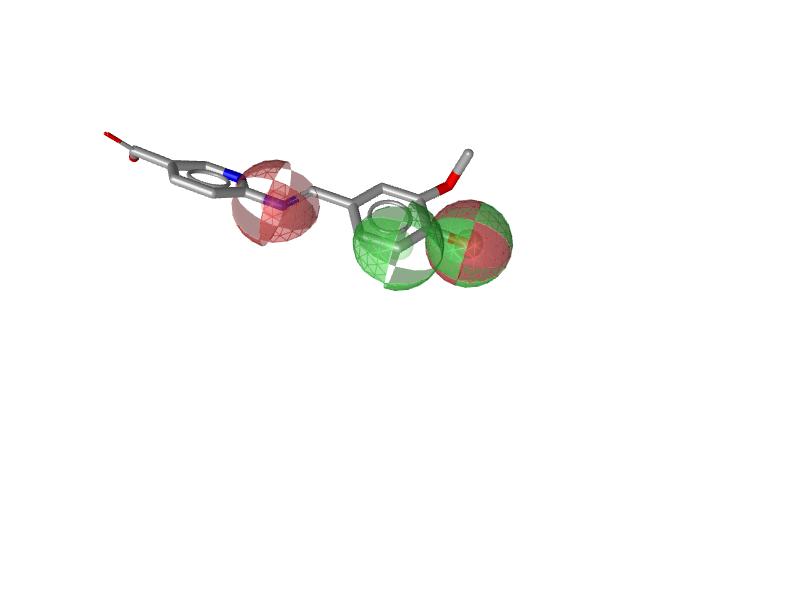 | 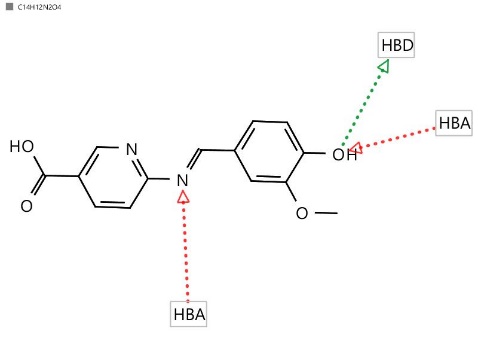 |
| **(4)** | 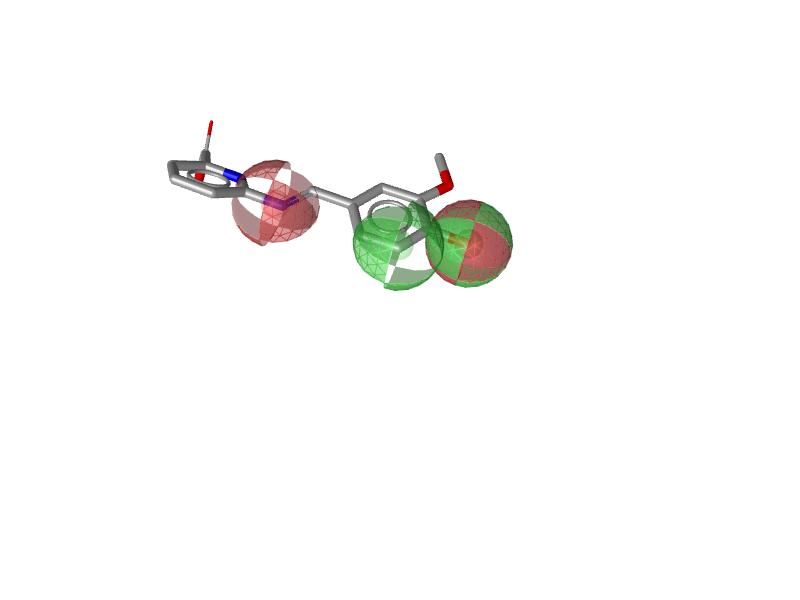 | 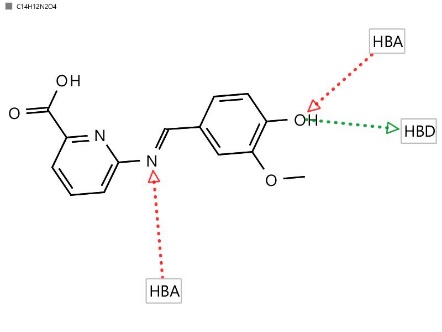 |
| **(5)** | 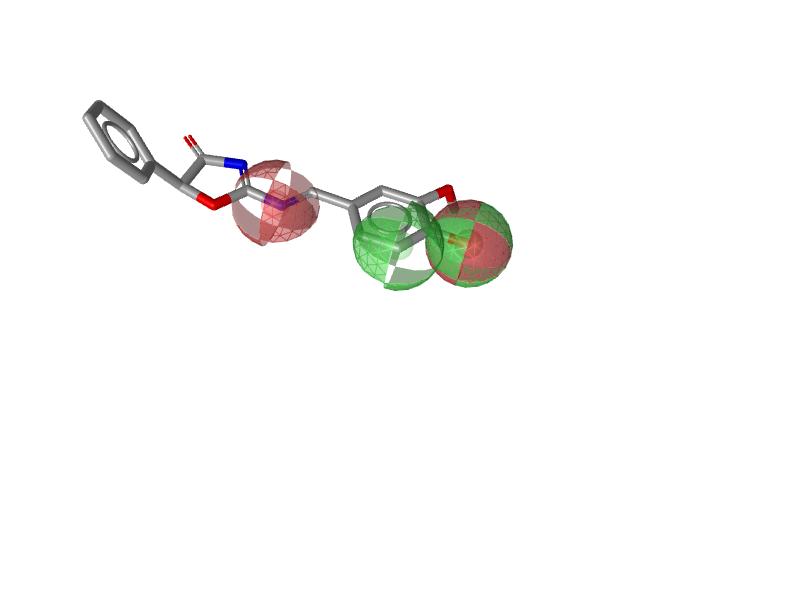 | 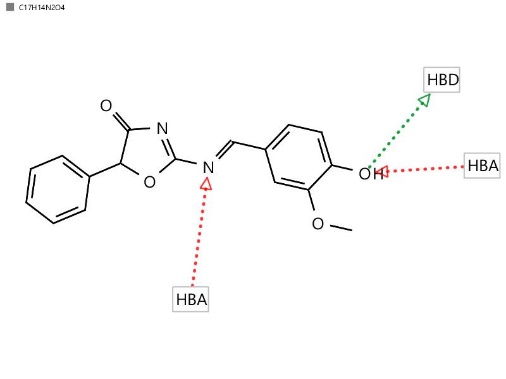 |
| **(6)** | 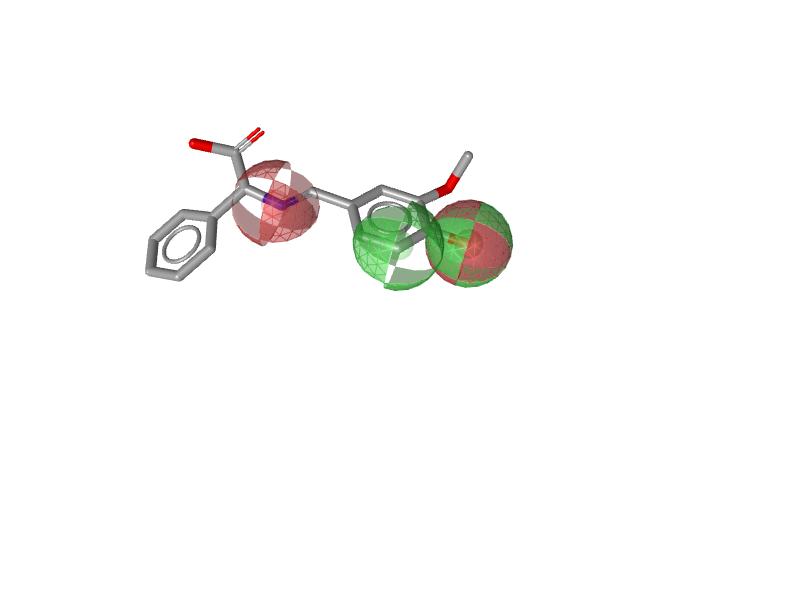 | 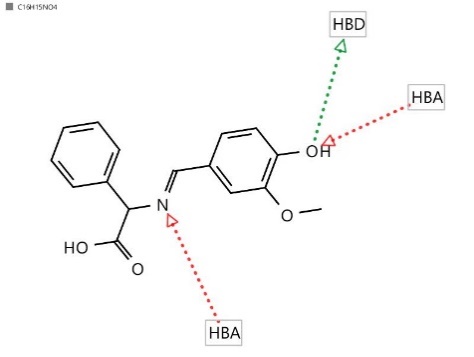 |
| **(7)** | 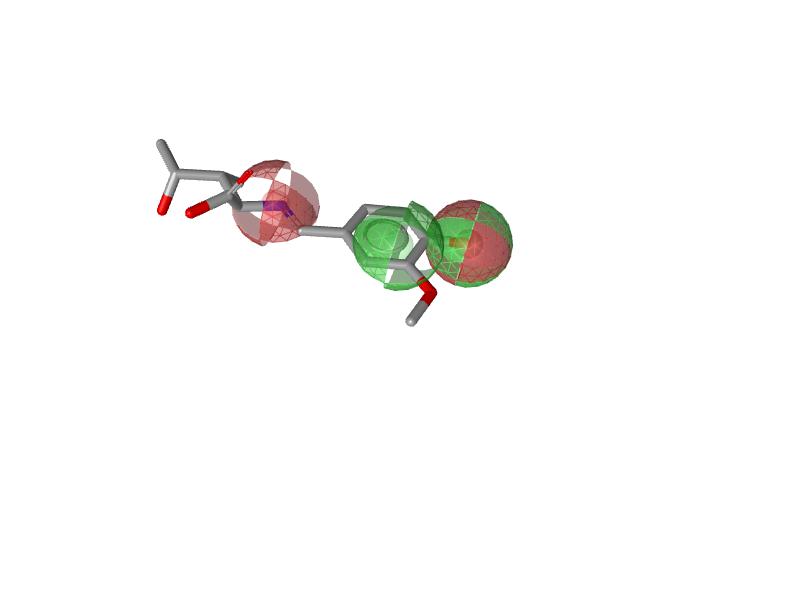 | 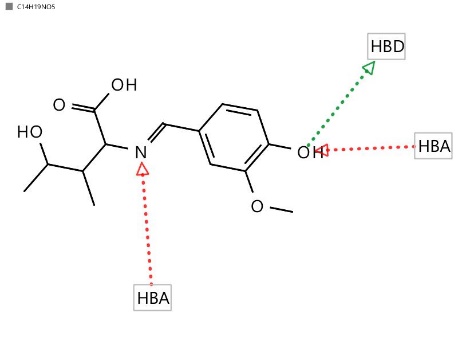 |
| **(8)** | 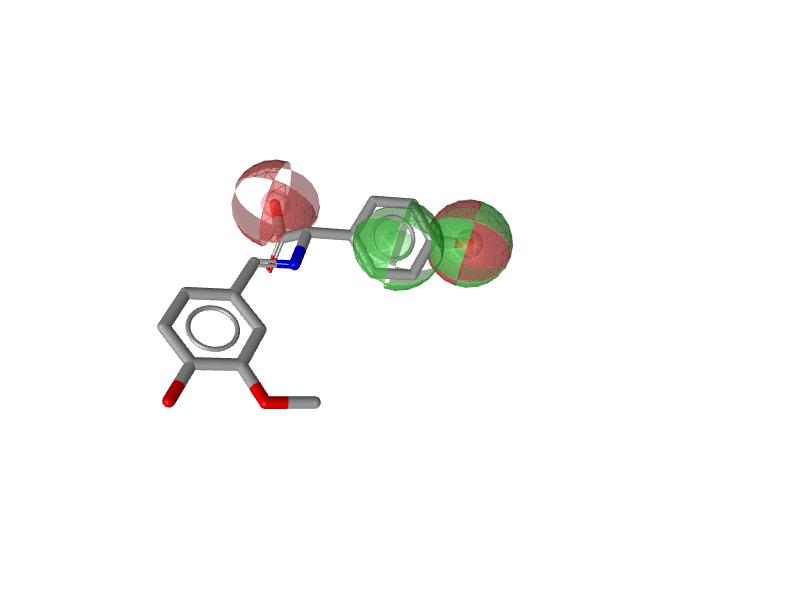 | 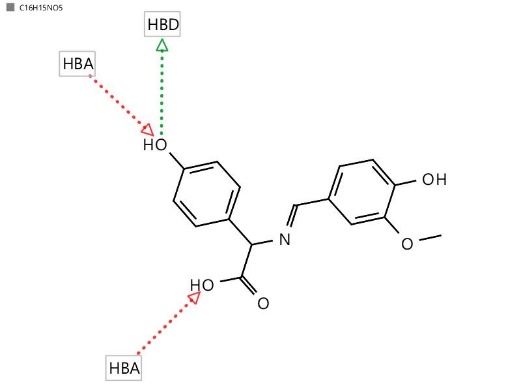 |
| **(9)** | 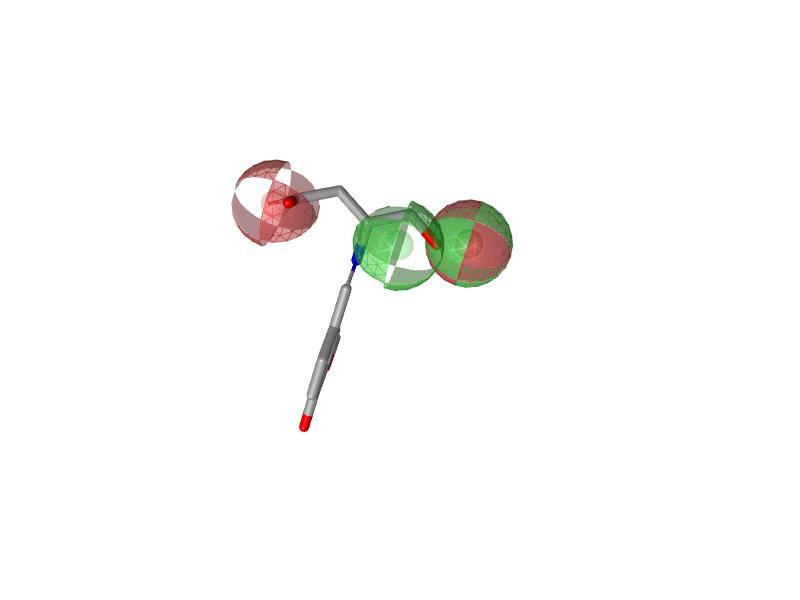 | 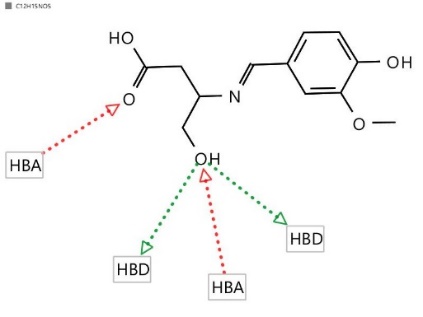 |
| **(10)** | 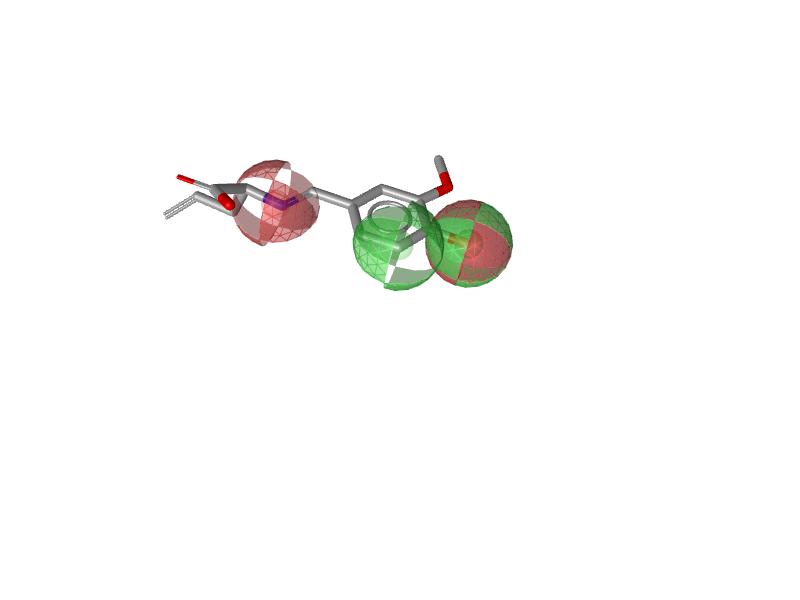 | 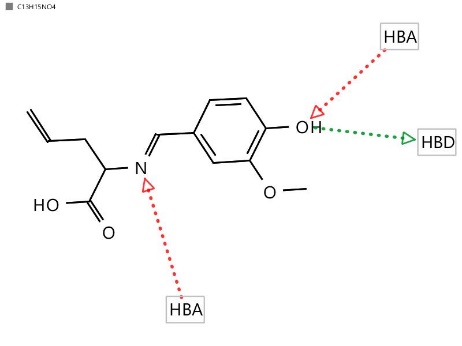 |
| **(11)** | 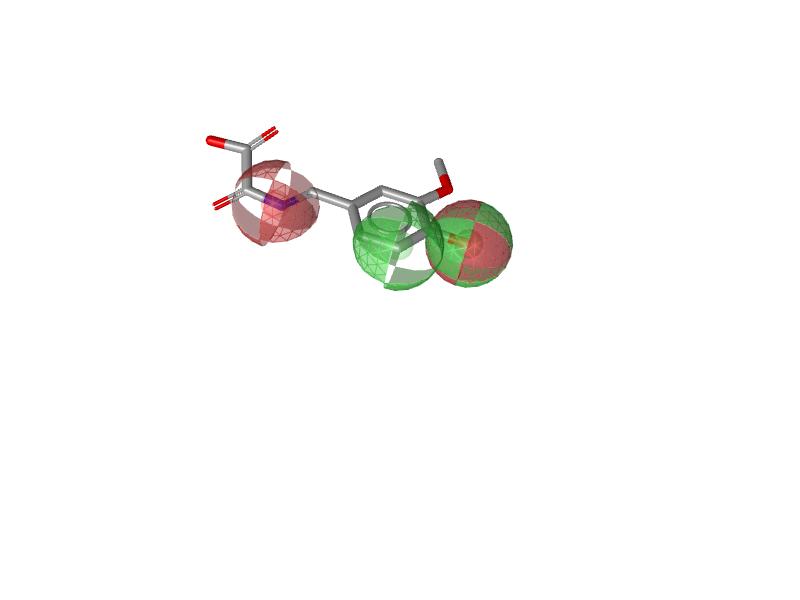 | 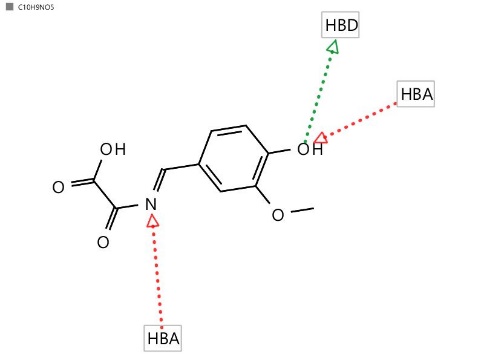 |
| **(12)** | 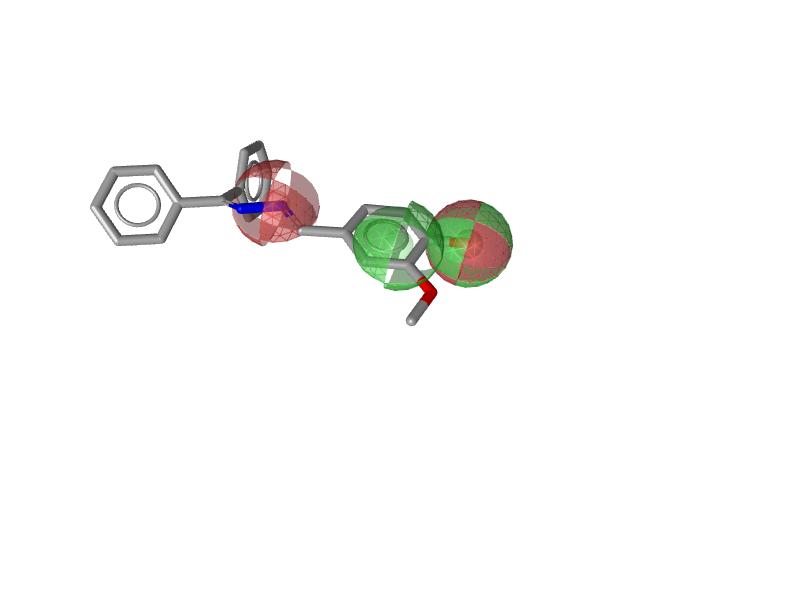 | 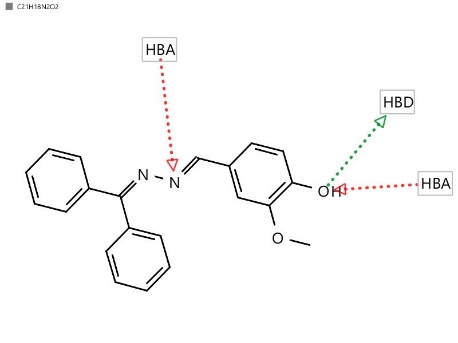 |
| **(13)** | 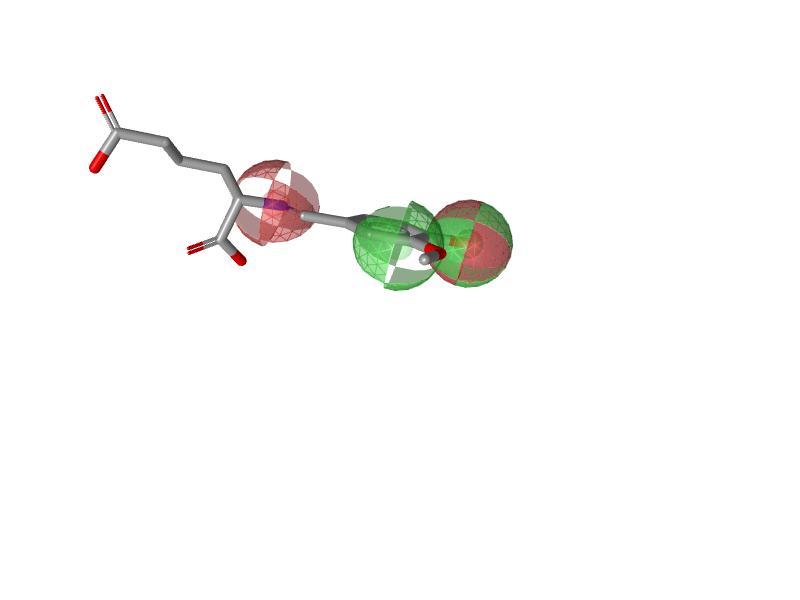 | 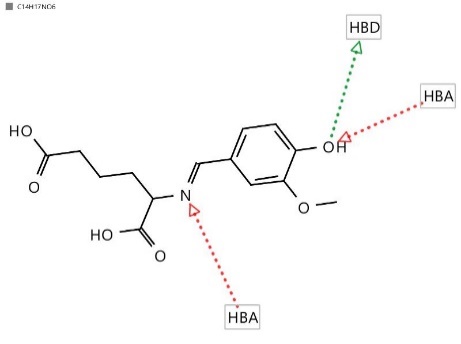 |
| **(14)** | 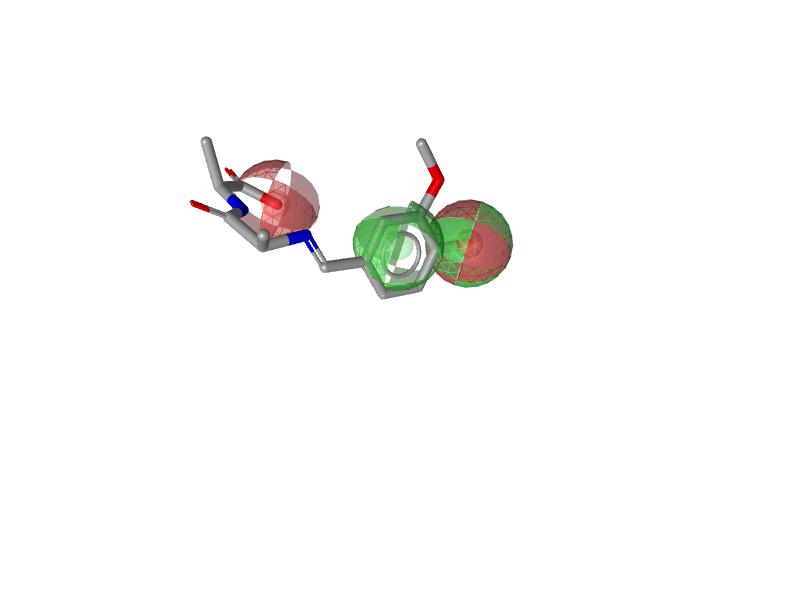 | 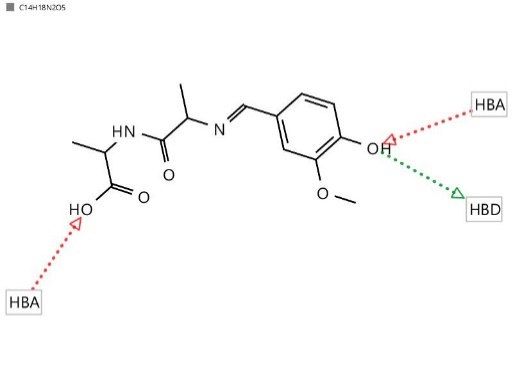 |
| **(15)** | 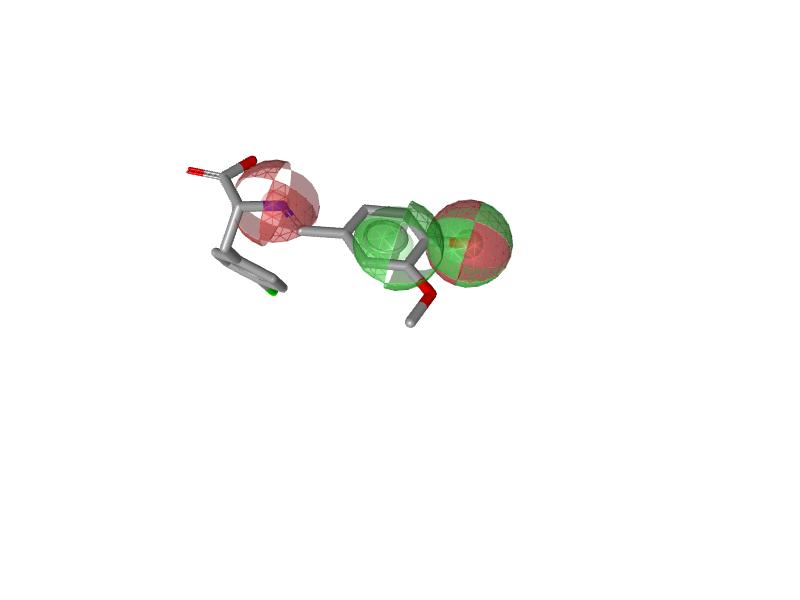 | 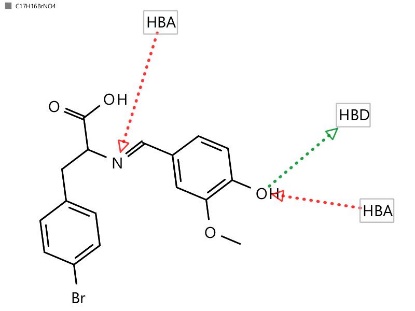 |
| **(16)** | 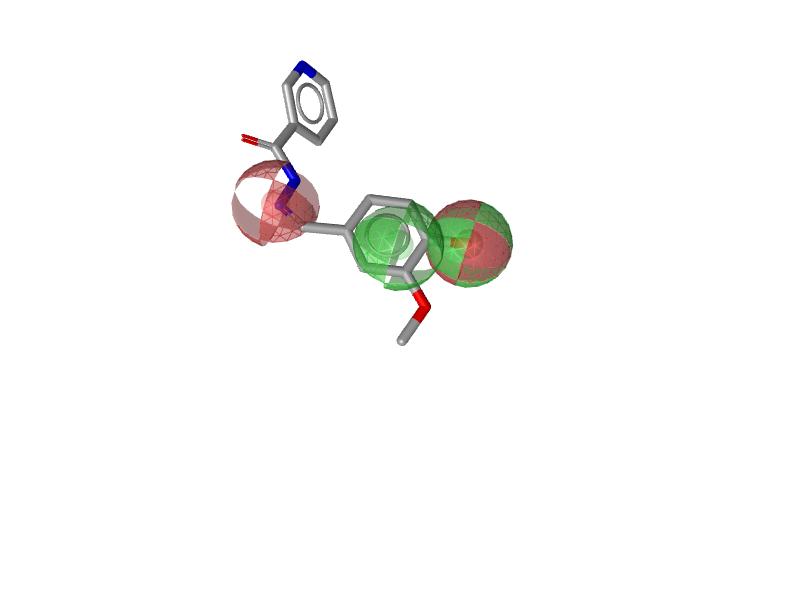 | 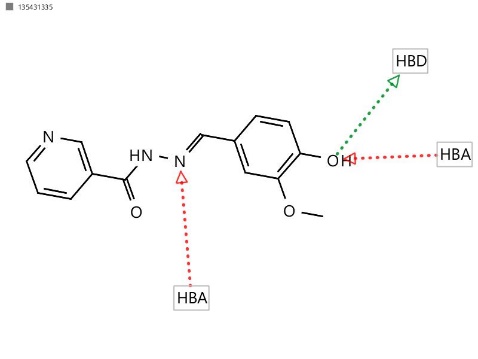 |
| **(17)** | 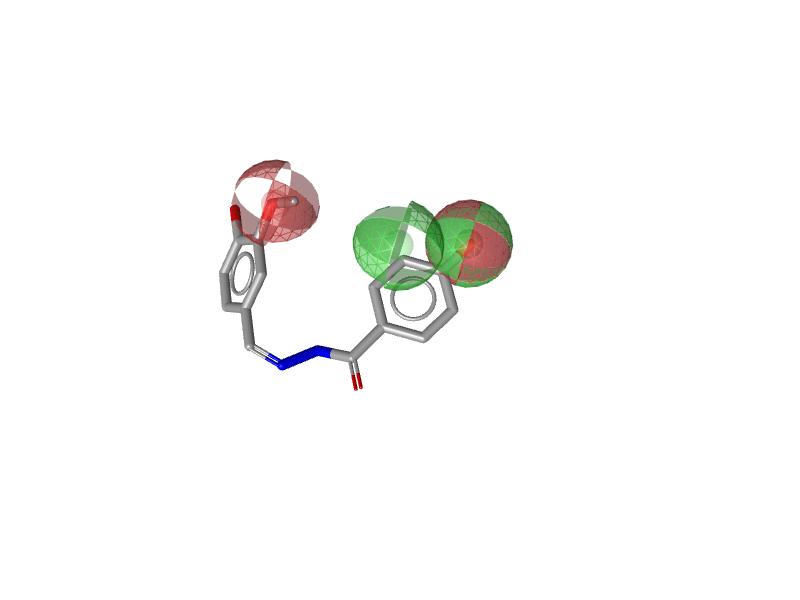 | 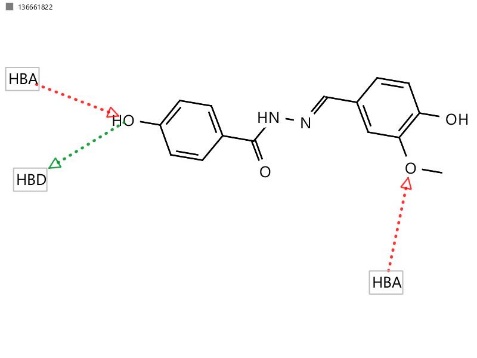 |
| **(18)** | 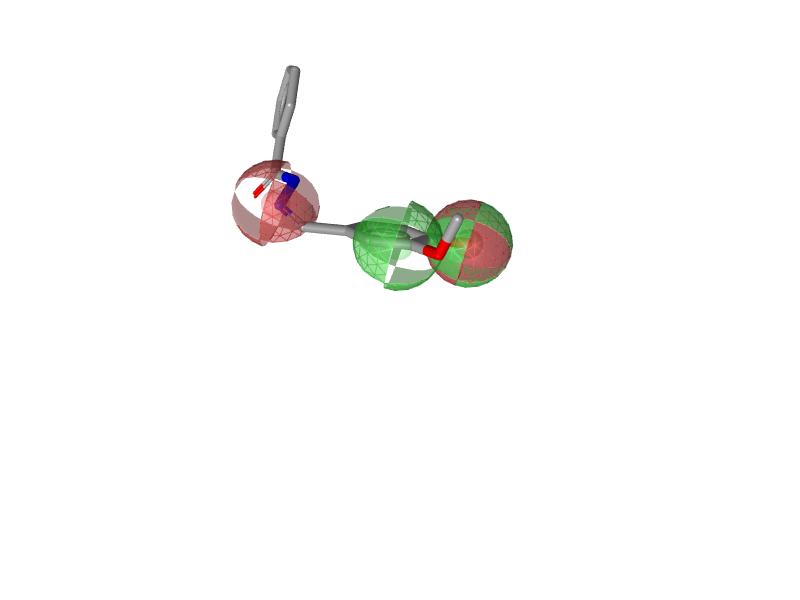 | 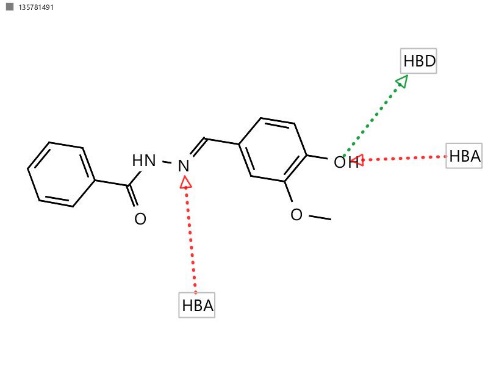 |
| **(19)** | 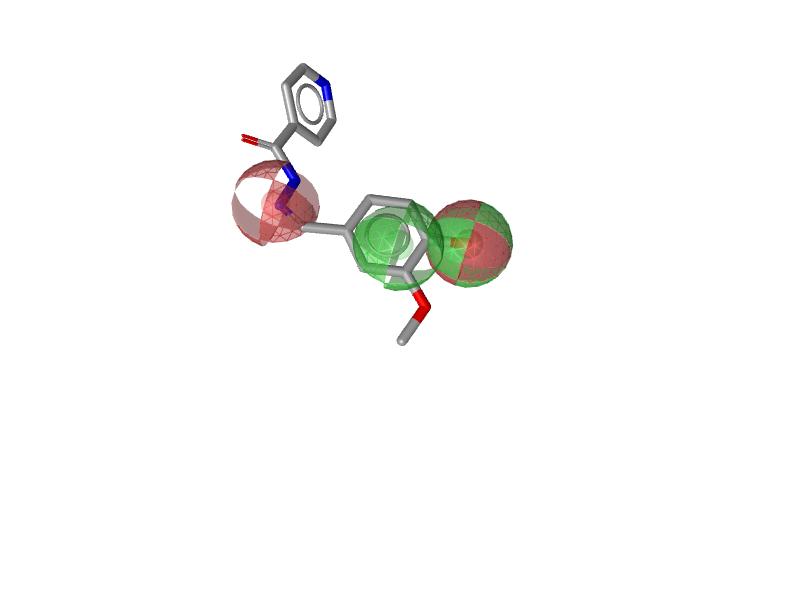 | 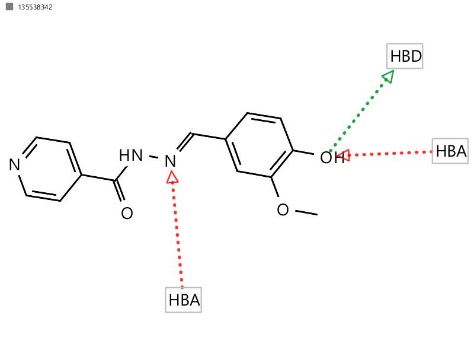 |
| **(20)** | 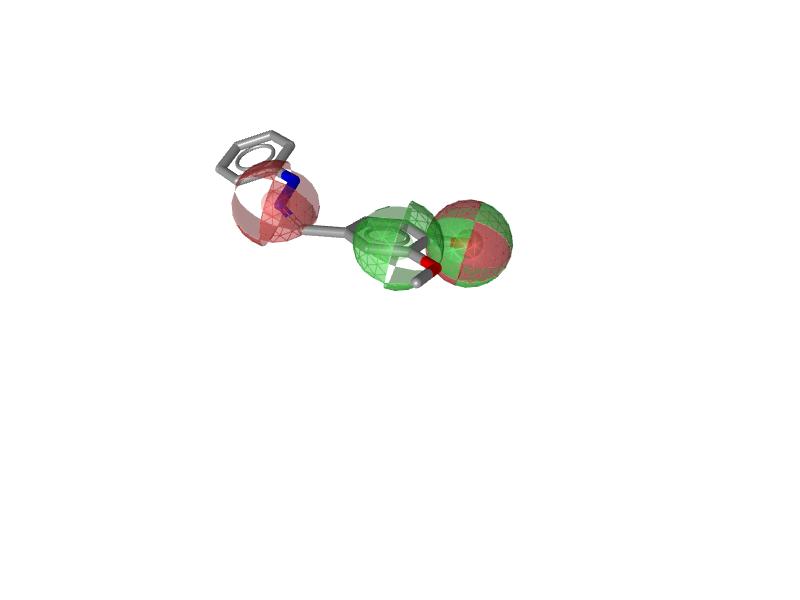 | 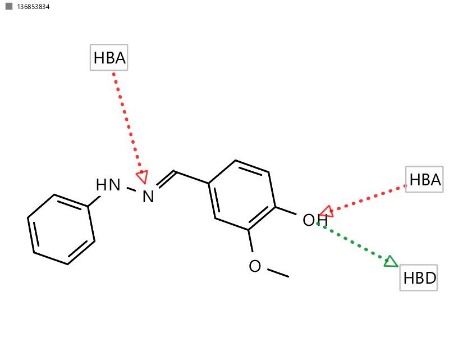 |
| **(21)** | 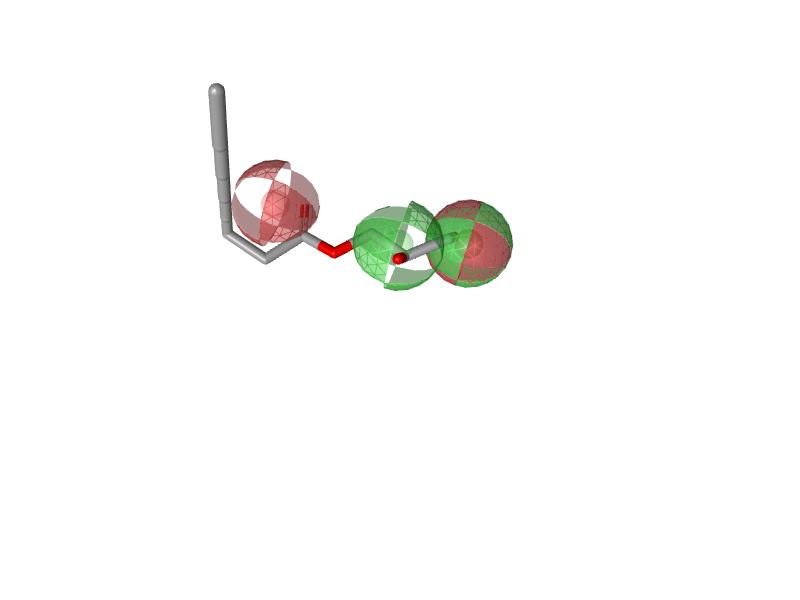 | 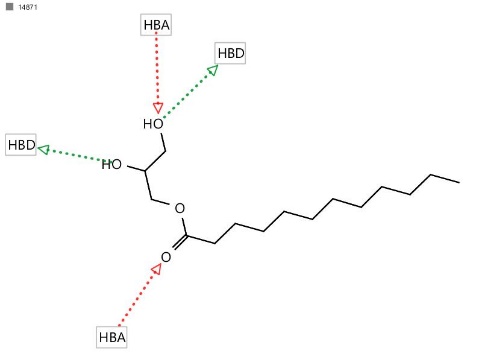 |
| **(22)** | **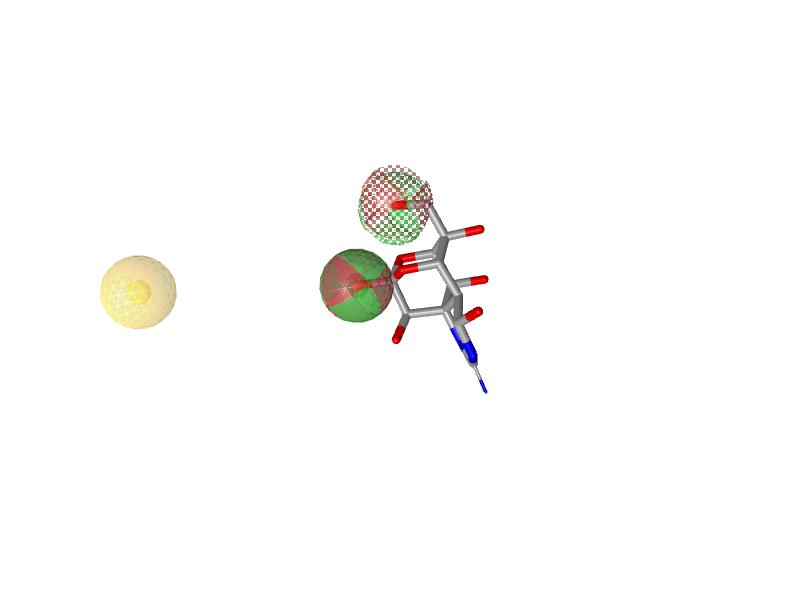** | **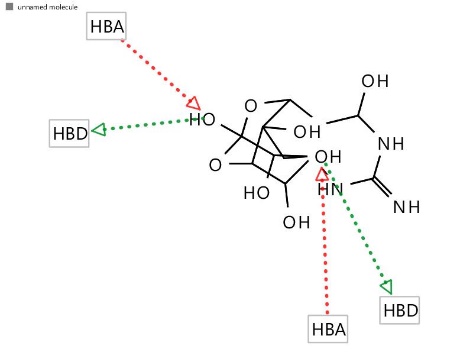** |

**Table S3: Alignment scores and matching features of vanillin derivatives in the active sites of 5RE6**

| **Test sets** | **PDB (Code: 5RE6)** | |
| --- | --- | --- |
|  | **Z54571979 / N-{4-[(pyrimidin-2-yl)oxy]phenyl}acetamide in the active site**  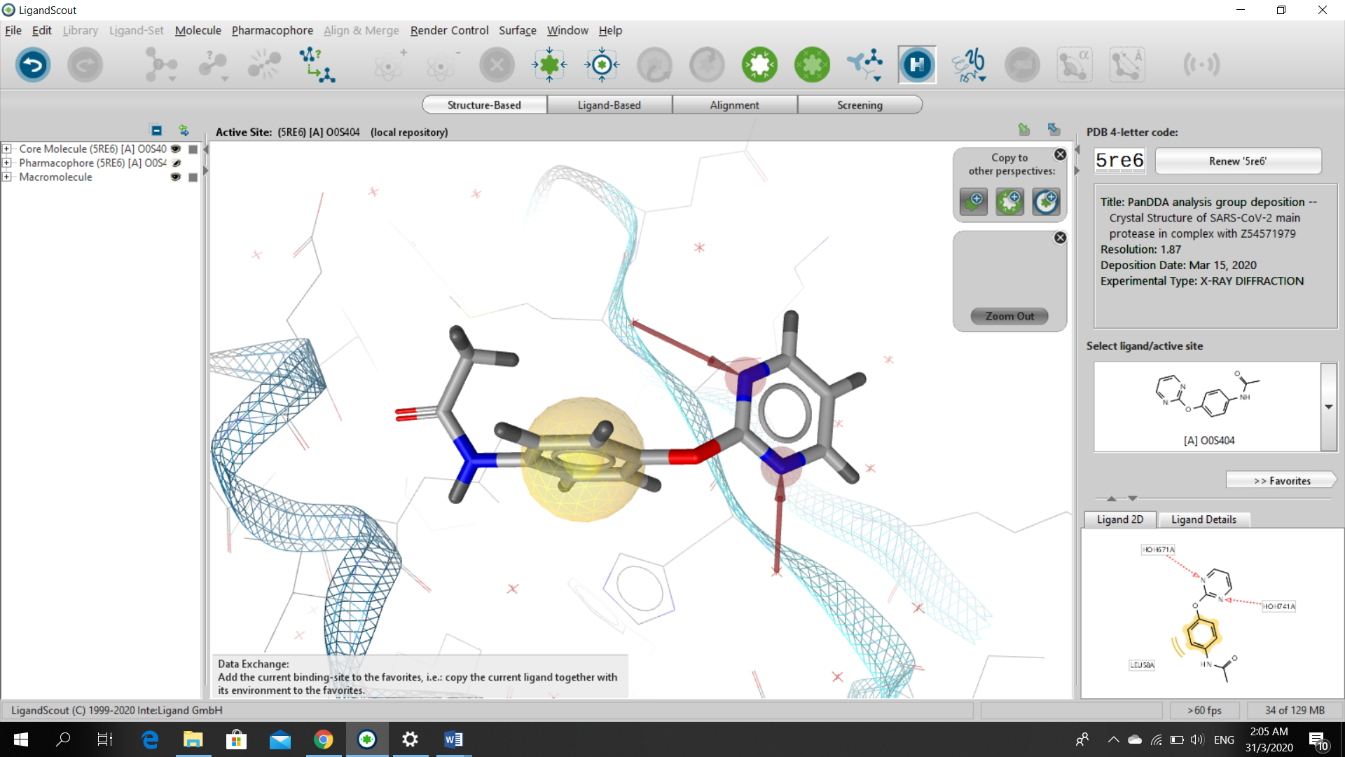 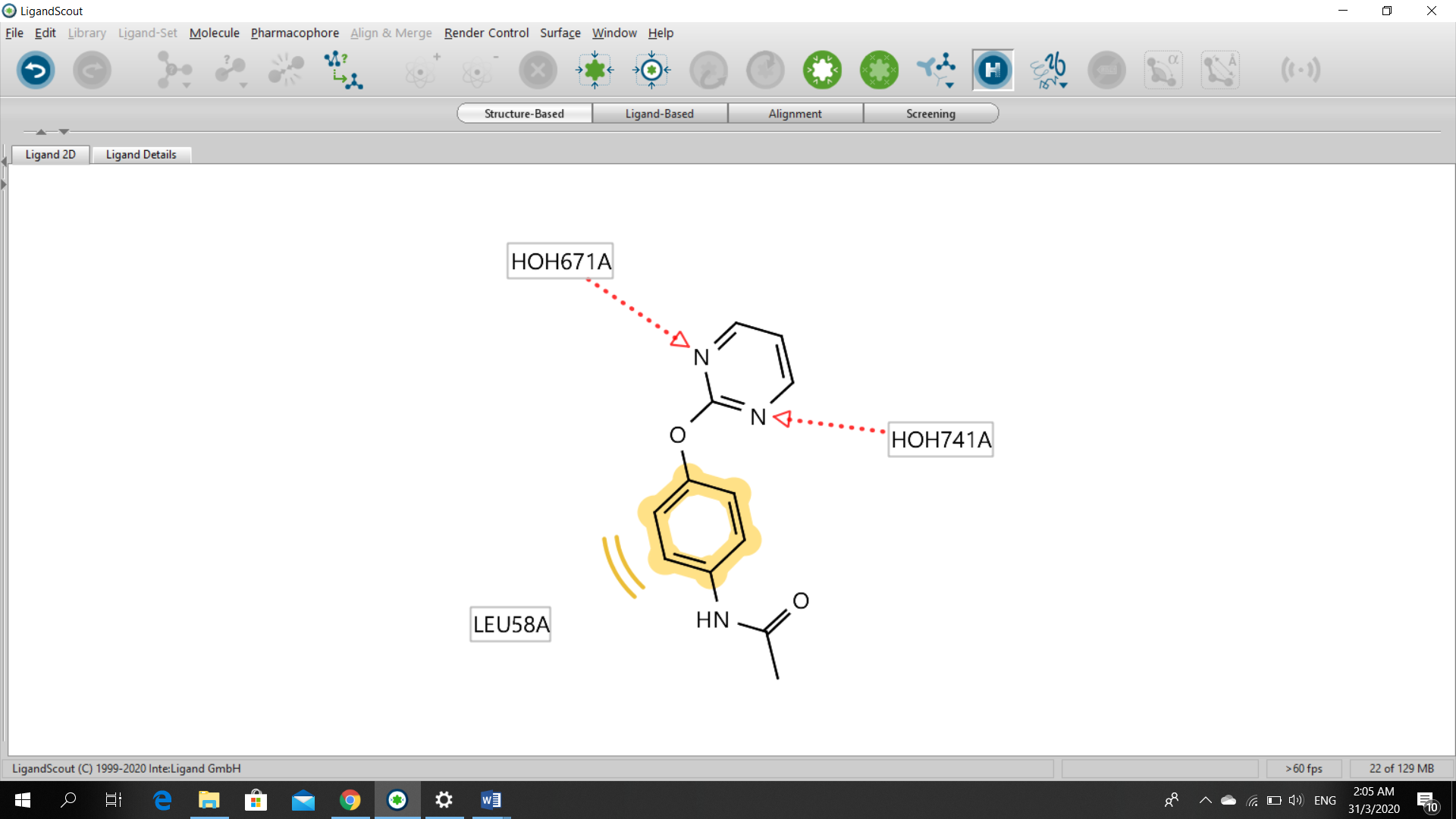 | |
| **(1)** | 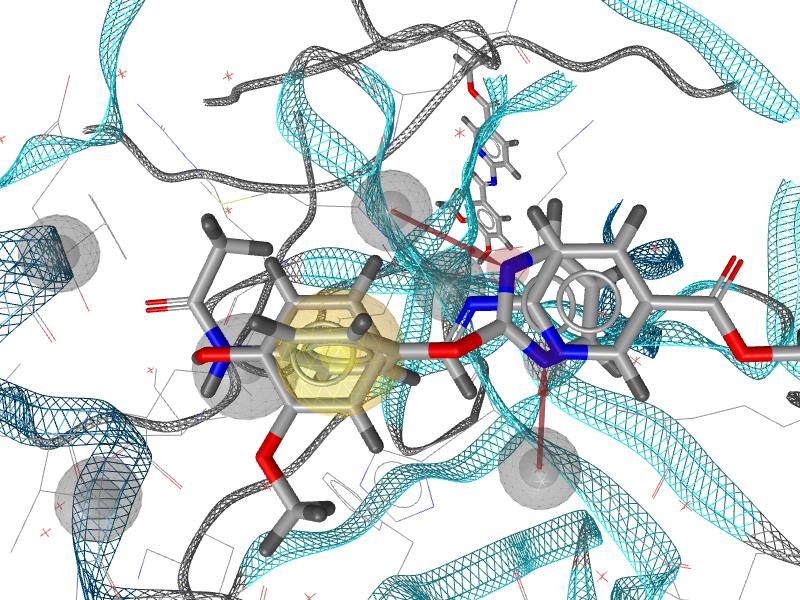  Alignment score: 36.97 | 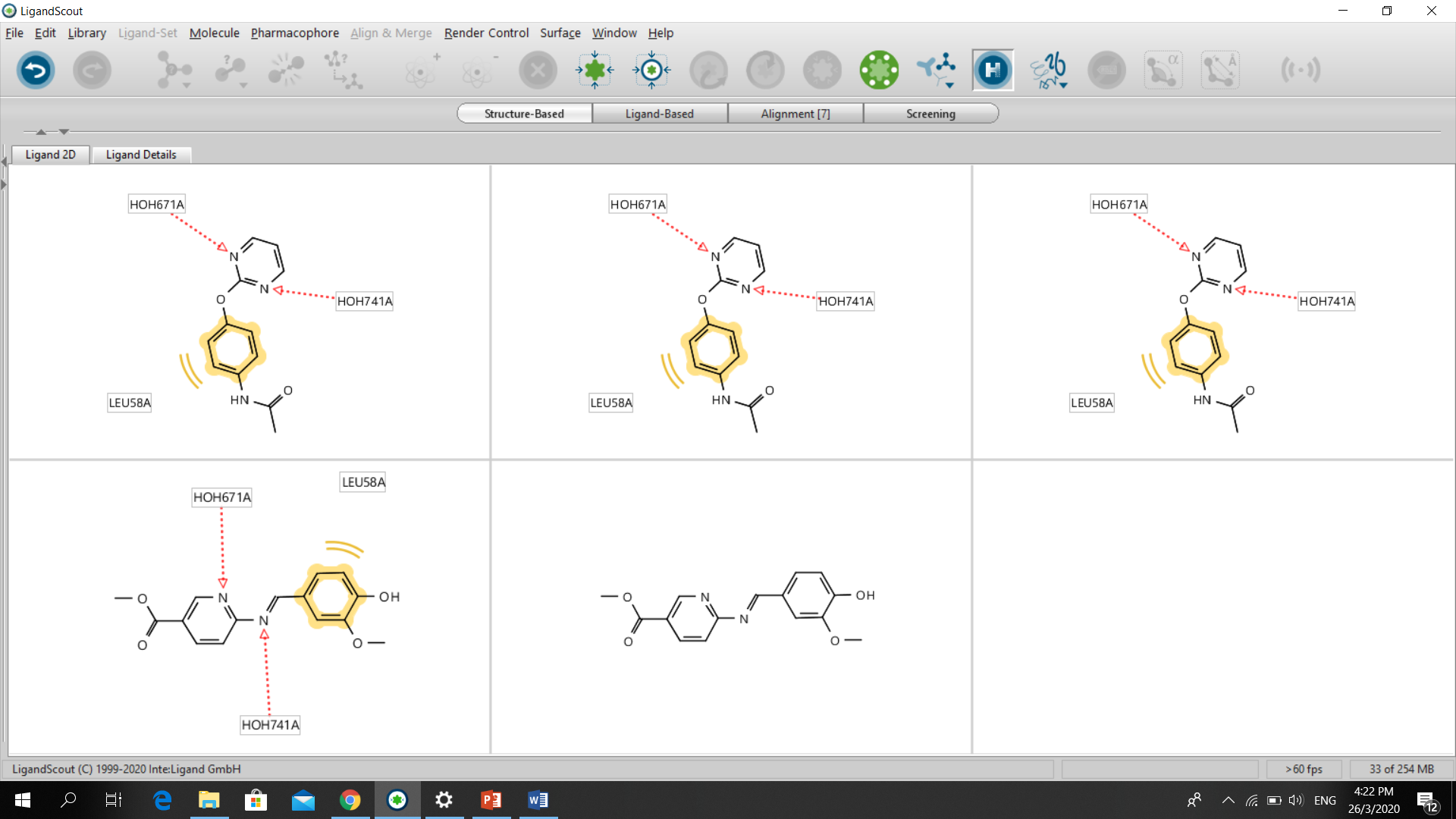 |
| **(2)** | 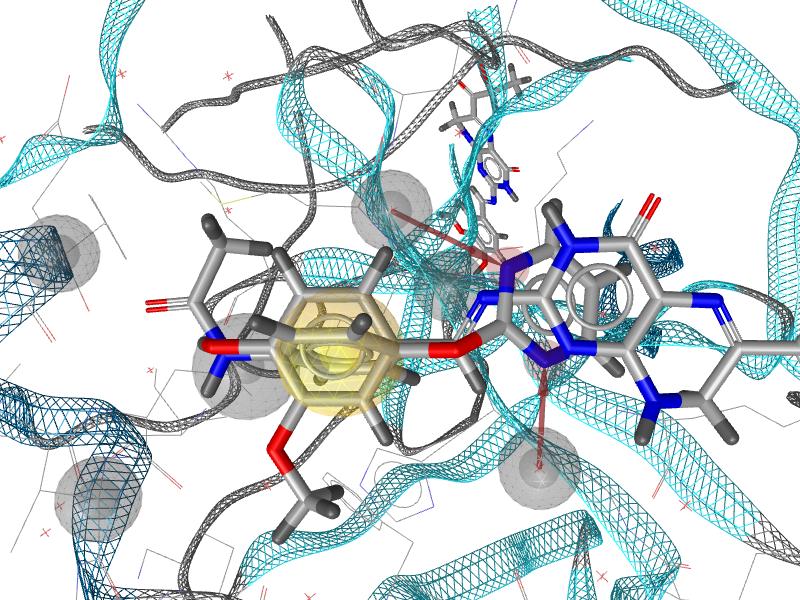  Alignment score: 37.01 | 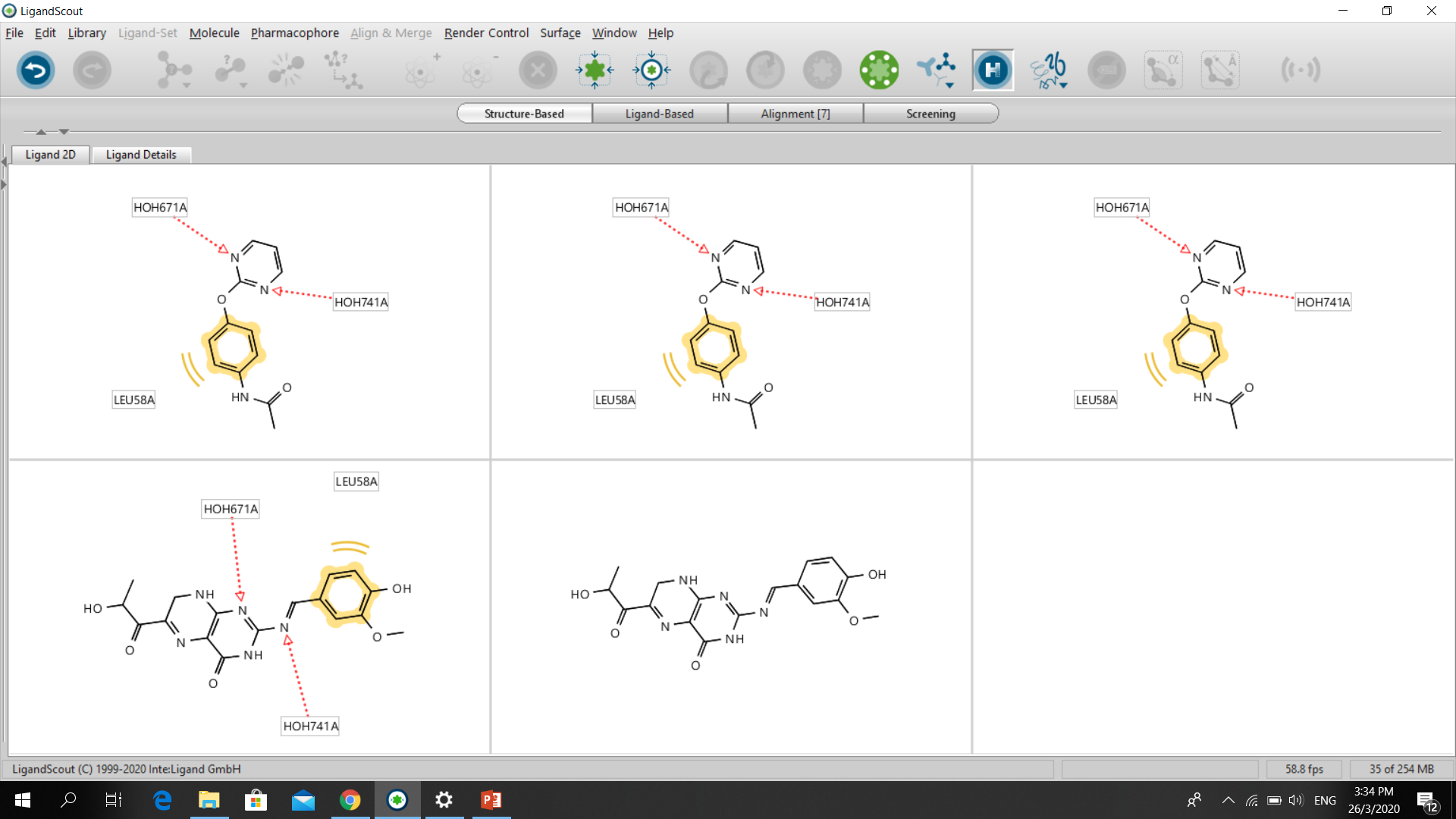 |
| **(3)** | 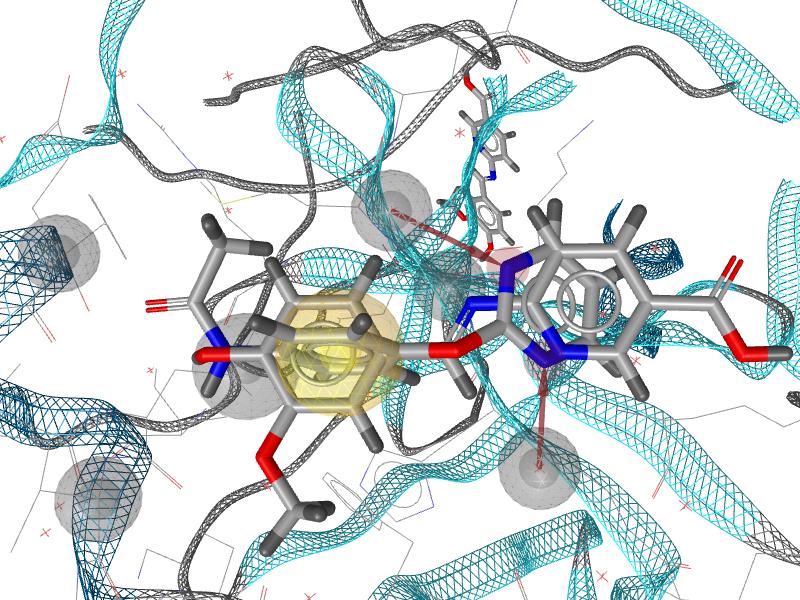  Alignment score: 36.97 | 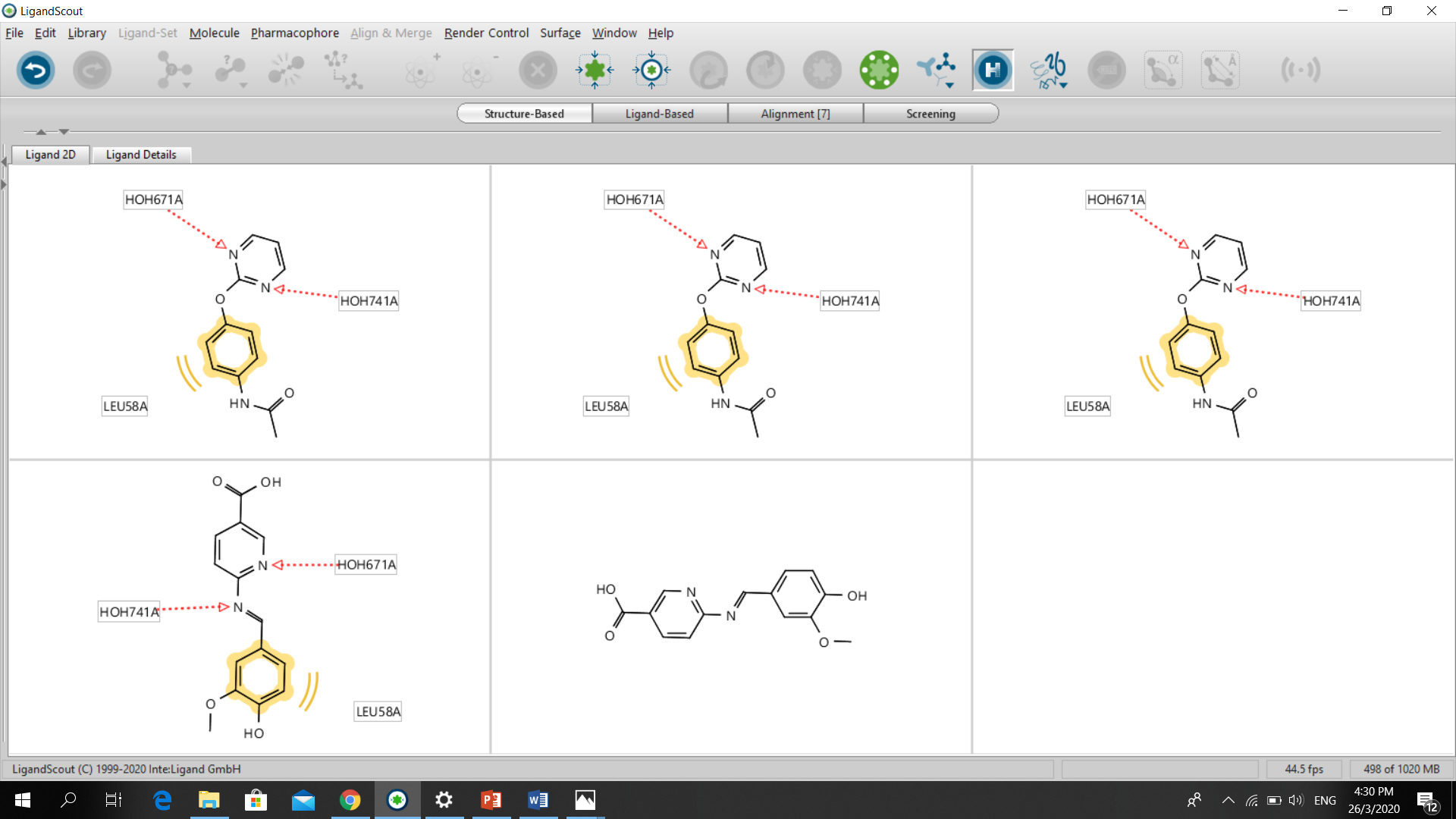 |
| **(4)** | 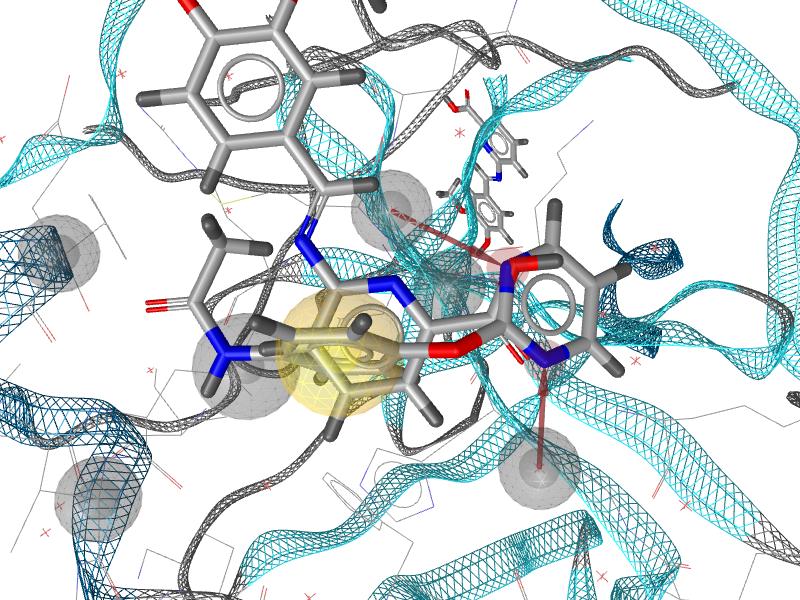  Alignment score: 37.96 | 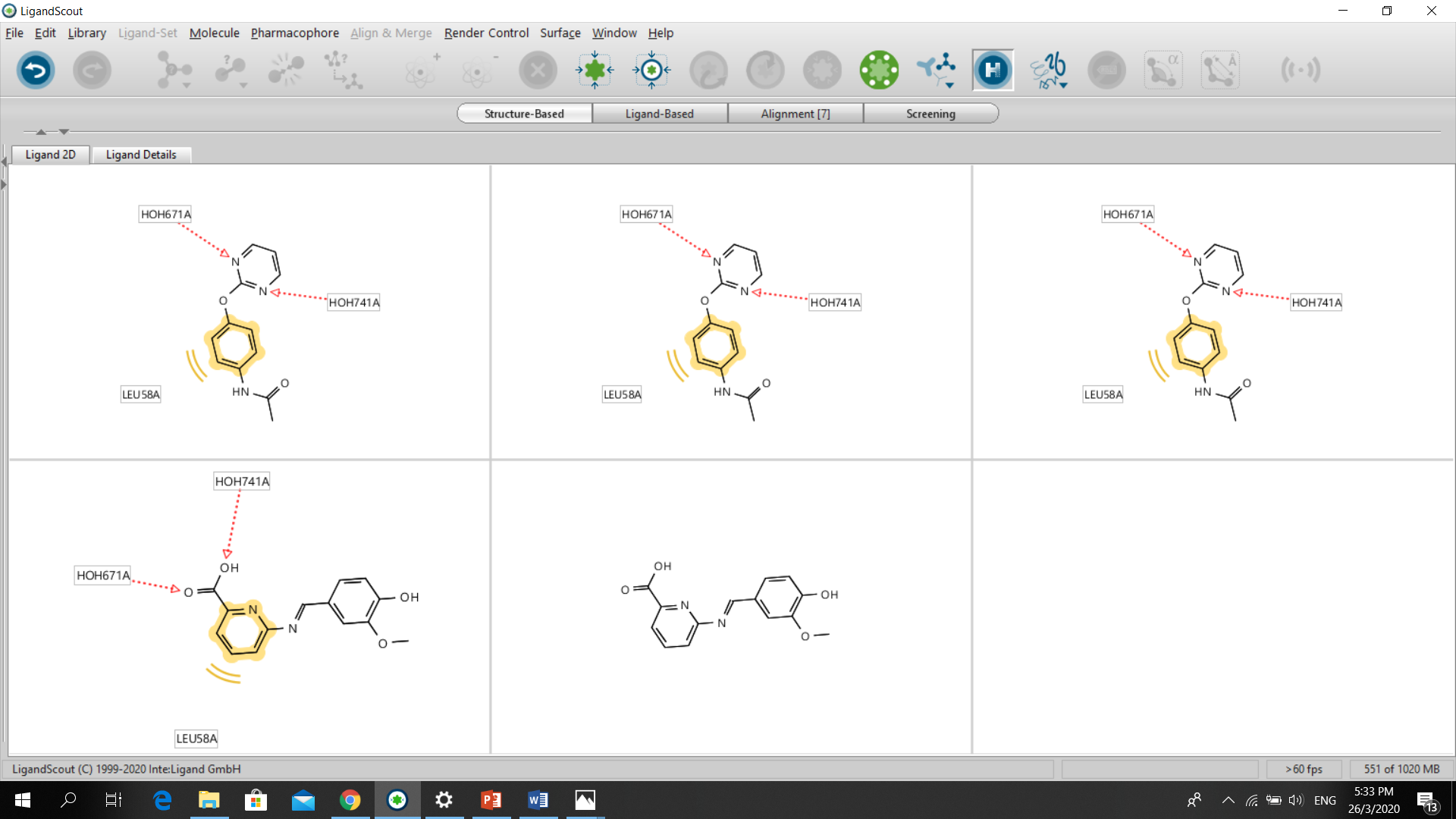 |
| **(5)** | 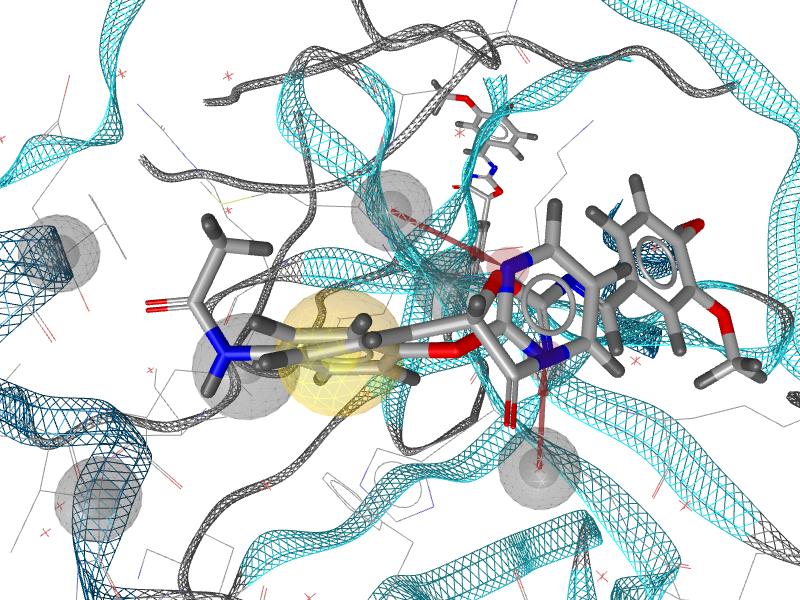  Alignment score: 38.33 | 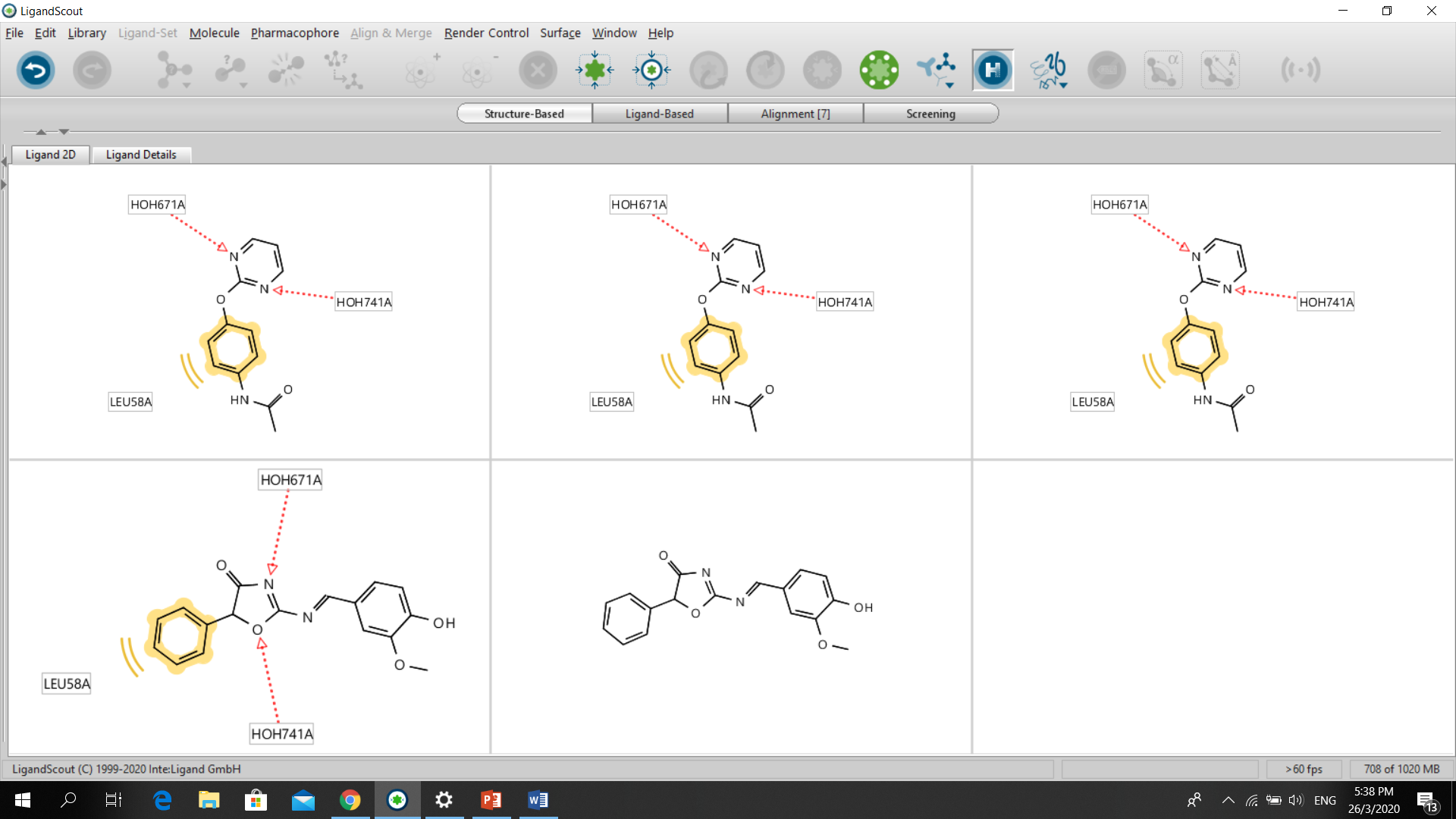 |
| **(6)** | 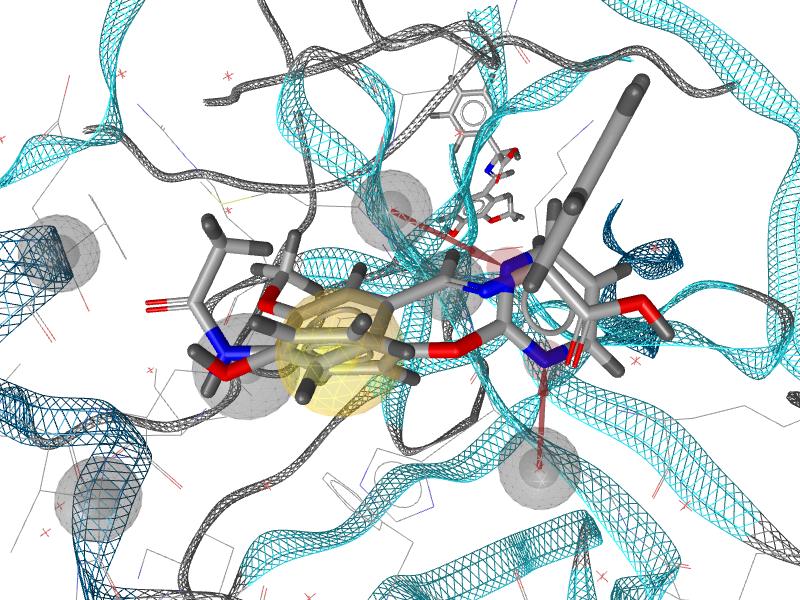  Alignment score: 38.27 | 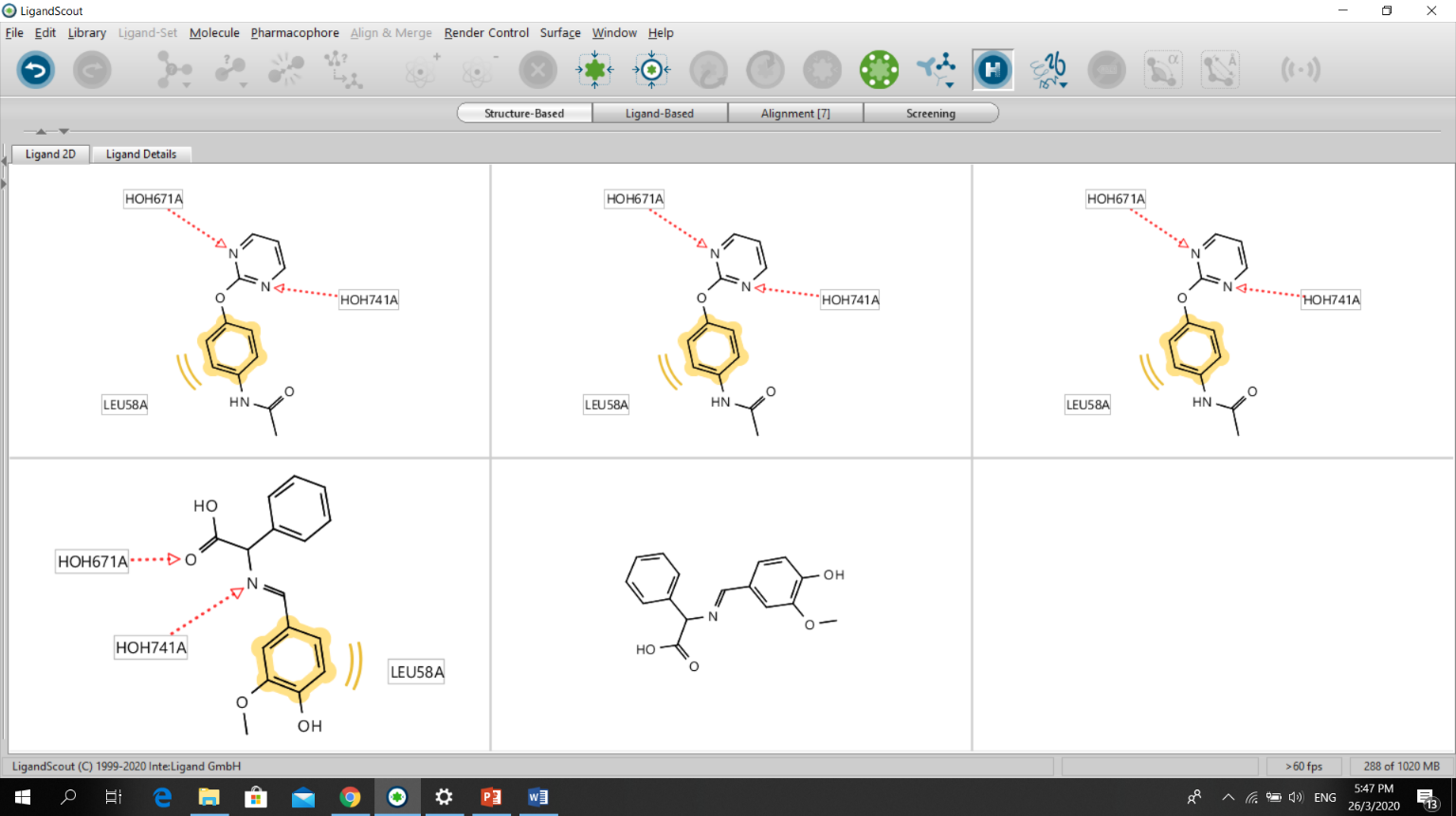 |
| **(7)** | 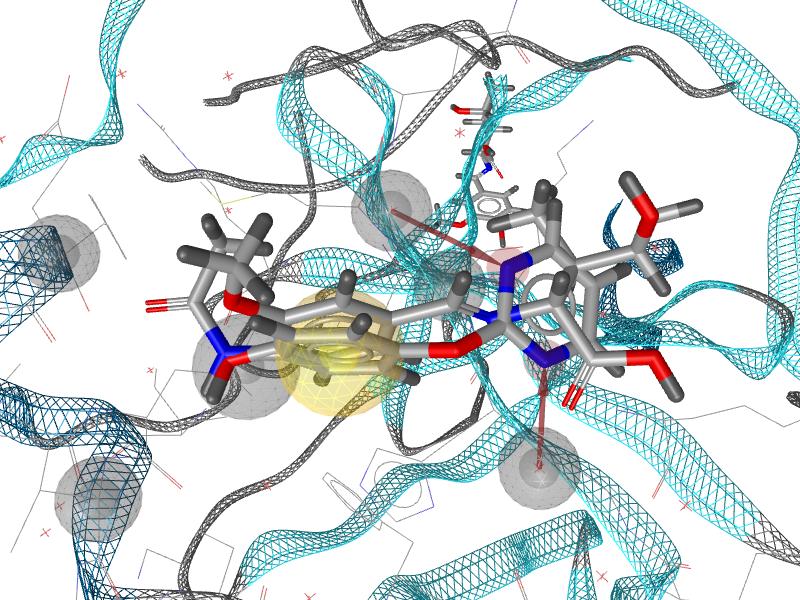  Alignment score: 37.81 | 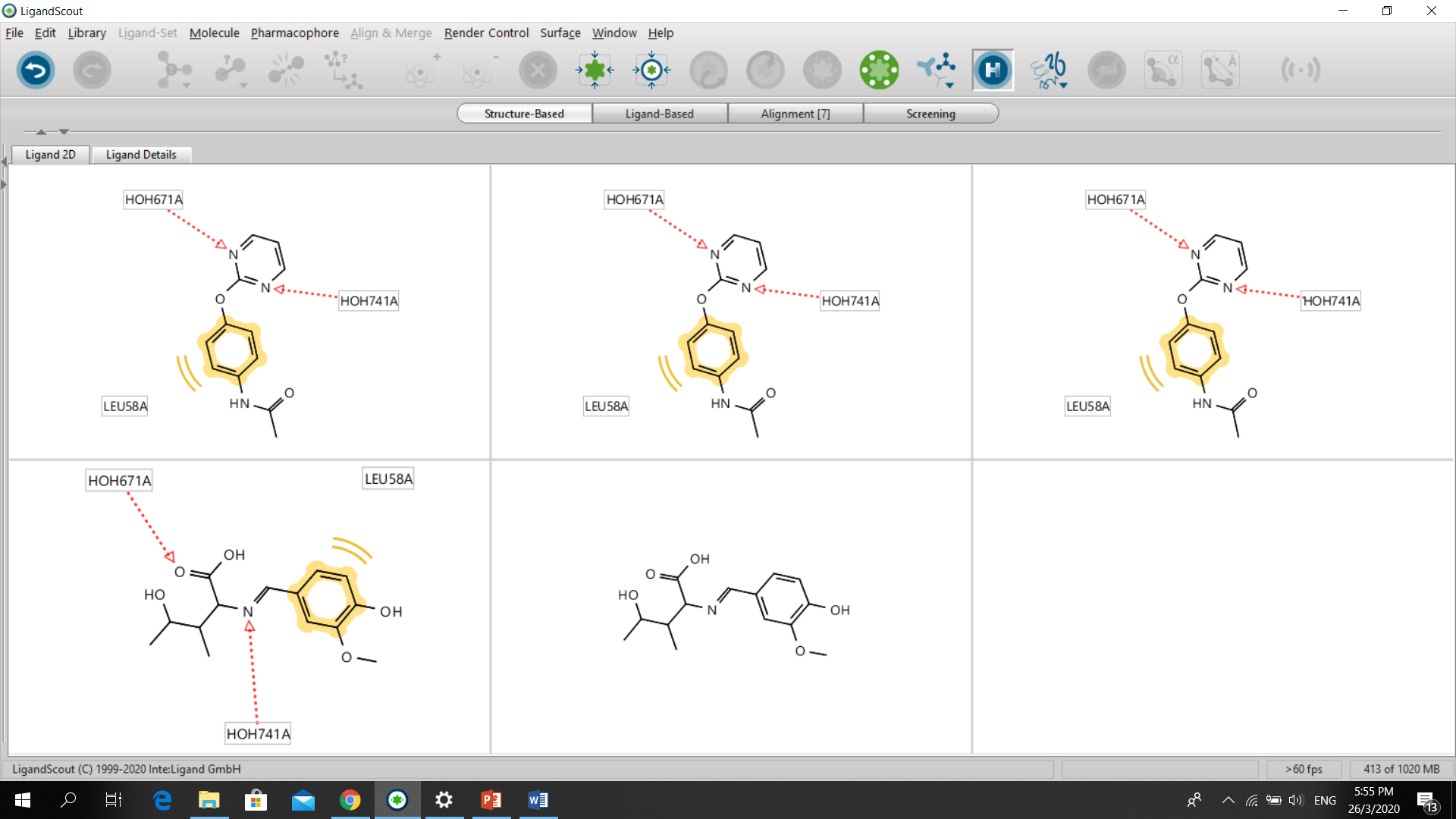 |
| **(8)** | 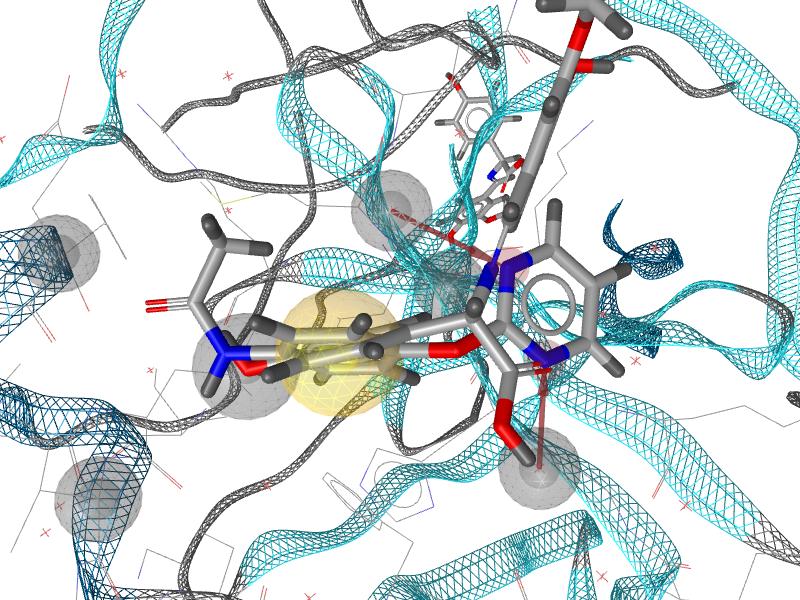  Alignment score: 38.06 | 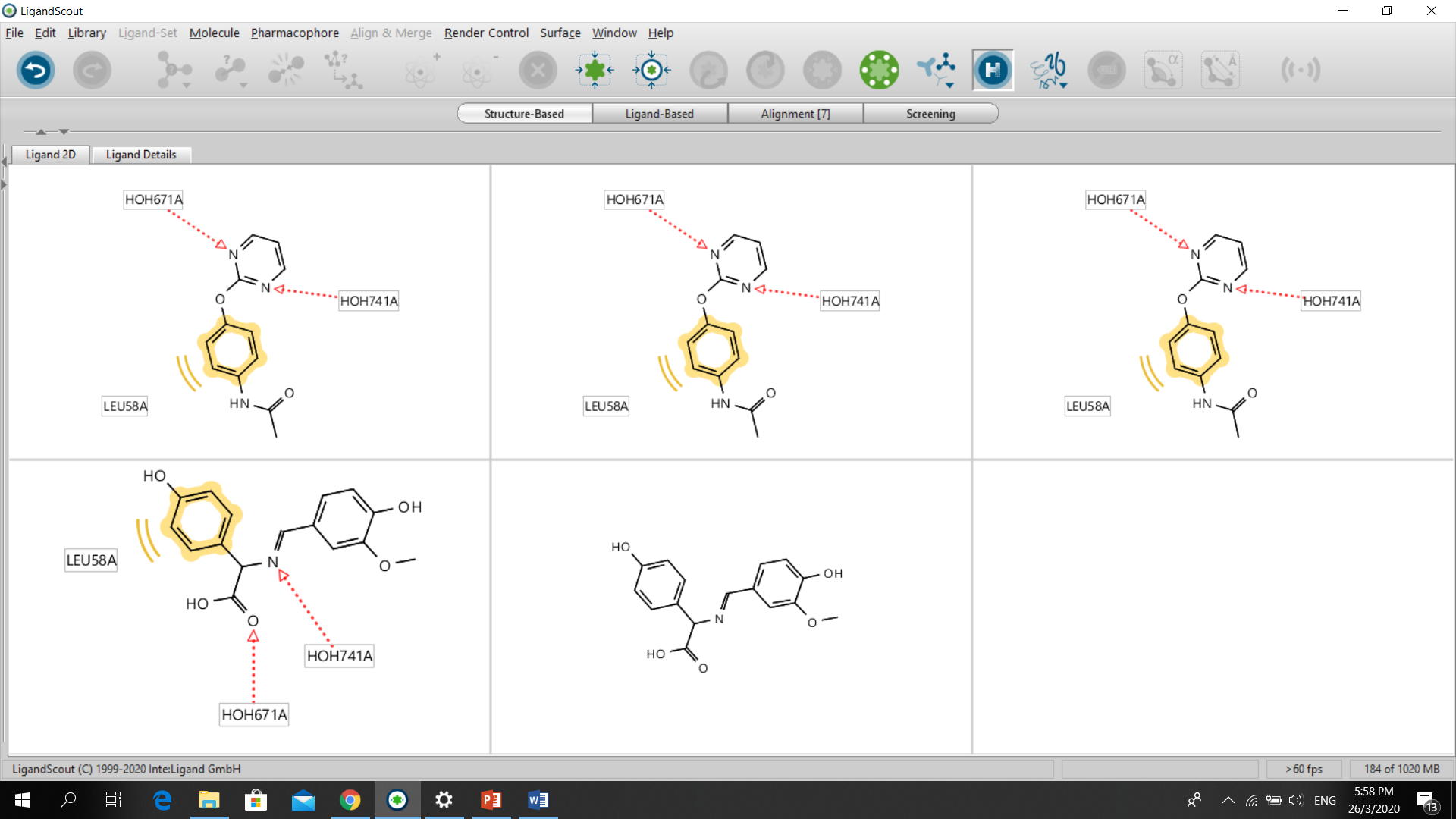 |
| **(9)** | 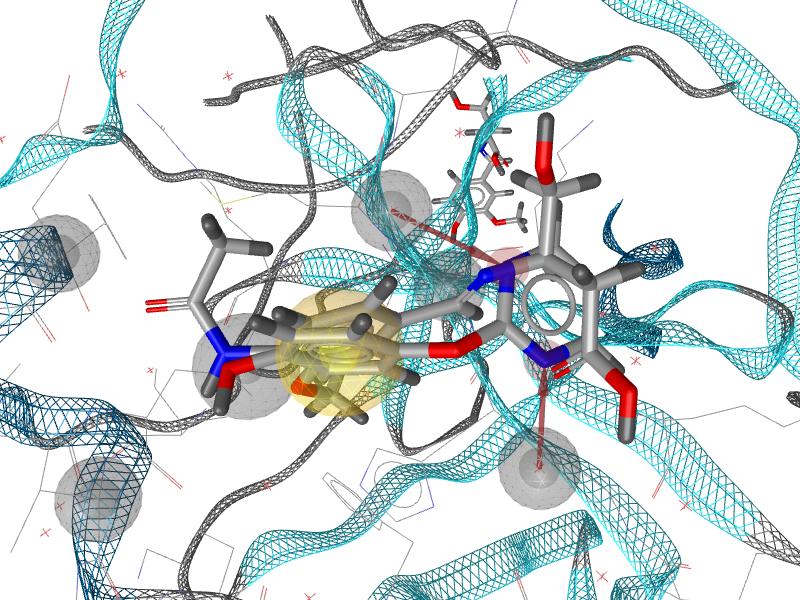  Alignment score: 37.69 | 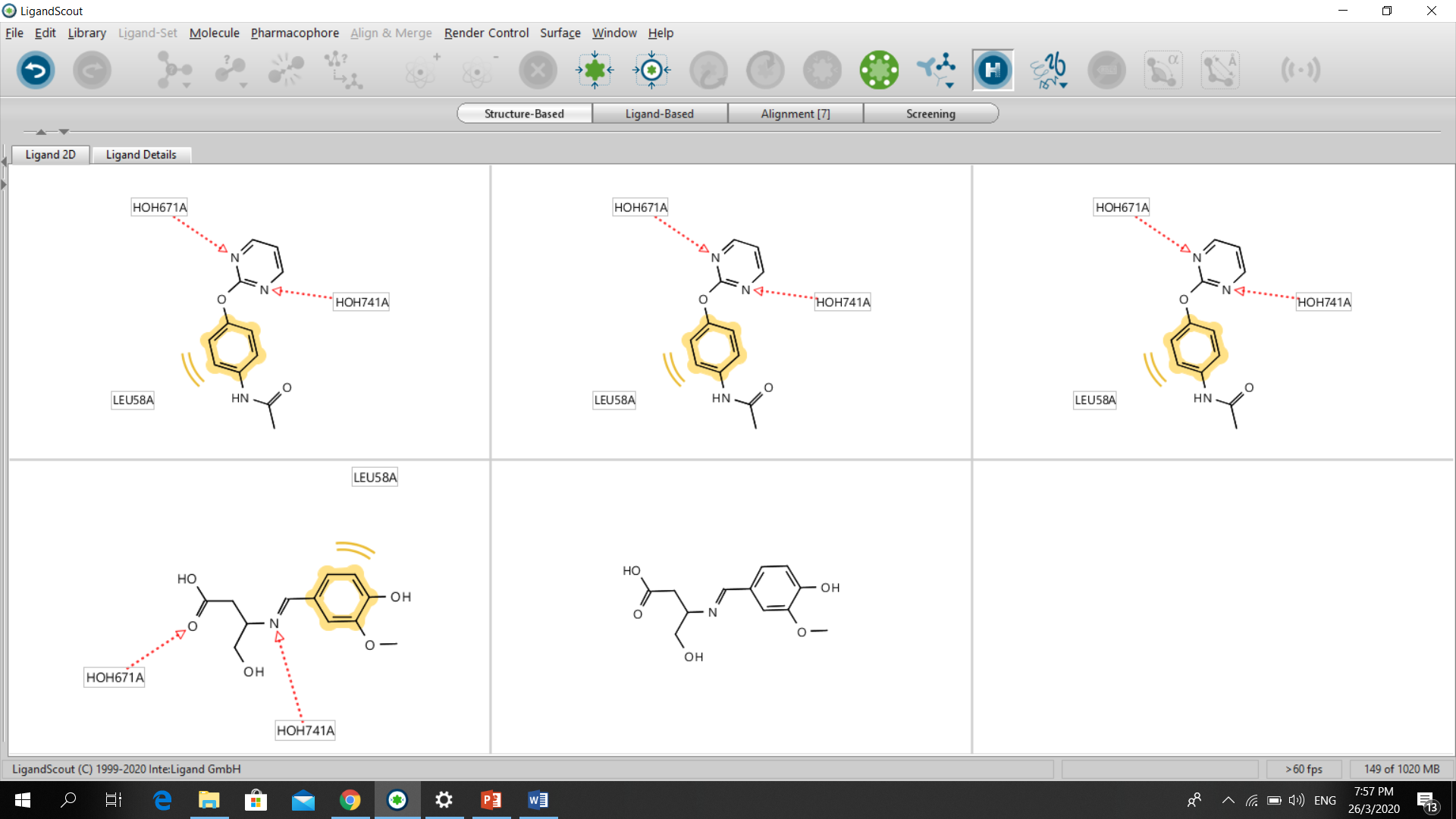 |
| **(10)** | 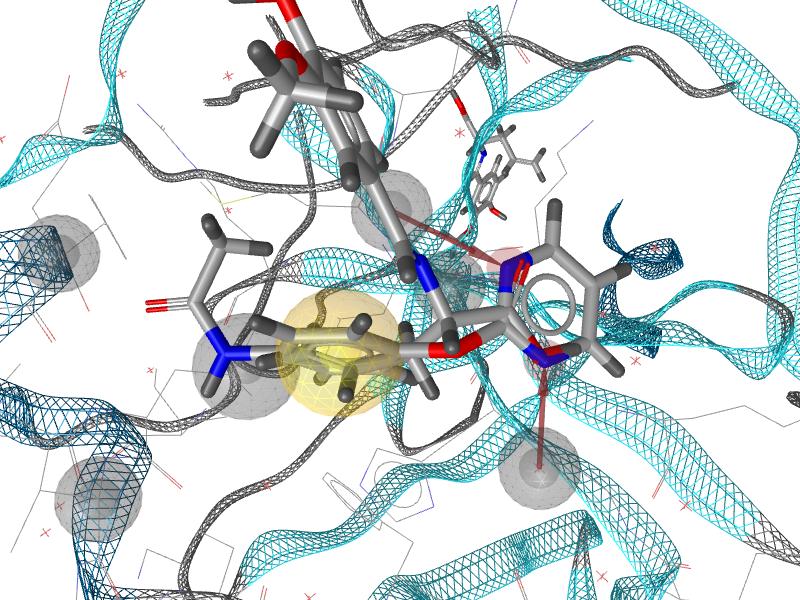  Alignment score: 38.06 | 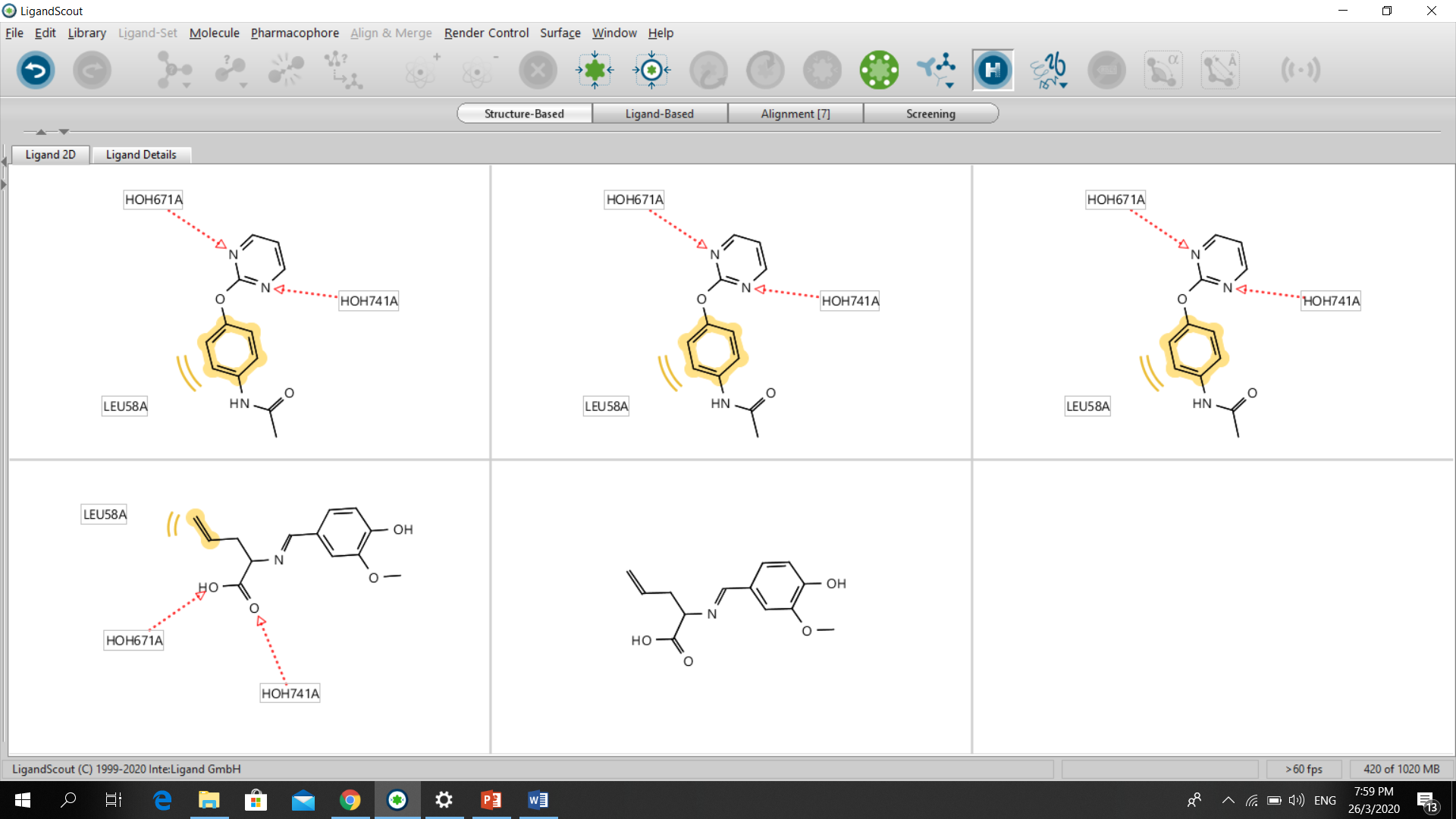 |
| **(11)** | 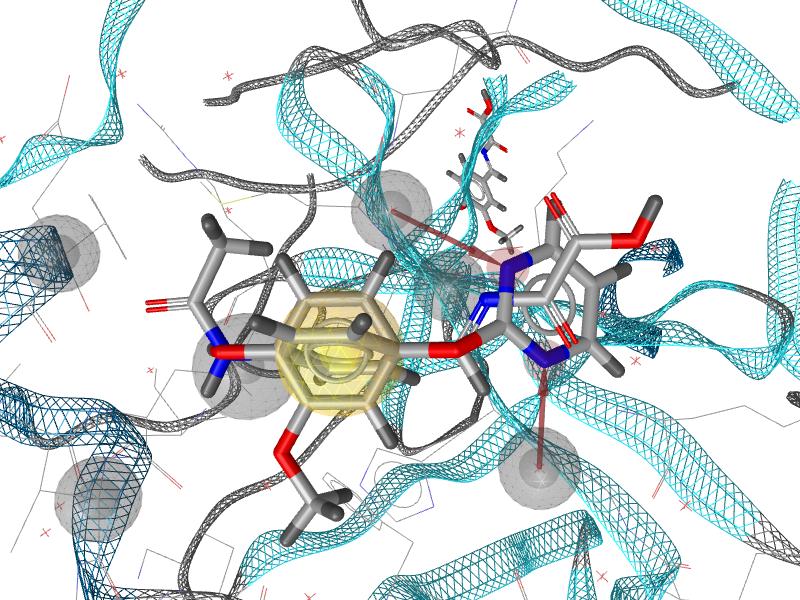  Alignment score: 36.98 | 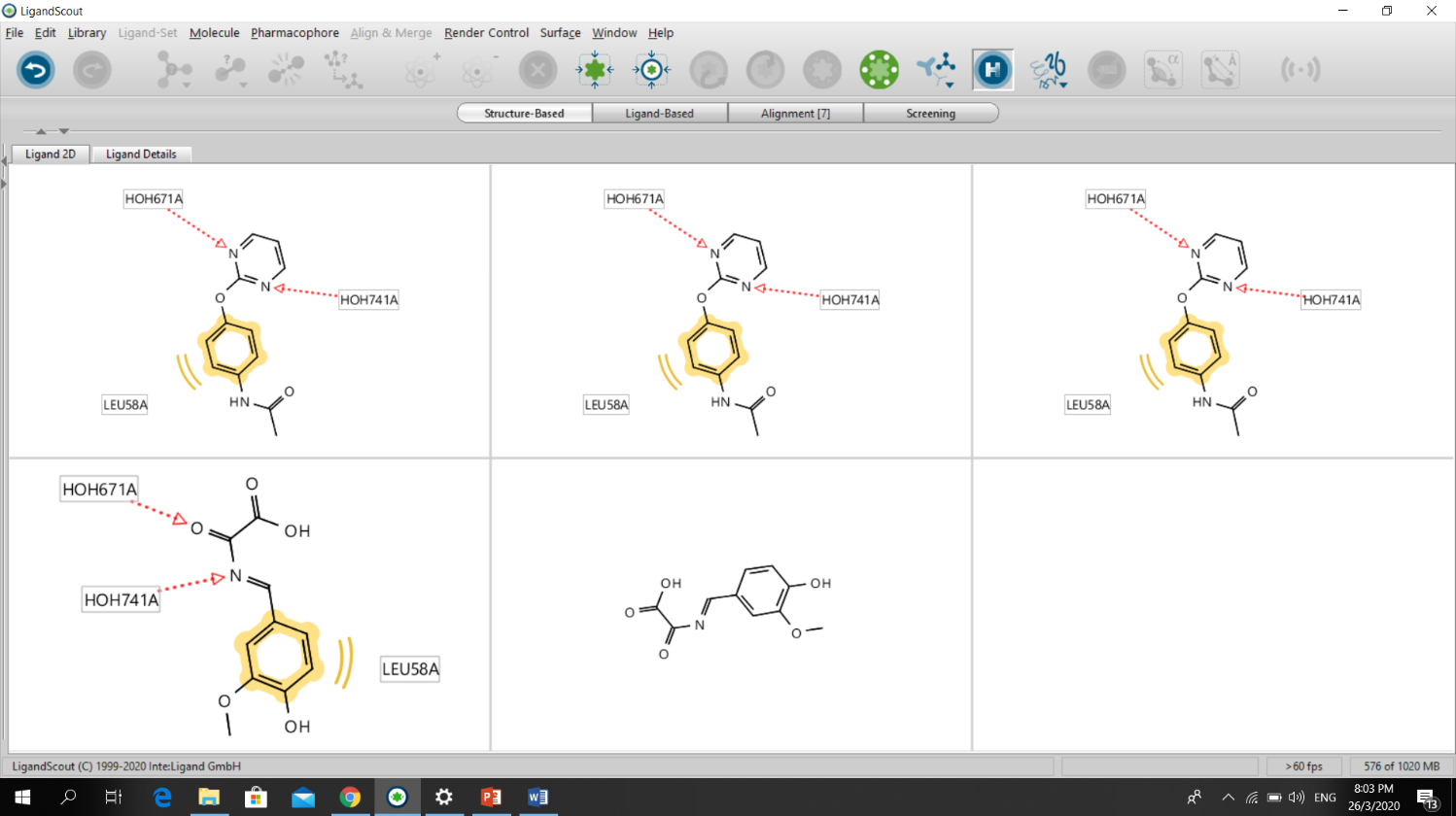 |
| **(12)** | 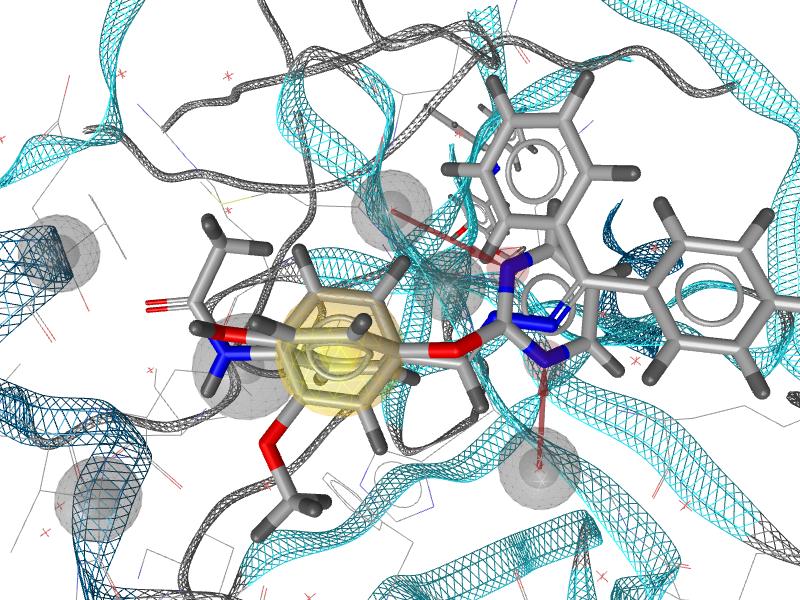  Alignment score: 36.41 | 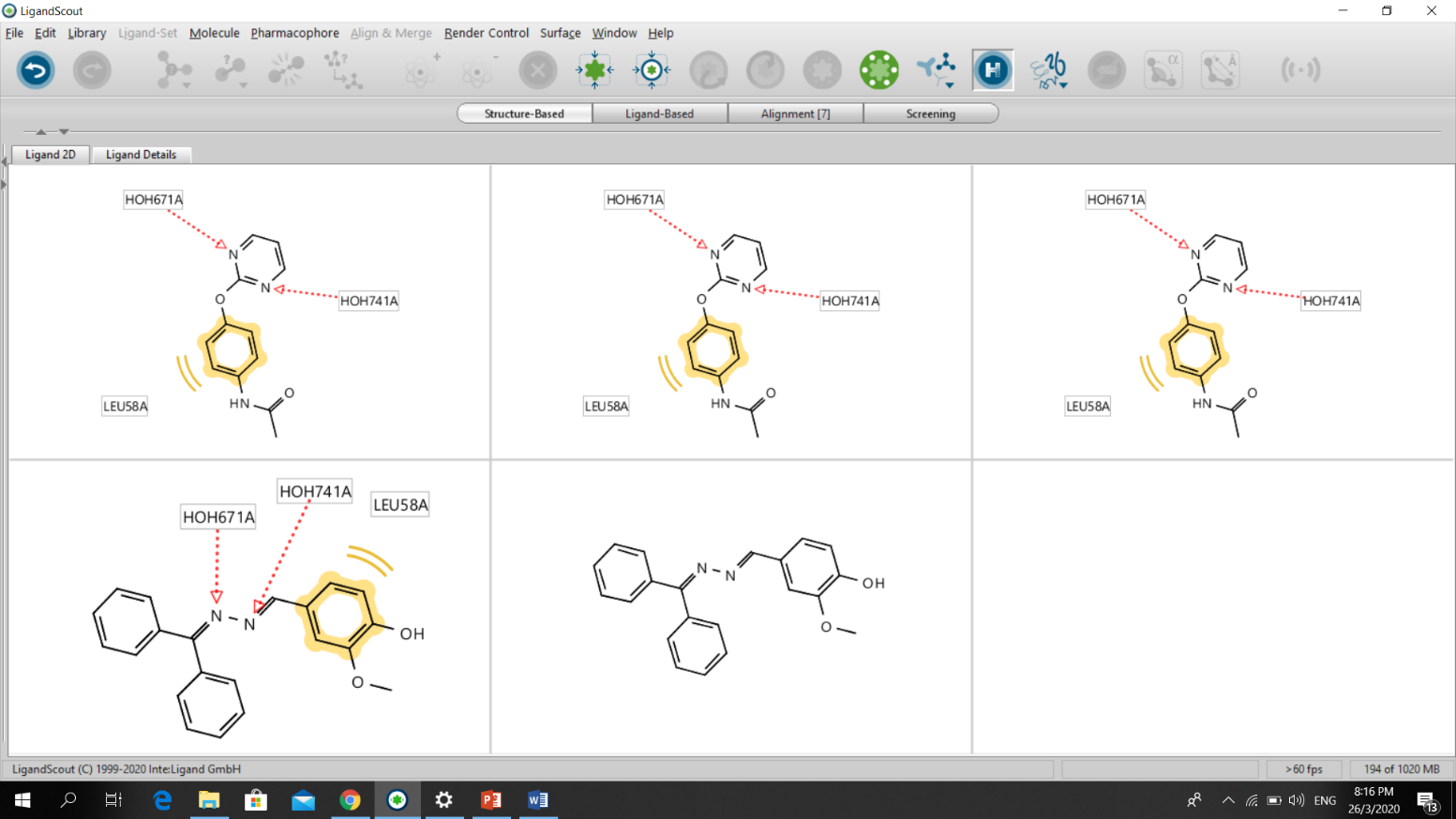 |

**Table S4: Matching features of favipiravir, chloroquine and hydroxychloroquine in the active sites of 5REX and 5RFZ**

| **Compounds** | **PDB code** | |
| --- | --- | --- |
|  | **5REX** | **5RFZ** |
|  | **PCM-0102287**  **1-{4-[(naphthalen-1-yl)methyl]piperazin-1-yl}ethan-1-one in the active site**  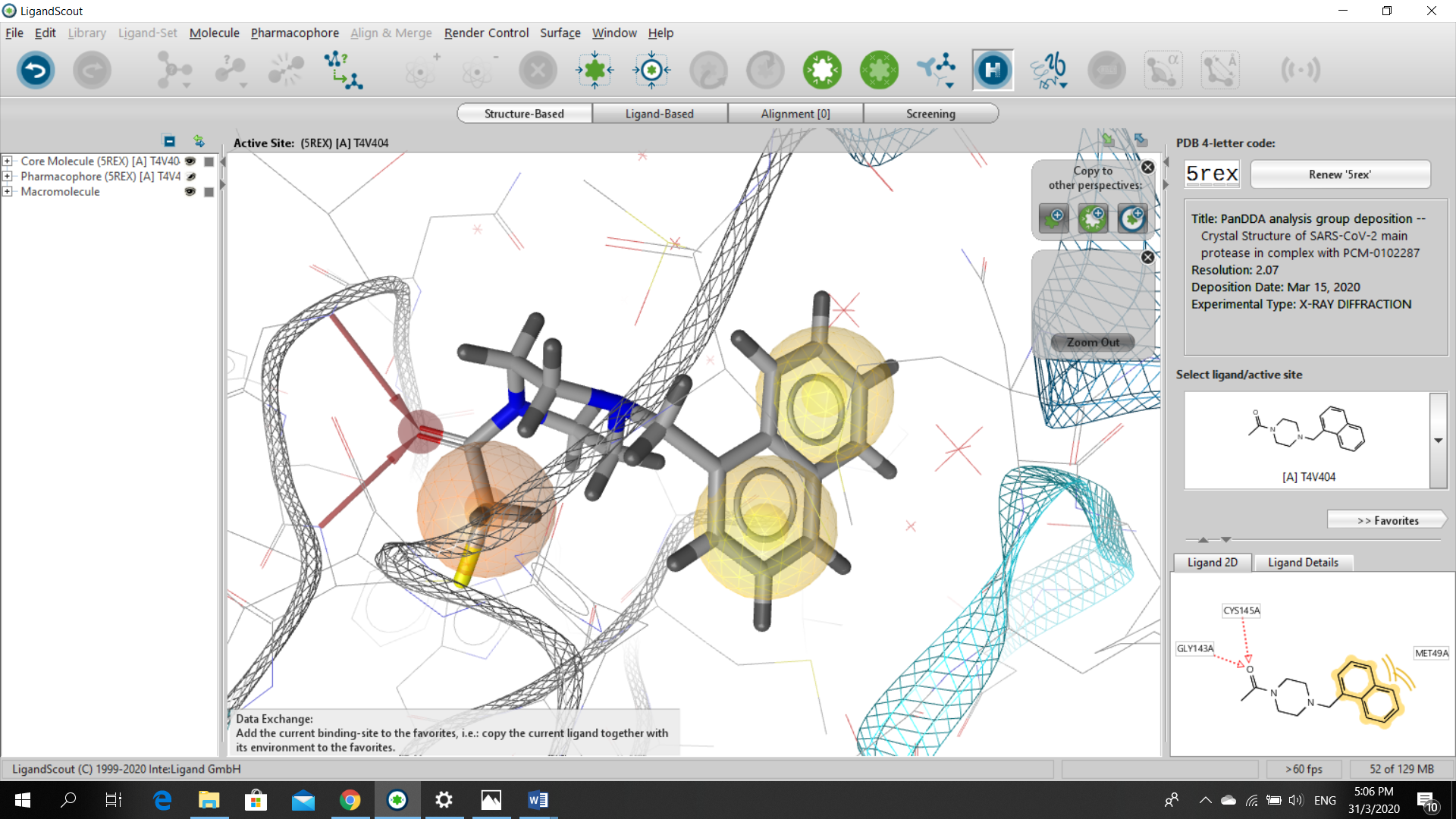  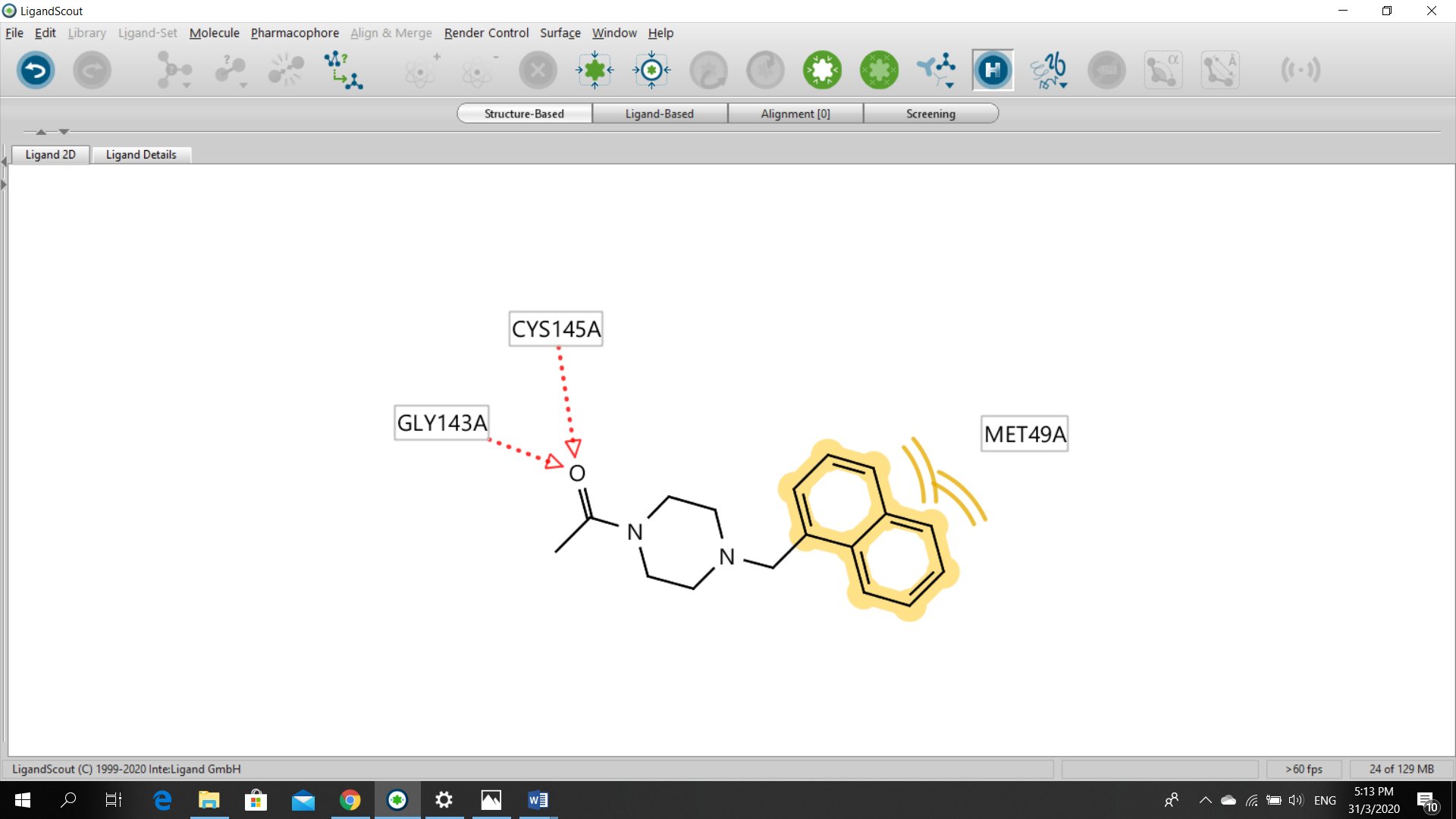 | **PCM-0102274**  **N-(2-chloropyridin-3-yl)acetamide in the active site**  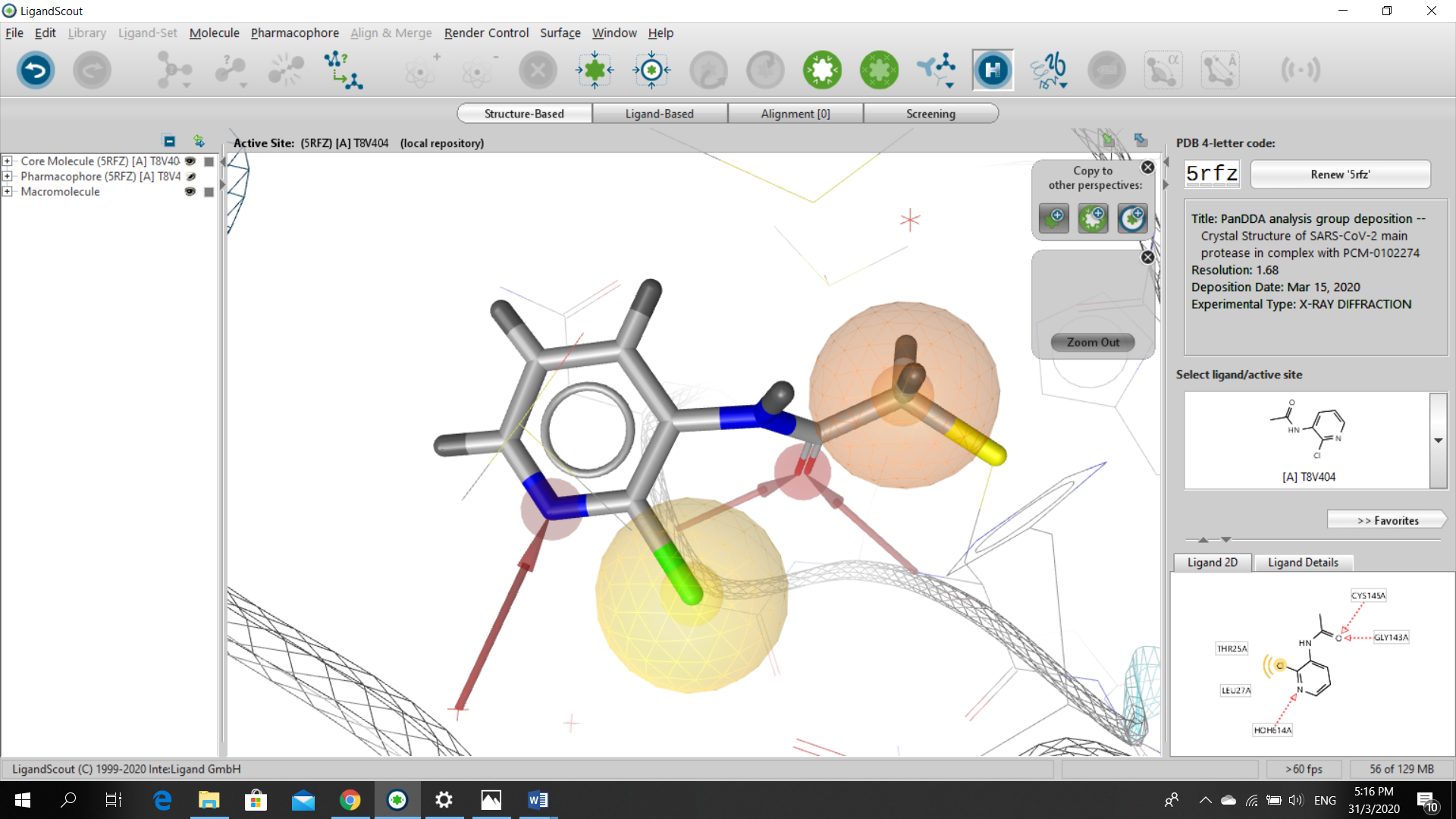  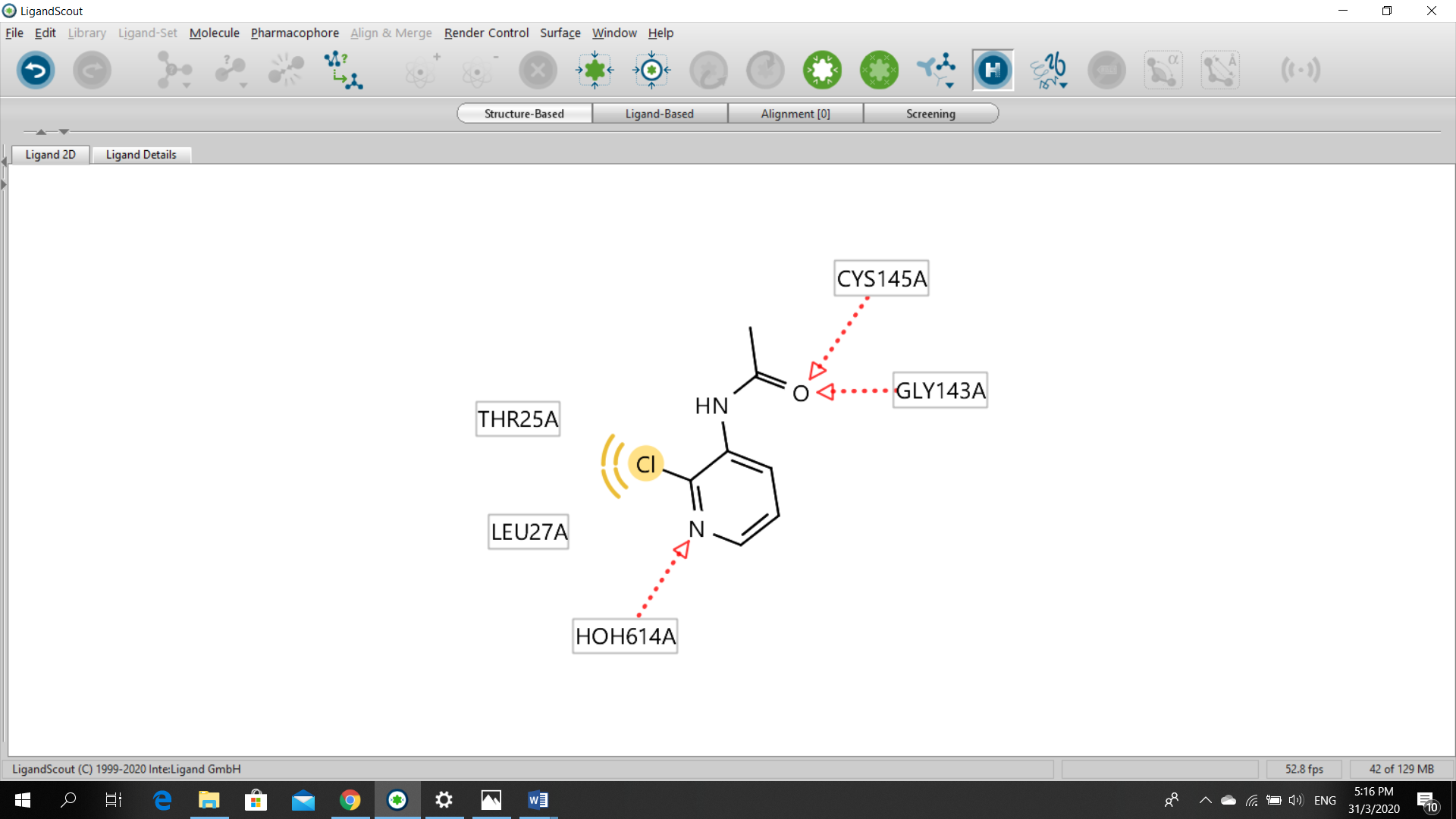 |
| **Favipiravir** |  | 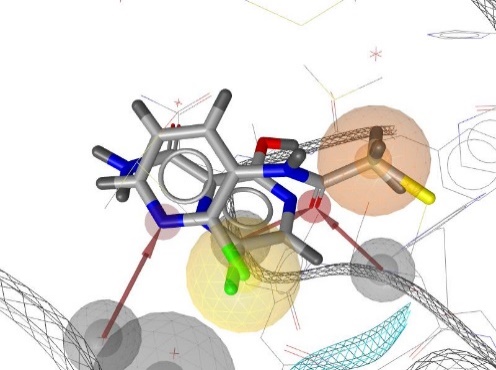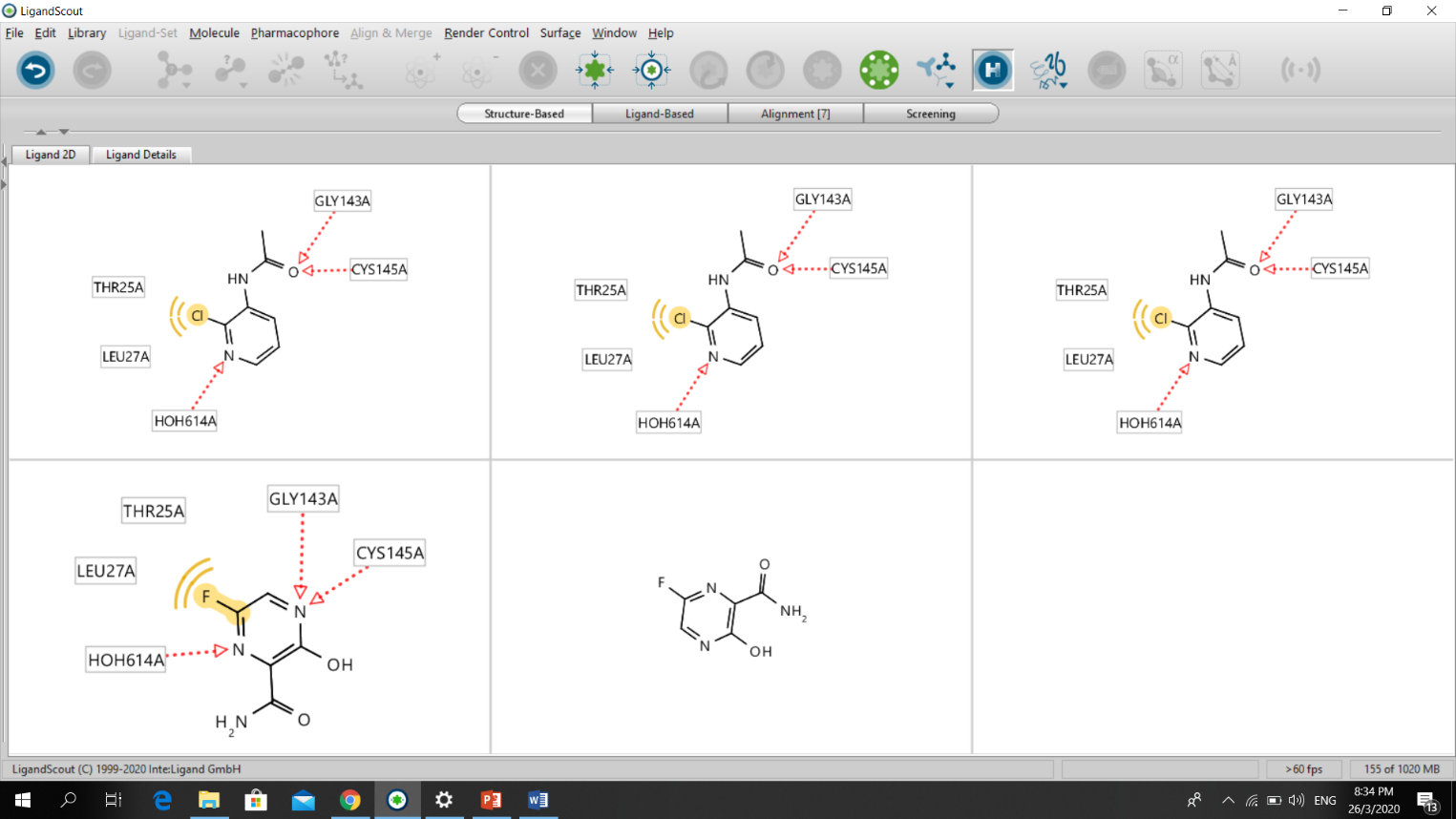 |
| **Chloroquine** | 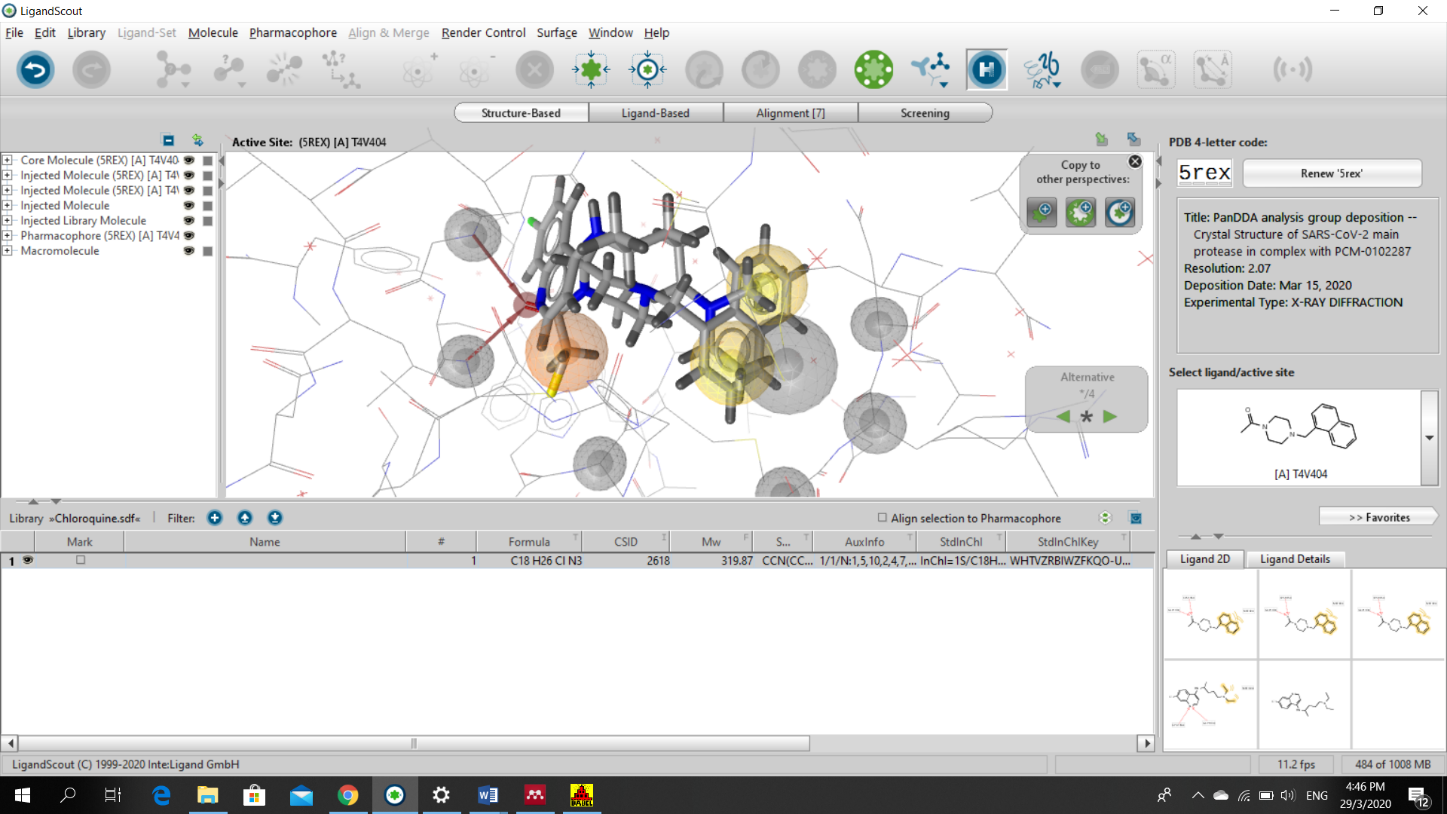 |  |
| **Hydroxychloroquine** |  |  |
